# Madecassic Acid—A New Scaffold for Highly Cytotoxic Agents

**DOI:** 10.3390/ijms23084362

**Published:** 2022-04-14

**Authors:** Oliver Kraft, Ann-Kathrin Hartmann, Sophie Hoenke, Immo Serbian, René Csuk

**Affiliations:** Organic Chemistry, Martin-Luther-University Halle-Wittenberg, Kurt-Mothes-Str. 2, D-06120 Halle (Saale), Germany; oliver.kraft@chemie.uni-halle.de (O.K.); ak.hartmann@gmx.de (A.-K.H.); sophie.hoenke@chemie.uni-halle.de (S.H.); immoserbian@gmail.com (I.S.)

**Keywords:** madecassic acid, gypsogenin, hederagenin, cytotoxicity, mitocan

## Abstract

Due to their manifold biological activities, natural products such as triterpenoids have advanced to represent excellent leading structures for the development of new drugs. For this reason, we focused on the syntheses and cytotoxic evaluation of derivatives obtained from gypsogenin, hederagenin, and madecassic acid, cytotoxicity increased—by and large—from the parent compounds to their acetates. Another increase in cytotoxicity was observed for the acetylated amides (phenyl, benzyl, piperazinyl, and homopiperazinyl), but a superior cytotoxicity was observed for the corresponding rhodamine B conjugates derived from the (homo)-piperazinyl amides. In particular, a madecassic acid homopiperazinyl rhodamine B conjugate **24** held excellent cytotoxicity and selectivity for several human tumor cell lines. Thus, this compound was more than 10,000 times more cytotoxic than parent madecassic acid for A2780 ovarian cancer cells. We assume that the presence of an additional hydroxyl group at position C–6 in derivatives of madecassic, as well as the (2α, 3β) configuration of the acetates in ring A, had a beneficial effect onto the cytotoxicity of the conjugates, as well as onto tumor/non-tumor cell selectivity.

## 1. Introduction

Despite all efforts and tremendous achievements in the fight against cancer, in 2020 10 million people worldwide died as a consequence of this disease [1], and 19.3 million new cases were reported by the International Agency for Research on Cancer [2]. Many chemotherapeutic agents hitherto used for the therapy of cancer are natural products or derived from them. Several compounds derived from pentacyclic triterpenes were successfully encountered as highly cytotoxic as well as exhibiting pronounced tumor-selectivity [3,4,5,6,7]. A closer look into this class of compounds, however, revealed that research was mainly focused on derivatives of betulinic, ursolic, and oleanolic acid and—to a minor extent—of glycyrrhetinic acid [8,9,10,11,12,13,14,15]. The number of investigations dealing with cytotoxic agents derived from gypsogenin, hederagenin, or madecassic acid, however, remained small over many years [16,17,18,19]. This may be due to the fact that the availability of these compounds is considered to be worse than that of, for example, betulinic acid [20,21,22]. This has changed as gypsogenin can now be obtained in good yields from the corresponding saponin (which is available in large technical quantities). Hederagenin was previously only available from extracts from the pericarp of the soap tree [23,24]. However, it is now even more convenient, obtained by a partial synthesis from also readily available gypsogenin [23]. Furthermore, hederagenin and gypsogenin can be interconverted into each other by a short sequence and in good yields [23].

Recently, we were able to show that acetylated pentacyclic triterpenoid amides are highly cytotoxic for many human tumor cell lines [24,25,26,27], while parent triterpenoic acids possess low cytotoxicity. The cytotoxicity of these compounds, however, intensifies upon acetylation and amidation, and even more by linking them with rhodamine B [28]. Thus, the synthesis of different rhodamine B conjugates has moved into the focus of scientific interest [25,26,27,29]. For example, benzyl-, piperazinyl-, and homopiperazinyl-amides and their resulting rhodamine B conjugates show cytotoxic effects even in nanomolar concentration. The presence of an extra cationic moiety such as a rhodamine B scaffold enhances their cytotoxic activity tremendously. However, many of these conjugates were also less cytotoxic to non-malignant fibroblasts (NIH 3T3) [26,27,28,30]. As a consequence of these promising results, and to have a closer look into this class of compounds, gypsogenin and hederagenin were chosen as starting materials for further studies. For comparison, madecassic acid was also included to investigate the possible influence of the presence of extra hydroxyl groups onto cytotoxic activity and selectivity. During our work, different acetylated triterpenoic derivatives modified at their carboxylic group were accessed, such as substituted benzyl, piperazinyl, and homopiperazinyl amides which were subsequently linked at their distal amino group as an amide to a rhodamine B moiety. Previous studies showed an increase in malignant cells’ membrane potential in relation to non-malignant cells. Thus, cationic compounds such as rhodamine B conjugates accumulate in these areas and cause selective cell death [31,32]. This is regarded as the main reason for the selective cell death of tumor cells upon incubation with the rhodamine B conjugates.

Our main objective was to find out whether triterpene amides, as well as spacered triterpene rhodamine conjugates, exhibit good cytotoxicity when the pentacyclic triterpene skeleton holds a vicinal hydroxyl groups in ring A, and optionally another hydroxyl group at another position. These requirements are fulfilled, for example, by the compounds gypsogenin, hederagenin, or madecassic acid. This should also allow a comparison with the conjugates of ursolic, oleanolic, and maslinic acid presented earlier.

Thus, a set of novel terpenoic amides and their rhodamine B conjugates was synthesized, and the cytotoxicity of the compounds was determined by sulforhodamine B (SRB) assays.

## 2. Results and Discussion

### 2.1. Chemistry

**Scheme 1:**
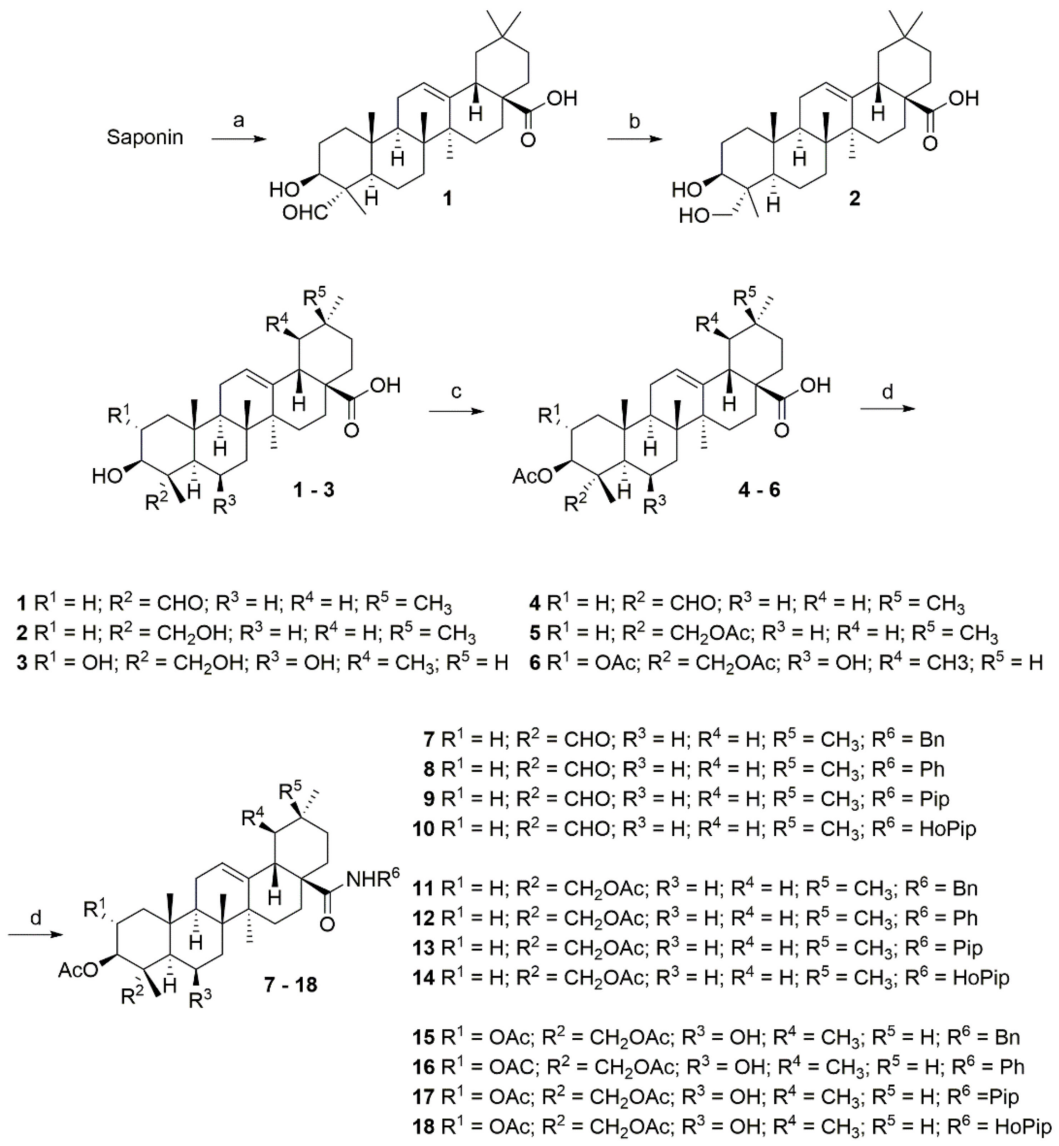
Reactions and conditions: (**a**) aq. HCl, 60 °C, 5 d, 2%; (**b**) NaBH_4_, THF/MeOH, 0 °C, 2 h, 72%; (**c**) Ac2O, NEt_3_, DMAP (cat.), DCM, 23 °C, 1 h, **4** (79%), **5** (94%), **6** (78%); (**d**) (COCl)_2_, DCM, DMF (cat.), then amine, 23 °C, 1 h; → **7** (81%), **8** (65%), **9** (64%), **10** (83%), **11** (52%), **12** (98%), **13** (80%), **14** (41%), **15** (41%), **16** (60%), **17** (41%), and **18** (81%).

Hydrolysis of Gypsophila saponin provided gypsogenin as previously reported (**1**, Scheme 1), whose reduction with NaBH_4_ gave hederagenin (**2**) in 78% isolated yield [23]; madecassic acid (**3**) was purchased from different local suppliers.

Acetylation of **1**–**3** gave acetates **4**–**6,** respectively [16,24,27]. These compounds were each treated with oxalyl chloride followed by the addition of benzylamine, aniline, piperazine, or homopiperazine to furnish amides **7**–**18**, respectively.

**Scheme 2:**
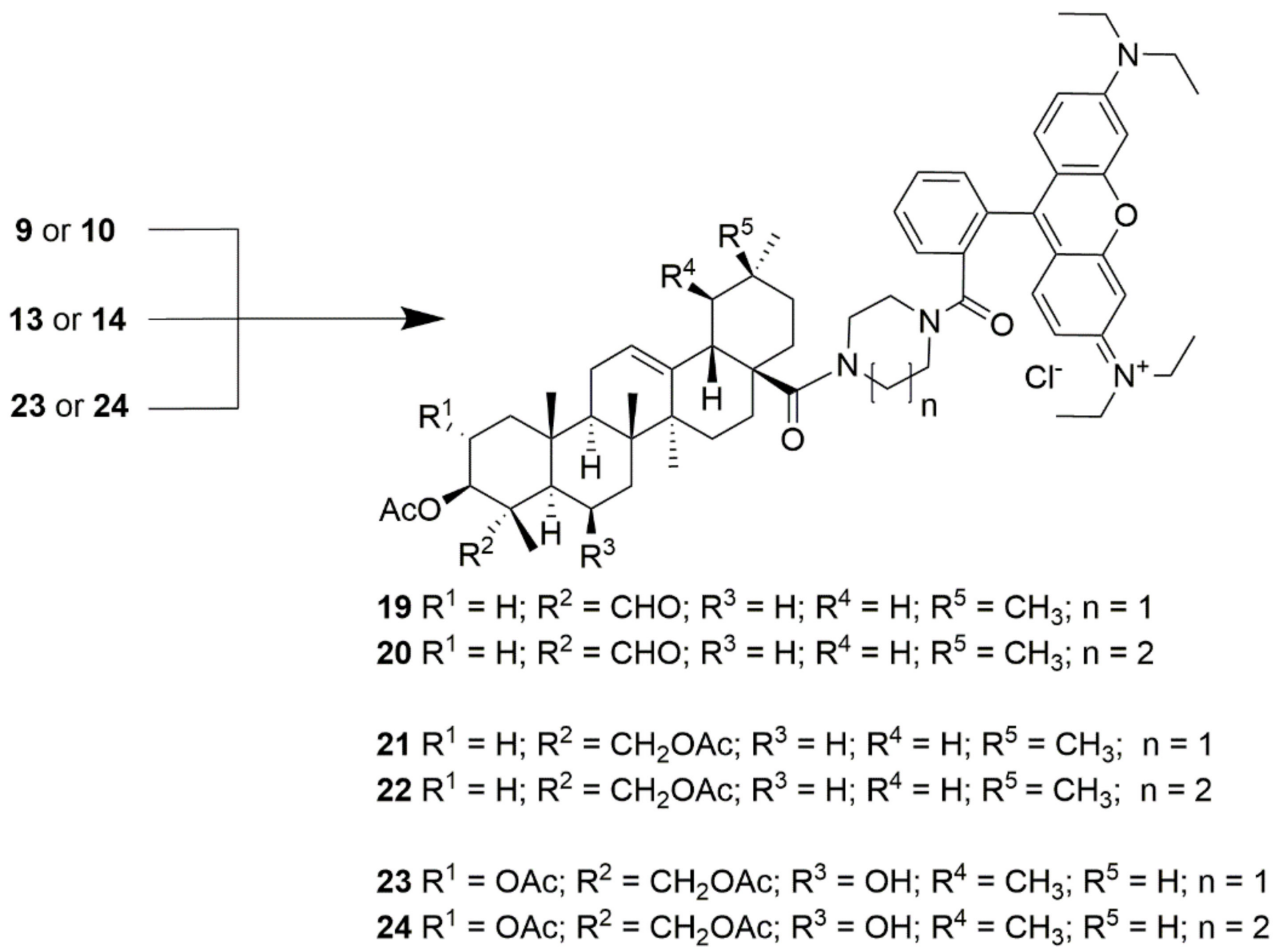
Reactions and conditions: rhodamine B, (COCl)_2_, DCM, NEt_3_ (cat.), DMF (cat.), 23 °C, 1 h, then starting material, 0 °C → 23 °C, 1 h; → **19** (64%), **20** (56%), **21** (47%), **22** (31%), **23** (56%), and **24** (48%).

Coupling amides **9**, **10**, **13**, **14**, **17**, and **18** with rhodamine B (after having being activated in situ with oxalyl chloride) gave the rhodamine B conjugates **19**–**24**, respectively (Scheme 2) [33].

Thus, compounds **19**–**24** can be regarded as analogs of previously reported piperazinyl and homopiperazinyl rhodamine B conjugates [26,33], whereas **7**, **8**, **11**, **12**, **15**, and **16** are structurally similar to the benzyl- and phenylamides previously reported by Siewert et al. [30] and Kaminskyy et al. [34] However, compounds of this study differ from those previously synthesized from ursolic, oleanolic, glycyrrhetinic, betulinic, or platanic acid inasmuch as the conjugates of this study hold (an) extra hydroxyl group(s) (protected, in part, as an acetate).

### 2.2. Biological Evaluation

To assess their cytotoxicity parent, triterpenoic acids **1**–**3**, their acetates **4**–**6**, the amides **7**–**18,** as well as the respective rhodamine B conjugates **19**–**24** were subjected to SRB assays employing several human tumor cell lines (A375, HT29, MCF-7, A2780, and HeLa) as well as non-malignant fibroblasts (NIH 3T3). The results from these assays are compiled in Table 1. The EC_50_ values in μM from SRB assays were determined after 72 h of treatment, and the values are averaged from three independent experiments performed each in triplicate, confidence interval CI = 95%; mean ± standard mean error). 

As a result, and as expected, the parent compounds **1**–**3** (holding unsubstituted hydroxyl groups) were—by and large—less cytotoxic than their corresponding acetates **4**–**6**. The former compounds showed some cytotoxicity for all human tumor cell lines and moderate selectivity. 

The latter compounds, however, holding one or more acetyl groups at positions 2, 3, and 23, showed an increased cytotoxic activity for all cancer lines, with EC_50_ values as low as EC_50_ = 3.50 µM (for A375 and compound **6**).

Transforming the carboxyl group into an amide led to compounds of increased cytotoxic effects onto the tumor cells. Benzylamides (**7**, **11**, and **15**), phenyl amides (**8**, **12**, and **16**), piperazinyl amides (**9**, **13**, and **17**) and homopiperazinyl amides (**10**, **14**, and **18**) held high cytotoxicity and moderate selectivity. In this series of compounds, hederagenin derived phenyl amide **12** was most cytotoxic with an EC_50_ value of 0.66 μM for A375 tumor cells. Comparing the cytotoxic effects of phenyl- and benzyl amides conjugates with those measured for the corresponding piperazinyl and homopiperazinyl analogues showed the former of higher cytotoxicity but also of an improved selectivity (malignant vs. non-malignant cells). 

Rhodamine B analogues **19**–**24** showed both an increased cytotoxicity but also improved selectivity. The lowest EC_50_ value was determined for **24** with EC_50_ = 0.0029 ± 0.0005 for A2780 ovarian tumor cells. 

The selectivity between malignant cells and the non-malignant cells was also affected by the kind of spacer linking the triterpene with the rhodamine B moiety. Thereby, a significant difference in cytotoxicity was detected between compounds holding a homopiperazinyl (**20**, **22**, and **24**) instead of piperazinyl spacer (**19**, **21**, and **23**).

As a result, compound **24**, the most active as well as selective compound with homopiperazinyl spacer, was nearly 10,000 times more cytotoxic than parent madecassic acid.

**Figure 1:**
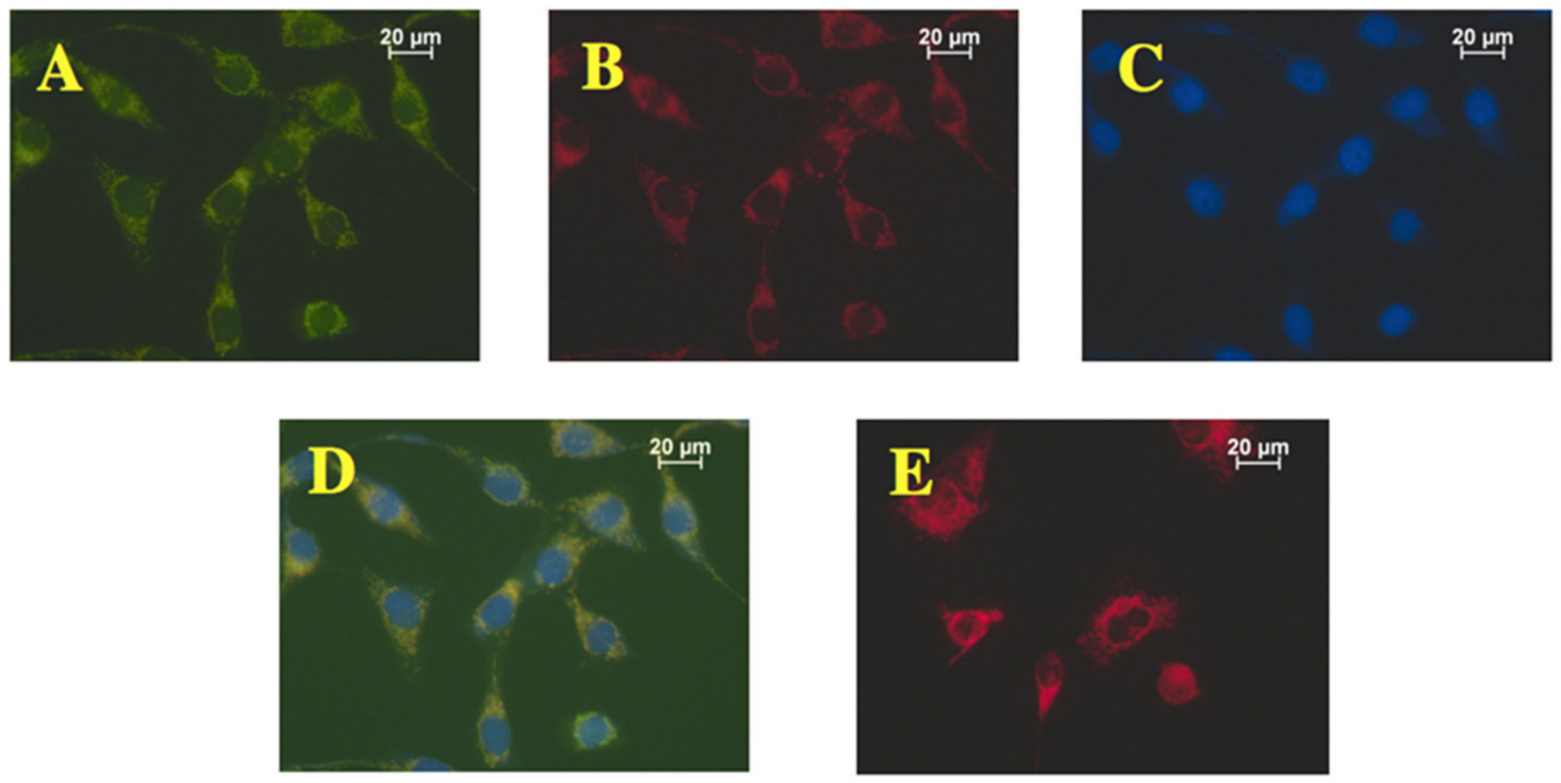
Staining experiments: A375 cells, 48 h; (**A**): control; (**B**): in the presence of **24**; (**C**): Hoechst 33,342 staining; (**D**): merged (Hoechst 33,342, control); and (**E**): in the presence of rhodamine 123.

The mode of action—as previously shown for several triterpene-rhodamine B conjugates—is to act as mitocans. To demonstrate this also for **24**—the most cytotoxic compound of this study—A375 cells were stained with acridine orange (AO), rhodamine 123, and Hoechst 33,342. Thereby, Hoechst 33,342 binds selectively to DNA while rhodamine 123 is usually applied for dying mitochondria. The results of these staining experiments are depicted in Figure 1 and prove that **24** also acts as a mitocan, as this compound is localized within the cells exactly in the same area as rhodamine 123, while a location in the nucleus can be ruled out from the staining experiments using Hoechst 33,342.

The influence of an extra hydroxyl group in ring B can best be seen from the cytotoxicity of the analogs of madecassic acid. The results from the SRB assays showed compounds **3**, **6**, **15**, **16**, **17**, **18**, **23**, and **24** as cytotoxic with EC_50_ values as low as 0.015 ± 0.001 μM; for compound **24** and A2780 cells, a selectivity index SI_EC50, NIH 3T3/EC50, A2780_ of approximately 20 can be calculated. From these results, we conclude that the presence of an additional hydroxyl group at position C-6 in the triterpenoid skeleton might be responsible for a significantly improved cytotoxicity. 

As compounds holding two acetyl groups in ring A with (2α, 3β) configuration (such as **24** or the analogues prepared earlier from maslinic or tormentic acid) are clearly more cytotoxic than those bearing only one acetyl group (such as oleanolic and ursolic, but also glycyrrhetinic, betulinic, or platanic acid), this confirms our original working hypothesis. At present, triterpenes with a larger number of hydroxyl or acetyl groups are being converted to the corresponding spacered rhodamine B conjugates in our laboratories in order to verify whether the trend shown here has general validity.

## 3. Materials and Methods

### 3.1. General

A detailed description of materials and methods can be found in the Supplementary Materials File. For biological screening, the cell lines were obtained from ATCC: A375 (CRL-1619), HT29 (HTB-38), MCF7 (HTP-22), A2780 (HTP-77), HeLa (CCL-2), and NIH 3T3 (CRL-1573) ^13^C NMR spectra (Supplementary Materials) were recorded as APT spectra showing CH and CH_3_ groups as positive signals and CH_2_ groups and quaternary carbons as negative signals.

### 3.2. General Procedure for the Acetylation of Triterpenoic Acids (GPA)

The triterpenoic acids **1**–**3** (1 eq.) were each dissolved in dry DCM (20 mL), treated with TEA (4.5 eq.), DMAP (cat.), and acetic anhydride (4.0 eq.). The mixture was stirred at room temperature for 24 h; for work-up, aq. ammonium hydroxide (7 m, 0.5 mL) was added. After 30 min, the mixture was diluted with Et_2_O (250 mL), washed with HCl (0.1 m, 1 × 250 mL), water (2 × 250 mL), and brine (1 × 125 mL), dried with MgSO_4_, and the organic phase was concentrated under reduced pressure. The crude product was subjected to column chromatography (SiO_2_, *n*-hexane/ethyl acetate) to afford compounds **4**–**6**, each as a colorless solid.

### 3.3. General Procedure for the Synthesis of of Triterpenoic Amides 7–18 (GPB)

To a solution of **4**–**6** (1 eq.) in dry DCM (10 mL), oxalyl chloride (4 eq.), DMF (0.24 eq.), and TEA (0.24 eq.) were added. After stirring for 2 h at room temperature, the solvent was removed under reduced pressure, re-evaporated with DCM (3 × 10 mL), and the residue was dissolved in dry DCM (10 mL). A solution of the respective amine or diamine (3 eq.) in dry DCM, TEA (1 eq.), and DMAP (cat.) was added, and stirring at room temperature was continued until completion of the reaction (as indicated by TLC). The solvent was removed under reduced pressure, and the crude product was subjected to column chromatography to yield compounds **7**–**18**.

### 3.4. General Procedure for Synthesis of Rhodamine B Conjugates 19–24 (GPC)

A solution of rhodamine B (1.5 eq.), oxalyl chloride (6 eq.), DMF (0.2 eq.), and TEA (0.2 eq.) in dry DCM (30 mL) was stirred at ambient temperature for 3 h. The solvent was removed under reduced pressure, re-evaporated with dry DCM (3 × 30 mL), and the residue was dissolved in dry DCM (30 mL). To this solution, compounds **9**, **10**, **13**, **14**, **17**, or **18** (1 eq.), TEA (1.5 eq.), and DMAP (cat.) were added. After stirring for 1 h, the solvent was removed under reduced pressure, and the resulting solid was subjected to column chromatography (SiO_2_, chloroform/methanol, 9:1 or *n*-hexane/ethyl acetate, 7:3) to yield compounds **19**–**24**, each as a purple solid.

### 3.5. (3β,4α) 3-Hydroxy-23-oxoolean-12-en-28-oic Acid (**1**) [639-14-5]

The saponin of *Gypsophila* (300 g; Dr. H. Schmittmann GmbH, Velbert, Germany) was treated with aq. hydrochloric acid (15%, 1000 mL) at 60 °C for 120 h. The solvent was removed under reduced pressure, the residue was air dried, grounded, and extracted with ethyl acetate (2 L) under reflux. The solvent was evaporated, and the dark brown residue was subjected to column chromatography (SiO_2_, *n*-hexane/ethyl acetate, 7:3) to yield **1** (6.2 g) as a colorless solid; m.p.: 275–278 °C (lit.: [24] 274–276 °C); ${\left[\alpha \right]}_{D}^{20}$ = +89.5° (*c* = 0.5, EtOH), [lit.: [35] ${\left[\alpha \right]}_{D}^{20}$ = +91.4° (*c* = 1.45, EtOH)]; and MS (ESI): *m/z* (%) 491.0 ([M + Na]^+^, 100).

### 3.6. (3β,4α) 3,23-Dihydroxyolean-12-en-28-oic Acid (**2**) [465-99-6]

To an ice-cold solution of **1** (2.0 g, 4.23 mmol) in THF/MeOH (100 mL, 1:1) NaBH_4_ (193 mg, 5.1 mmol) was added, and the reaction was stirred for 2 h, quenched with aq. hydrochloric acid (2 m, 50 mL) and extracted with chloroform (3 × 50 mL). The combined organic phases were dried over MgSO_4_, the solvent was removed under reduced pressure, and the crude product was subjected by column chromatography (SiO_2_, *n*-hexane/ethyl acetate, 7:3) to yield **2** (1.4 g, 72%) as a colorless solid; m.p.: 331–334 °C (lit.: [23] 332–335 °C); ${\left[\alpha \right]}_{D}^{20}$ = +80.3° (*c* = 0.52, pyridine), [lit.: [36] ${\left[\alpha \right]}_{D}^{20}$ = +81.2° (pyridine)]; and MS (ESI): *m*/*z* (%) 471.1 ([M-H]^−^, 100).

### 3.7. Madecassic Acid (**3**)

This compound was obtained from different local suppliers and used as received.

### 3.8. (3β,4α) 3-(Acetyloxy)-23-oxoolean-12-en-28-oic Acid (**4**) [27706-38-3]

Following GPA from **1** (1500 mg, 3.18 mmol) followed by column chromatography (SiO_2_, *n*-hexane/ethyl acetate, 8:2), **4** (2.40 mmol, 79%) was obtained as a colorless solid; m.p.: 175–178 °C (lit.: [24] 177–178 °C); ${\left[\alpha \right]}_{D}^{20}$ = +77° (*c* = 0.6, CHCl_3_), [lit.: [37] ${\left[\alpha \right]}_{D}^{20}$ = +78° (*c* = 0.6, CHCl_3_)]; and MS (ESI): *m*/*z* (%) 511.6 ([M−H]^−^, 90).

### 3.9. (3β,4α) 3,23-Bis(acetyloxy)olean-12-en-28-oic Acid (5) [5672-32-2]

Following GPA from **2** (2000 mg, 4.23 mmol) followed by column chromatography (SiO_2_, *n*-hexane/ethyl acetate, 8:2), **5** (1880 mg, 94%) was obtained as a colorless solid; m.p.: 185–189 °C (lit.: [38] 173–175 °C); ${\left[\alpha \right]}_{D}^{20}$ = +76.8° (*c* = 0.17, CHCl_3_) [lit.: [39] ${\left[\alpha \right]}_{D}^{20}$ = +78° (CHCl_3_)]; R_F_ = 0.37 (SiO_2_, *n*-hexane/ethyl acetate, 8:2); IR (ATR): ν = 2944*br*, 1738*s*, 1692*s*, 1467*w*, 1366*s*, 1239*vs*, 1040*s*, 771*w* cm^−1^; ^1^H NMR (500 MHz, CDCl_3_): δ = 5.29–5.24 (*m*, 1H, 12–H), 4.79 (*dd*, *J* = 11.6, 4.8 Hz, 1H, 3–H), 3.87 (*d*, *J* = 11.6 Hz, 1H, 23–H_a_), 3.70 (*d*, *J* = 11.6 Hz, 1H, 23–H_b_), 2.82 (*dd*, *J* = 13.9, 4.6 Hz, 1H, 18–H), 2.06 (*s*, 3H, 15–H_a_ + 16–H_a_ + 22–H_a_), 2.04 (*s*, 3H, 34–H), 2.02 (*s*, 3H, 32–H), 1.97 (d*d*, *J* = 13.4, 4.0 Hz, 2H, 11–H_a_ + 11–H_b_), 1.92–1.86 (*m*, 3H, 16–H_b_ + 2–H_a_ + 2–H_b_), 1.79 (*d*, *J* = 4.4 Hz, 1H, 22–H_b_), 1.77 (*d*, *J* = 4.4 Hz, 1H, 7–H_a_), 1.65–1.56 (*m*, 5H, 7–H_b_ + 19–H_a_ + 9–H +1–H_a_ + 5–H), 1.43–1.33 (*m*, 3H, 6–H_a_ + 6 –H_b_ + 21–H_a_), 1.24–1.16 (*m*, 3H, 19–H_b_ + 21–H_b_ + 15–H_b_), 1.12 (*s*, 1H, 1–H_b_) 1.11 (*s*, 3H, 27–H), 0.97 (*s*, 3H, 25–H), 0.92 (*s*, 3H, 29–H), 0.90 (*s*, 3H, 30–H), 0.82 (*s*, 3H, 24–H), 0.74 (*s*, 3H, 26–H) ppm; ^13^C NMR (125 MHz, CDCl_3_): δ = 184.0 (C–28), 171.0 (C–31), 170.7 (C–33), 143.6 (C–13), 122.4 (C–12), 74.5 (C–3), 65.4 (C–23), 52.0 (C–5), 47.7 (C–9), 46.5 (C–17), 45.8 (C–19), 41.5 (C–4), 40.9 (C–14), 40.5 (C–18), 39.3 (C–8), 37.7 (C–1), 36.8 (C–10), 33.8 (C–21), 33.0 (C–30), 32.4 (C–7), 32.2 (C–20), 30.7 (C–15), 27.6 (C–2), 25.8 (C–27), 23.6 (C–29), 23.4 (C–11), 22.9 (C–16) 22.8 (C–22), 21.2 (C–32), 20.9 (C–34), 17.9 (C–6), 17.1 (C–26), 15.8 (C–25), 13.1 (C–24) ppm; and MS (ESI): *m*/*z* (%) 555.6 ([M−H]^−^, 90).

### 3.10. (2α,3β,4α,6β) 2,3,23-Tris(acetyloxy)-6-hydroxyurs-12-en-28-oic Acid (**6**) [99598-46-6]

Following GPA from **3** (500 mg, 0.99 mmol) followed by column chromatography (SiO_2_, *n*-hexane/ethyl acetate, 8:2), **6** (392 mg, 78%) was obtained as a colorless solid; m.p.: 188–194 °C (lit.: [40] 189–192 °C); ${\left[\alpha \right]}_{D}^{20}=$ +15.0° (*c* = 0.18, CHCl_3_) [lit.: [41]$\;{\left[\alpha \right]}_{D}^{20}=$ +19.5° (CHCl_3_)]; R_F_ = 0.19 (SiO_2_, CHCl_3_/MeOH 100:1); IR (ATR): ν = 3533*br*, 3230*br*, 2921*br*, 2875*w*, 1755*s*, 1726*s*, 1707*s* 1458*w*, 1363*w*, 1230*s*, 1031*s* cm^−1^; ^1^H NMR (400 MHz, CDCl_3_): δ = 5.28 (*t*, *J* = 3.6 Hz, 1H, 12–H), 5.23 (*ddd*, *J* = 11.6, 10.3, 4.8 Hz, 1H, 2–H), 5.01 (*d*, *J* = 10.2 Hz, 1H, 3–H), 4.34 (*d*, *J* = 3.9 Hz, 1H, 6–H), 3.94 (*d*, *J* = 12.0 Hz, 1H, 23–H_a_), 3.71 (*d*, *J* = 12.0 Hz, 1H, 23–H_b_), 2.29–2.14 (*m*, 1H, 18–H), 2.06 (*s*, 3H, 36–H), 2.03 (*s*, 3H, 32–H), 1.98 (*s*, 3H, 34–H), 2.05–1.97 (*m*, 2H, 16–H_a_ + 16–H_b_), 1.95–1.78 (*m*, 2H, 11–H_a_ + 11–H_b_), 1.77–1.60 (*m*, 4H, 22–H_a_ + 22–H_b_ + 15–H_a_ + 5–H), 1.58–1.48 (*m*, 2H, 21–H_a_, 20–H), 1.46 (*d*, *J* = 2.6 Hz, 3H, 29–H), 1.37 (*d*, *J* = 1.8 Hz, 1H, 9–H), 1.35–1.28 (*m*, 5H, 21–H_b_ + 1–H_a_ + 1–H_b_ + 7–H_a_ + 7–H_b_), 1.26 (*s*, 3H, 26–H), 1.22 (*s*, 1H, 19–H), 1.08 (*s*, 1H, 15–H_b_), 1.05 (*s*, 3H, 25–H), 1.04 (*s*, 3H, 27–H), 0.95 (*d*, *J* = 6.2 Hz, 3H, 30–H), 0.86 (*s*, 3H, 24–H) ppm; ^13^C NMR (100 MHz, CDCl_3_): δ = 183.3 (C–28), 170.8 (C–35), 170.4 (C–31), 170.3 (C–33), 137.2 (C–13), 125.6 (C–12), 74.9 (C–3), 69.9 (C–2), 67.9 (C–6), 65.4 (C–23), 52.4 (C–18), 48.2 (C–5), 47.9 (C–17), 47.8 (C–9), 47.1 (C–10), 45.8 (C–4), 42.4 (C–1), 40.7 (C–20), 39.0 (C–19), 38.8 (C–8), 38.6 (C–7), 37.3 (C–14), 36.6 (C–22), 30.6 (C–21), 27.9 (C–15), 24.0 (C–16), 23.5 (C–11), 23.3 (C–27), 21.1 (C–30), 21.0 (C–34), 20.9 (C–32), 20.8 (C–36), 18.6 (C–29), 18.5 (C–25), 16.9 (C–26), 15.3 (C–24) ppm; and MS (ESI): *m*/*z* (%) 629.3 ([M−H]^−^, 90); analysis calculated for C_36_H_54_O_9_ (630.81): C 68.54, H 8.63; found: C 68.36, H 8.90.

### 3.11. (3β,4α) 3-Acetyloxy-N-(benzyl)-23-oxo-olean-12-en-28-amide (**7**)

Following GPB from **4** (300 mg, 0.58 mmol) and benzylamine (186 mg, 1.74 mmol) followed by chromatography (SiO_2_, *n*-hexane/ethyl acetate, 8:2), **7** (242 mg, 81%) was obtained as a colorless solid; m.p.: 119–121 °C; ${\left[\alpha \right]}_{D}^{20}$ = +35.1° (*c* = 0.15, CHCl_3_); R_F_ = 0.30 (SiO_2_, *n*-hexane/ethyl acetate, 8:2); IR (ATR): ν = 3391*br*, 2944*w*, 2872*w*, 1732*s*, 1645*s*, 1513*s*, 1452*s*, 1363*s*, 1233*vs*, 1028*s*, 696*w* cm^−1^; UV-Vis (CHCl_3_): λ_max_ (log ε) = 245 nm (2.37); ^1^H NMR (400 MHz, CDCl_3_): δ = 9.27 (*s*, 1H, 23–H), 7.38–7.17 (*m*, 6H, 35–H + 39–H + 37–H + 36–H + 38–H), 5.31 (*t*, *J* = 3.6 Hz, 1H, 12–H), 4.96 (*t*, *J* = 5.7 Hz, 1H, 3–H), 4.96 (*t*, *J* = 5.7 Hz, 1H, 33–H_a_), 4.59 (*dd*, *J* = 14.7, 6.2 Hz, 1H, 33–H_b_), 2.61–2.52 (*m*, 1H, 18–H), 2.03 (*s*, 1H, 32–H), 1.95 (*s*, 2H, 16–H_a_ + 16–H_b_), 1.92–1.84 (*m*, 2H, 11–H_a_ + 11–H_b_), 1.84–1.62 (*m*, 9H, 9–H + 19–H_a_ + 1–H_a_ + 7–H_a_ + 7–H_b_ + 15–H_a_ + 15–H_b_ + 2–H_a_ + 2–H_b_), 1.60–1.49 (*m*, 2H, 22–H_a_ + 6–H_a_), 1.43–1.37 (*m*, 1H, 21–H_a_), 1.36–1.31 (*m*, 1H, 5–H), 1.25 (*t*, *J* = 7.1 Hz, 2H, 22–H_b_ + 21–H_b_), 1.21–1.17 (*m*, 1H, 19–H_b_), 1.16 (*s*, 3H, 27–H), 1.12–1.00 (*m*, 2H, 15–H_b_ + 1–H_b_), 1.08 (*s*, 3H, 24–H), 0.97–0.82 (*m*, 2H, 6–H_a_ + 6–H_b_), 0.98 (*s*, 3H, 26–H), 0.94 (*s*, 3H, 29–H), 0.90 (*s*, 3H, 30–H), 0.66 (*s*, 3H, 25–H) ppm; ^13^C NMR (100 MHz, CDCl_3_): δ = 204.4 (C–23), 177.8 (C–28), 170.3 (C–31), 144.9 (C–13), 138.4 (C–34), 128.6 (C–35), 128.6 (C–39), 127.8 (C–36), 127.8 (C–38), 127.4 (C–37), 122.3 (C–12), 73.3 (C–3), 54.2 (C–4), 47.8 (C–5), 47.5 (C–9), 46.6 (C–17), 46.3 (C–19), 43.6 (C–33), 42.3 (C–14), 42.1 (C–18), 39.7 (C–80), 37.8 (C–1), 35.7 (C–10), 34.1 (C–21), 33.0 (C–29), 32.7 (C–7), 31.7 (C–22), 30.7 (C–20), 27.3 (C–15), 25.7 (C–27), 23.8 (C–30), 23.6 (C–11), 22.5 (C–2), 21.0 (C–16), 21.0 (C–32), 20.4 (C–6), 16.9 (C–25), 15.6 (C–26), 9.5 (C–24) ppm; MS (ESI): *m*/*z* (%) 602.6 ([M + H]^+^, 20); 624.6 ([M + Na]^+^, 60); and 640.6 ([M + K]^+^, 30); analysis calculated for C_39_H_55_NO_4_ (601.87): C 77.83, H 9.21, N 2.33; found: C 77.59, H 9.43, N 2.08.

### 3.12. (3β,4α) 3-Acetyloxy-N-(phenyl)-23-oxo-olean-12-en-28-amide (**8**)

Following GPB from **4** (300 mg, 0.58 mmol) and aniline (162 mg, 1.74 mmol) followed by chromatography (SiO_2_, *n*-hexane/ethyl acetate, 9:1), **8** (194 mg, 65%) was obtained as a colorless solid; m.p.: 150–152 °C; ${\left[\alpha \right]}_{D}^{20}$ = +85.3° (*c* = 0.17, CHCl_3_); R_F_ = 0.20 (SiO_2_, *n*-hexane/ethyl acetate, 9:1); IR (ATR): ν = 3385*br*, 2941*br*, 2857*w*, 1735*s*, 1596*s*, 1239*vs*, 1025*s*, 751*vs*, 690*vs* cm^−1^; UV-Vis (CHCl_3_): λ_max_ (log ε) = 243 nm (0.4); ^1^H NMR (500 MHz, CDCl_3_): δ = 9.28 (*s*, 1H, 23–H), 7.50–7.44 (*m*, 2H, 34–H + 38–H), 7.35–7.29 (*m*, 2H, 35–H + 37–H), 7.17–7.15 (*m*, 1H, 36–H), 5.56 (*q*, *J* = 2.8, 1.9 Hz, 1H, 12–H), 5.03 (*d*, *J* = 4.6 Hz, 1H, 3–H), 2.73–2.65 (*m*, 1H, 18–H), 2.14–2.01 (*m*, 3H, 32–H), 1.98 (*s*, 2H, 16–H_a_ + 16–H_b_), 1.88–1.77 (*m*, 3H, 11–H_a_ + 11–H_b_ + 7–H_a_), 1.76–1.54 (*m*, 7H, 9–H + 19–H_a_ + 1–H_a_ + 7–H_b_ + 15–H_a_ + 2–H_a_ + 2–H_b_), 1.53–1.46 (*m*, 2H, 22–H_a_ + 6–H_a_), 1.42–1.32 (*m*, 1H, 21–H_a_), 1.30–1.24 (*m*, 1H, 5–H), 1.23–1.21 (*m*, 2H, 22–H_b_ + 21–H_b_ + 6–H_b_), 1.23 (*s*, 1H, 19–H_b_), 1.20 (*s*, 3H, 27–H), 1.10–1.04 (*m*, 2H, 15–H_b_ + 1–H_b_), 1.07 (*s*, 3H, 24–H) 1.02 (*s*, 3H, 26–H), 0.94 (*s*, 3H, 29–H), 0.92 (*s*, 3H, 30–H), 0.73 (*s*, 3H, 25–H) ppm; ^13^C NMR (125 MHz, CDCl_3_): δ = 177.0 (C–28), 171.3 (C–33), 170.9 (C–31), 138.6 (C–13), 128.4 (C–12), 80.9 (C–3), 73.6 (C–19), 70.3 (C–2), 55.5 (C–18), 55.1 (C–17), 54.9 (C–5), 47.2 (C–9), 45.6 (C–35), 45.4 (C–36), 43.9 (C–1), 41.7 (C–14), 41.0 (C–20), 39.8 (C–8), 39.4 (C–4), 38.1 (C–10), 34.8 (C–22), 32.8 (C–7), 28.4 (C–23), 28.3 (C–15), 27.7 (C–29), 25.9 (C–21), 25.3 (C–16), 24.3 (C–27), 23.7 (C–11), 21.1 (C–34), 20.9 (C–32), 18.3 (C–6), 17.6 (C–24), 16.6 (C–26), 16.4 (C–25), 16.0 (C–30) ppm; and MS (ESI): *m*/*z* (%) 586.7 ([M-H]^−^, 90); analysis calculated for C_38_H_53_NO_4_ (587.85): C 77.64, H 9.09, N 2.38; found: C 77.41, H 9.23, N 2.05.

### 3.13. (3β,4α) 3-Acetyloxy-N-(piperazinyl)-23-oxo-olean-12-en-28-amide (**9**)

Following GPB from **4** (300 mg, 0.58 mmol) and piperazine (150 mg, 1.74 mmol) followed by chromatography (SiO_2_, chloroform/methanol, 9:1), **9** (243 mg, 81%) was obtained as a colorless solid; m.p.: 150–154 °C; ${\left[\alpha \right]}_{D}^{20}$ = +36.5° (*c* = 1.15, CHCl_3_); R_F_ = 0.27 (SiO_2_, CHCl_3_/MeOH, 95:5); IR (ATR): ν = 3325*br*, 2947*br*, 2855*w*, 1735*s*, 1625*s*, 1239*vs*, 754s cm^−1^; ^1^H NMR (400 MHz, CDCl_3_): δ = 9.26 (*s*, 1H, 23–H), 5.21 (*t*, *J* = 3.7 Hz, 1H, 12–H), 4.98 (*dd*, *J* = 11.0, 5.3 Hz, 1H, 3–H), 3.71–3.56 (*m*, 4H, 34–H_a_ + 34–H_b_ + 35–H_a_ + 35–H_b_), 3.31–3.29 (*m*, 4H, 33–H_a_ + 33–H_b_ + 36–H_a_ + 36–H_b_), 3.08–2.83 (*m*, 1H, 18–H), 2.15–2.13 (*m*, 4H, 11–H_a_ + 11–H_b_ + 16–H_a_ + 16–H_b_), 1.93 (*s*, 3H, 32–H), 1.82–1.54 (*m*, 8H, 9–H + 19–H_a_ + 1–H_a_ + 7–H_a_ + 7–H_b_ + 15–H_a_ + 2–H_a_ + 2–H_b_), 1.50–1.40 (*m*, 3H, 22–H_a_ + 6–H_a_ + 21–H_a_), 1.29–1.25 (*m*, 1H, 5–H), 1.25–1.20 (*m*, 2H, 22–H_b_ + 21–H_b_), 1.20 (*s*, 3H, 27–H), 1.18–1.16 (*m*, 1H, 19–H_b_), 1.06 (*s*, 3H, 24–H), 1.08–1.0 (*m*, 2H, 15–H_b_ + 1–H_b_), 1.02 (*s*, 3H, 25–H), 0.98–0.84 (*m*, 1H, 6–H_b_), 0.94 (*s*, 3H, 30–H), 0.91 (*s*, 3H, 29–H), 0.77 (*s*, 3H, 26–H) ppm; ^13^C NMR (100 MHz, CDCl_3_): δ = 204.9 (C–23), 175.8 (C–28), 170.5 (C–31), 144.6 (C–13), 121.2 (C–12), 73.4 (C–3), 54.0 (C–4), 48.2 (C–33), 48.2 (C–36), 48.0 (C–17), 47.8 (C–5), 47.6 (C–9), 46.9 (C–19), 45.0 (C-34), 45.0 (C–35), 43.7 (C–18), 41.7 (C–14), 39.4 (C–8), 37.4 (C–1), 35.6 (C–10), 33.5 (C–21), 33.0 (C–7), 32.1 (C–29), 31.9 (C–22), 29.8 (C–20), 27.7 (C–15), 25.1 (C–27), 23.0 (C–30), 22.9 (C–11), 22.6 (C–16), 22.1 (C–2), 20.2 (C–32), 19.4 (C–6), 16.2 (C–26), 14.6 (C–25), 8.5 (C–24) ppm; and MS (ESI): *m*/*z* (%) 581.6 ([M + H]^+^, 90); analysis calculated for C_36_H_56_N_2_O_4_ (580.85): C 74.44, H 9.72, N 4.82; found: C 74.06, H 9.98, N 4.61.

### 3.14. (3β,4α) 3-Acetyloxy-N-(homopiperazinyl)-23-oxo-olean-12-en-28-amide (**10**)

Following GPB from **4** (300 mg, 0.58 mmol) and homopiperazine (174 mg, 1.74 mmol) followed by chromatography (SiO_2_, chloroform/methanol, 9:1), **10** (286 mg, 83%) was obtained as a colorless solid; m.p.: 176–179 °C; ${\left[\alpha \right]}_{D}^{20}$ = −4.39° (*c* = 0.145 CHCl_3_); R_F_ = 0.34 (SiO_2_, CHCl_3_/MeOH, 9:1); IR (ATR): ν = 2923*w*, 1741*s*, 1621*w*, 1455*w*, 1368*m*, 1230*vs*, 1156*w*, 1087*w*, 1043*m*, 1029*m*, 964*w*, 920*w*, 751*m*, 665*w*, 460*w*, 422*w* cm^−1^; ^1^H NMR (400 MHz, CDCl_3_): δ = 9.26 (*s*, 1H, 23-H), 5.27–5.22 (*m*, 1H, 12–H), 5.00–4.91 (*m*, 1H, 3–H), 3.97–3.37 (*m*, 6H, 33–H + 35–H + 37–H), 3.36–2.90 (*m*, 3H, 34–H + 18–H), 2.34–2.00 (*m*, 3H, 36–H + 16–H), 1.98–1.81 (*m*, 2H, 11–H), 1.94 (*s*, 3H, 32–H), 1.79–1.58 (*m*, 8H, 2–H + 1–H_a_ + 16–H_b_ + 19–H_a_ + 22–H + 9–H), 1.57–1.39 (*m*, 3H, 15–H_a_ + 6–H_a_ + 7–H_a_), 1.39–1.26 (*m*, 2H, 21–H_a_ + 5–H), 1.26–0.99 (*m*, 5H, 21–H_b_ + 7–H_b_ + 19–H_b_ + 1–H_b_ + 15–H_b_), 1.14 (*s*, 3H, 27–H), 1.06 (*s*, 3H, 24–H), 0.99–0.88 (*m*, 1H, 6–H_b_), 0.96 (*s*, 3H, 26–H), 0.93 (*s*, 3H, 30–H), 0.89 (*s*, 3H, 29–H), 0.73 (*s*, 3H, 25–H) ppm; ^13^C NMR (100 MHz, CDCl_3_): δ = 204.5 (C–23), 176.2 (C–28), 170.3 (C–31), 144.7 (C–13), 121.3 (C–12), 73.4 (C–3), 54.3 (C–4), 49.2 (C–35), 47.9 (C–5), 47.8 (C–17), 47.7 (C–9), 47.2 (C–34), 46.5 (C–33 + C–37), 46.3 (C–19), 43.6 (C–18), 42.0 (C–14), 39.4 (C–8), 37.7 (C–1), 35.9 (C–10), 33.9 (C–21), 33.0 (C–29), 32.1 (C–7), 30.4 (C–20), 30.0 (C–22), 27.8 (C–15), 26.6 (C–36), 25.9 (C–27), 24.0 (C–30), 23.3 (C–11), 22.5 (C–16), 22.5 (C–2), 21.0 (C–32), 20.4 (C–6), 16.9 (C–25), 15.6 (C–26), 9.5 (C–24) ppm; and MS (ESI): *m*/*z* (%) = 595.1 ([M + H]^+^, 100); analysis calculated for C_37_H_58_N_2_O_4_ (594.87): C 74.71, H 9.82, N 4.71; found: C 74.50, H 10.02, N 4.55.

### 3.15. (3β,4α) 3,23-Bis(acetyloxy)-N-(benzyl)-olean-12-en-28-amide (**11**)

Following GPB from **5** (450 mg, 0.81 mmol) and benzylamine (260 mg, 2.43 mmol) followed by chromatography (SiO_2_, *n*-hexane/ethyl acetate, 8:2), **11** (117 mg, 26%) was obtained as a colorless solid; m.p.: 210–214 °C; ${\left[\alpha \right]}_{D}^{20}$ = +39.22° (*c* = 0.159, CHCl_3_); R_F_ = 0.20 (SiO_2_, *n*-hexane/ethyl acetate, 8:2); IR (ATR): ν = 3432*w*, 2950*br*, 1726*vs*, 1631*s*, 1253*vs*, 1034*s*, 745*vs* cm^−1^; UV-Vis (CHCl_3_): λ_max_ (log ε) = 260 nm (3.28); ^1^H NMR (400 MHz, CDCl_3_): δ = 7.35–7.27 (*m*, 2H, 37–H + 41–H), 7.25–7.19 (*m*, 3H, 38–H + 40–H + 39–H), 5.30 (*t*, *J* = 3.6 Hz, 1H, 12–H), 4.76 (*dd*, *J* = 11.3, 4.9 Hz, 1H, 3–H), 4.63–4.57 (*m*, 1H, 35–H_a_), 4.14 (*dd*, *J* = 14.7, 4.4 Hz, 1H, 35–H_b_), 3.86 (*d*, *J* = 11.6 Hz, 1H, 23–H_a_), 3.70 (*d*, *J* = 11.7 Hz, 1H, 23–H_b_), 2.60–2.50 (*m*, 1H, 18–H), 2.05 (*s*, 3H, 15–H_a_ + 16–H_a_ + 22–H_a_), 2.05 (*s*, 3H, 34–H), 2.01 (*s*, 3H, 32–H), 1.99–1.91 (*m*, 5H, 11–H_a_ + 11–H_b_ + 16–H_b_), 1.87–1.77 (*m*, 1H, 22–H_b_), 1.76–1.54 (*m*, 5H, 7–H_a_ + 7–H_b_ + 19–H_a_ + 9–H + 1–H_a_), 1.52 (*d*, *J* = 4.2 Hz, 1H, 2–H_a_), 1.45–1.31 (*m*, 3H, 6–H_a_ + 6–H_b_ + 21–H_a_), 1.28–1.15 (*m*, 4H, 19–H_b_ + 21–H_b_ + 15–H_b_ + 5–H), 1.14–0.95 (*m*, 2H, 1–H_b_ + 2–H_b_) 1.13 (*s*, 3H, 27–H), 0.93 (*s*, 3H, 25–H), 0.91 (*s*, 3H, 29–H), 0.90 (*s*, 3H, 30–H), 0.83 (*s*, 3H, 24–H), 0.66 (*s*, 3H, 26–H) ppm; ^13^C NMR (100 MHz, CDCl_3_): δ = 177.9 (C–28), 170.9 (C–31), 170.6 (C–33), 144.9 (C–13), 138.4 (C–36), 128.6 (C–37), 128.6 (C–41), 127.8 (C-38), 127.8 (C–40), 127.3 (C–39), 122.7 (C–12), 74.4 (C–3), 65.4 (C–23), 47.8 (C–5), 47.6 (C–9), 46.6 (C–17), 46.3 (C–19), 43.6 (C–35), 42.3 (C–4), 42.0 (C–14), 40.5 (C–18), 39.3 (C–8), 37.8 (C–1), 36.6 (C–10), 34.1 (C–21), 33.0 (C–30), 32.6 (C–7), 32.0 (C–20), 30.7 (C–15), 27.3 (C–2), 25.6 (C–27), 23.8 (C–29), 23.6 (C–11), 23.4 (C–16), 22.9 (C–22), 21.2 (C–32), 20.9 (C–34), 17.9 (C–6), 16.9 (C–26), 15.8 (C–25), 13.1 (C–24) ppm; and MS (ESI): *m*/*z* (%) 646.8 ([M + H]^+^, (23)), 668.7 ([M + Na]^+^, 55), 684.6 ([M + K]^+^, 60); analysis calculated for C_41_H_59_NO_5_ (645.93): C 76.24, H 9.21, N 2.17; found: C 75.98, H 9.44, N 2.02.

### 3.16. (3β,4α) 3,23-Bis(acetyloxy)-N-(phenyl)-olean-12-en-28-amide (**12**)

Following GPB from **5** (450 mg, 0.81 mmol) and aniline (226 mg, 2.43 mmol) followed by chromatography (SiO_2_, *n*-hexane/ethyl acetate, 8:2), **12** (440 mg, 98%) was obtained as a colorless solid; m.p.: 210–214 °C; ${\left[\alpha \right]}_{D}^{20}$ = +39.2° (*c* = 0.16, CHCl_3_); R_F_ = 0.20 (SiO_2_, *n*-hexane/ethyl acetate, 8:2); IR (ATR): ν = 2947*br*, 1744*s*, 1657*s*, 1267*s*, 1028*w*, 748*s* cm^−1^; UV-Vis (CHCl_3_): λ_max_ (log ε) = 237 nm (0.4); ^1^H NMR (400 MHz, CDCl_3_): δ = 7.49–7.46 (*m*, 2H, 36–H + 40–H), 7.35–7.24 (*m*, 2H, 37–H + 39–H), 7.10–7.05 (*m*, 1H, 38–H), 5.55 (*t*, *J* = 3.6 Hz, 1H, 12–H), 4.78 (*dd*, *J* = 11.5, 4.9 Hz, 1H, 3–H), 3.85 (*d*, *J* = 11.6 Hz, 1H, 23–H_a_), 3.70 (*d*, *J* = 11.6 Hz, 1H, 23–H_b_), 2.72–2.64 (*m*, 1H, 18–H), 2.06 (*s*, 3H, 15–H_a_ + 16–H_a_ + 22–H_a_), 2.05 (*s*, 3H, 34–H), 2.02 (*s*, 3H, 32–H), 1.98 (*d*, *J* = 3.6 Hz, 2H, 11–H_a_ + 11–H_b_), 1.88–1.69 (*m*, 5H, 16–H_b_ + 2–H_a_ + 2–H_b_ + 22–H_b_ + 7–H_a_), 1.64–1.59 (*m*, 5H, 7–H_b_ + 19–H_a_ + 9–H + 1–H_a_ + 5–H), 1.47–1.33 (*m*, 3H, 6–H_a_ + 6–H_b_ + 21–H_a_), 1.31–1.22 (*m*, 3H, 19–H_b_ + 21–H_b_ + 15–H_b_), 1.19 (*s*, 3H, 27–H), 1.10 (*tq*, *J* = 10.8, 3.5 Hz, 1H, 1–H_b_), 0.94 (*s*, 3H, 29–H), 0.94 (*s*, 3H, 30–H), 0.94 (*s*, 3H, 25–H), 0.81 (*s*, 3H, 24–H), 0.72 (*s*, 3H, 26–H) ppm; ^13^C NMR (100 MHz, CDCl_3_): δ = 176.4 (C–28), 170.9 (C–31), 170.7 (C–33), 145.1 (C–13), 138.0 (C–35), 128.8 (C–37), 128.8 (C–39), 124.1 (C–38), 123.1 (C–12), 119.8 (C–36), 119.8 (C–40), 74.4 (C–3), 65.4 (C–23), 47.8 (C–5), 47.6 (C–9), 47.2 (C–17), 46.7 (C–19), 42.6 (C–4), 42.1 (C–14), 40.5 (C–18), 39.4 (C–8), 37.8 (C–1), 36.7 (C–10), 34.2 (C–21), 33.0 (C–30), 32.5 (C–7), 32.0 (C–20), 30.7 (C–15), 27.3 (C–2), 25.6 (C–27), 24.1 (C–29), 23.6 (C–11), 23.6 (C–16), 22.9 (C–22), 21.2 (C–32), 20.9 (C–34), 17.8 (C–6), 16.9 (C–26), 15.9 (C–25), 13.1 (C–24) ppm; and MS (ESI): *m*/*z* (%) 632.7 ([M + H]^+^, 40), 654.7 ([M + Na]^+^, 35), 670.7 ([M + K]^+^, 25); analysis calculated for C_40_H_57_NO_5_ (631.90): C 76.03, H 9.09, N 2.22; found: C 75.81, H 9.27, N 1.97.

### 3.17. (3β,4α)3,23-Bis(acetyloxy)-N-(piperazinyl)-olean-12-en-28-amide (**13**)

Following GPB from **5** (450 mg, 0.81 mmol) and piperazine (210 mg, 2.43 mmol) followed by chromatography (SiO_2_, *n*-hexane/ethyl acetate, 8:2), **13** (360 mg, 80%) was obtained as a colorless solid; m.p.: 183–187 °C; ${\left[\alpha \right]}_{D}^{20}=$ +37.9° (*c* = 0.18, CHCl_3_); R_F_ = 0.20 (SiO_2_, *n*-hexane/ethyl acetate, 8:2); IR (ATR): ᴠ = 3354*br*, 2947*br*, 1735*s*, 1625*s*, 1410*w*, 1242*vs*, 1002*s*, 754*w* cm^−1^; ^1^H NMR (500 MHz, CDCl_3_): δ = 5.25 (*t*, *J* = 3.3 Hz, 1H, 12–H), 4.78 (*dd*, *J* = 11.5, 5.0 Hz, 1H, 3–H), 3.87 (*dd*, *J* = 11.6, 2.1 Hz, 1H, 23–H_a_), 3.71–3.51 (*m*, 5H, 23–H_b_ + 35–H_a_ + 35–H_b_ + 38–H_a_ + 38–H_b_), 3.37–2.96 (*m*, 4H, 36–H_a_ + 36–H_b_ + 37–H_a_ + 37–H_b_), 2.96–2.75 (*m*, 1H, 18–H), 2.44 (*dd*, *J* = 17.5, 9.9 Hz, 1H, 5–H), 2.22–2.08 (*m*, 1H, 22–H_a_), 2.08–2.05 (*m*, 5H, 15–H_a_ + 16–H_a_), 2.05 (*s*, 3H, 34–H), 2.01 (*s*, 3H, 32–H), 1.88 (*td*, *J* = 17.7, 16.0, 11.8 Hz, 5H, 11–H_a_ + 11–H_b_ + 16–H_b_ + 2–H_a_ + 2–H_b_), 1.76–1.54 (*m*, 6H, 22–H_b_ + 7–H_a_ + 7–H_b_ + 19–H_a_ + 9–H + 1–H_a_), 1.41–1.27 (*m*, 3H, 6–H_a_ + 6–H_b_ + 21–H_a_), 1.26–1.13 (*m*, 3H, 19–H_b_ + 21–H_b_ + 15–H_b_), 1.11 (*s*, 3H, 27–H), 1.04 (*dd*, *J* = 13.7, 4.0 Hz, 1H, 1–H_b_), 0.95 (*s*, 3H, 25–H), 0.91 (*s*, 3H, 29–H), 0.89 (*s*, 3H, 30–H), 0.82 (*s*, 3H, 24–H), 0.73 (*s*, 3H, 26–H) ppm; ^13^C NMR (125 MHz, CDCl_3_): δ = 174.9 (C–28) 171.0 (C–31), 170.6 (C–33), 144.9 (C–13), 121.3 (C–12), 74.6 (C–3), 65.5 (C–23), 51.6 (C–5), 47.9 (C–35), 47.9 (C–38), 47.9 (C-9), 47.3 (C–17), 46.4 (C–19), 46.2 (C–36), 46.2 (C–37), 45.0 (C–4), 41.8 (C–14), 40.5 (C–18), 39.1 (C–8), 37.7 (C–1), 36.8 (C–10), 34.0 (C–21), 33.0 (C–30), 32.5 (C–7), 32.2 (C–20), 30.4 (C–15), 29.9 (C–2), 27.9 (C–27), 25.8 (C–29), 24.1 (C–11), 23.4 (C–16), 22.9 (C–22), 21.2 (C–32), 20.9 (C–34), 17.9 (C–6), 16.9 (C–26), 15.8 (C–25), 13.1 (C–24) ppm; and MS (ESI): *m*/*z* (%) 626.0 ([M + H]^+^, 80); analysis calculated for C_38_H_60_N_2_O_5_ (624.91): C 73.04, H 9.68, N 4.48; found: C 72.84, H 9.82, N 4.15.

### 3.18. (3β,4α)3,23-Bis(acetyloxy)-N-(homopiperazinyl)-olean-12-en-28-amide (**14**)

Following GPB from **5** (450 mg, 0.81 mmol) and homopiperazine (243 mg, 2.43 mmol) followed by chromatography (SiO_2_, chloroform/methanol, 9:1), **14** (186 mg, 41%) was obtained as a colorless solid; m.p.: 176–180 °C; ${\left[\alpha \right]}_{D}^{20}$ = +31.2° (*c* = 0.11, CHCl_3_); R_F_ = 0.36 (SiO_2_, CHCl_3_/MeOH 9:1); IR (ATR): ᴠ = 2944*br*, 1738*s*, 1622*w*, 1467*w*, 1239*s*,1034*s*, 745*w* cm^−1^; ^1^H NMR (400 MHz, CDCl_3_): δ = 5.27–5.22 (*m*, 1H, 12–H), 4.77 (*dd*, *J* = 11.5, 5.0 Hz, 1H, 3–H), 4.02–3.82 (*m*, 1H, 23–H_a_), 3.69 (*d*, *J* = 11.7 Hz, 1H, 23–H_b_), 3.44–3.14 (*m*, 2H, 39–H_a_ + 39–H_b_), 3.06 (*d*, *J* = 13.4 Hz, 2H, 35–H_a_ + 35–H_b_), 2.83- 2.50 (*m*, 5H, 36–H_a_ + 36–H_b_ + 37–H_a_ + 37–H_b_ + 18–H), 2.18–2.08 (*m*, 2H, 16–H_a_ + 22–H_a_), 2.05 (*s*, 3H, 34–H), 2.01 (*s*, 3H, 32–H), 2.00–1.79 (*m*, 2H, 11–H_a_ + 11–H_b_), 1.77–1.44 (*m*, 10H, 16–H_b_ + 15–H_a_ + 38–H_a_ + 38– H_b_ + 22–H_b_ + 2–H_a_ + 2–H_b_ + 19–H_a_ + 1–H_a_ + 5–H), 1.40–1.33 (*m*, 5H, 7–H_a_ + 19–H_b_ + 6–H_a_ + 6–H_b_ + 21–H_a_), 1.26–1.14 (*m*, 5H, 7–H_b_ + 9–H + 1–H_b_ + 21–H_b_ + 15–H_b_), 1.11 (*s*, 3H, 27–H), 0.94 (*s*, 3H, 25–H), 0.93 (*s*, 3H, 29–H), 0.89 (*s*, 3H, 30–H), 0.82 (*s*, 3H, 24–H), 0.71 (*s*, 3H, 26–H) ppm; ^13^C NMR (100 MHz, CDCl_3_): δ = 176.4 (C–28), 170.9 (C–31), 170.6 (C–33), 144.5 (C–13), 121.7 (C–12), 74.5 (C–3), 65.5 (C–23), 56.6 (C–39), 47.8 (C–5), 47.8 (C–9), 47.0 (C–36), 46.6 (C–17), 45.7 (C–19), 46.3 (C–37), 43.7 (C–35), 41.9 (C–4), 40.9 (C–14), 40.5 (C–18), 39.1 (C–8), 37.7 (C–1), 36.8 (C–10), 33.8 (C–21), 33.0 (C–30), 32.4 (C–7), 31.9 (C–20), 30.0 (C–15), 27.8 (C–2), 25.9 (C–38), 25.5 (C–27), 24.0 (C–29), 23.3 (C–11), 22.9 (C–16), 21.2 (C–32), 20.9 (C–34), 22.9 (C–22), 17.9 (C–6), 16.9 (C–26), 15.8 (C–25), 13.1 (C–24) ppm; and MS (ESI): *m*/*z* (%) 639.7 ([M + H]^+^, 90); analysis calculated for C_39_H_62_N_2_O_5_ (638.93): C 73.31, H 9.78, N 4.38; found: C 73.05, H 9.93, N 4.11.

### 3.19. (2α,3β,4α,6β) 2,3,23-Tris(acetyloxy)-N-(benzyl)-6-hydroxyurs-12-en-28-amide (**15**)

Following GPB from **6** (150 mg, 0.24 mmol) and benzylamine (77 mg, 0.72 mmol) followed by chromatography (SiO_2_, *n*-hexane/ethyl acetate, 8:2), **15** (186 mg, 41%) was obtained as a colorless solid; m.p.: 90–95 °C; ${\left[\alpha \right]}_{D}^{20}$ = −11.7° (*c* = 0.12, CHCl_3_); R_F_ = 0.25 (SiO_2_, *n*-hexane/ethyl acetate, 8:2); IR (ATR): ν = 3388*br*, 2924*br*, 1741*vs*, 1643*s*, 1371*s*, 1233*vs*, 1028*vs*, 601*w* cm^−1^; UV-Vis (CHCl_3_): λ_max_ (log ε) = 260 nm (0.1); ^1^H NMR (400 MHz, CDCl_3_): δ = 7.32 (*dd*, *J* = 8.0, 6.3 Hz, 2H, 40–H + 42–H), 7.30–7.22 (*m*, 3H, 39–H + 43–H + 41–H), 5.33–5.26 (*m*, 2H, 12–H + 2–H), 5.13–4.99 (*m*, 1H, 3–H), 4.50 (*d*, *J* = 5.7 Hz, 1H, 6–H), 4.47 (*d*, *J* = 5.6 Hz, 2H, 1–H_a_ + 37–H_a_), 4.26–4.19 (*m*, 3H, 23–H_a_ + 1–H_b_ + 37–H_b_), 3.69 (*d*, *J* = 12.0 Hz, 1H, 23–H_b_), 2.34 (*dd*, *J* = 19.0, 6.4 Hz, 1H, 18–H), 2.05 (*s*, 3H, 36–H), 2.00 (*s*, 3H, 32–H), 2.00 (*s*, 1H, 16–H_a_), 1.98 (*s*, 3H, 34–H), 1.95–1.80 (*m*, 3H, 11–H_a_ + 11–H_b_ + 16–H_b_), 1.70–1.56 (*m*, 4H, 22–H_a_ + 22–H_b_ + 15–H_a_ + 5–H), 1.56–1.44 (*m*, 2H, 21–H_a_ + 20–H), 1.40–1.28 (*m*, 4H, 9–H + 19–H + 21–H_b_ + 7–H_a_), 1.25 (*s*, 3H, 24–H), 1.20 (*s*, 3H, 26–H), 1.18–1.15 (*m*, 1H, 15–H_b_), 0.95 (*d*, *J* = 5.9 Hz, 1H, 7–H_b_), 0.95 (*s*, 3H, 29–H), 0.88 (*s*, 3H, 25–H), 0.84 (*s*, 3H, 27–H), 0.83 (*s*, 3H, 24–H), 0.82 (*d*, *J* = 5.7 Hz, 3H, 30–H) ppm; ^13^C NMR (100 MHz, CDCl_3_): δ = 177.6 (C–28), 171.0 (C–35), 170.4 (C–31), 170.2 (C–33), 141.0 (C–38), 138.3 (C–13), 128.7 (C–39), 128.7 (C–43), 128.0 (C–40), 128.0 (C–42), 127.5 (C–41), 126.2 (C–12), 73.4 (C–3), 69.1 (C–2), 67.9 (C–6), 64.9 (C–23), 54.4 (C–18), 48.1 (C–5), 47.2 (C–10), 46.0 (C–17), 45.0 (C–4), 43.9 (C–1), 43.8 (C–37), 42.3 (C–9), 39.0 (C–19), 38.9 (C–8), 38.4 (C–7), 38.2 (C–14), 37.0 (C–20), 32.3 (C–22), 30.8 (C–21), 27.1 (C–15), 24.7 (C–16), 23.4 (C–11), 22.3 (C–26), 21.9 (C–34), 21.2 (C–30), 21.1 (C–25), 21.0 (C–27), 20.9 (C–32), 20.8 (C–36), 17.3 (C–29), 14.1 (C–24) ppm; and MS (ESI): *m*/*z* (%) 702.3 ([M + H−H_2_O]^−^, 90), 724.2 ([M + H− H_2_O + Na]^+^, 85); analysis calculated for C_43_H_61_NO_8_ (719.96): C 71.74, H 8.54, N 1.95; found: C 71.55, H 8.71, N 1.68.

### 3.20. (2α,3β,4α,6β) 2,3,23-Tris(acetyloxy)-N-(phenyl)-6-hydroxyurs-12-en-28-amide (**16**)

Following GPB from **6** (150 mg, 0.24 mmol) and aniline (67 mg, 0.72 mmol) followed by chromatography (SiO_2_, *n*-hexane/ethyl acetate, 8:2), **16** (170 mg, 60%) was obtained as a colorless solid; m.p.: 135–139 °C; ${\left[\alpha \right]}_{D}^{20}$ = +10.9° (*c* = 0.08, CHCl_3_); R_F_ = 0.15 (SiO_2_, *n*-hexane/ethyl acetate, 8:2); IR (ATR): ν = 3403*br*, 2927*br*, 1741*s*, 1438*s*, 1233*vs*, 1031*s*, 757*s*, 690*s* cm^−1^; UV-Vis (CHCl_3_): λ_max_ (log ε) = 246 nm (0.3); ^1^H NMR (500 MHz, CDCl_3_): δ = 7.50–7.42 (*m*, 2H, 38–H + 42–H), 7.33–7.24 (*m*, 2H, 39–H + 41–H), 7.10–7.05 (*m*, 1H, 40–H), 5.62 (*d*, *J* = 3.7 Hz, 1H, 12–H), 5.33–5.25 (*m*, 1H, 2–H), 5.15 (*d*, *J* = 10.4 Hz, 1H, 3–H), 4.34 (*d*, J = 3.9 Hz, 1H, 6–H), 4.23 (*d*, *J* = 11.9 Hz, 1H, 23–H_a_), 3.70 (*d*, *J* = 12.0 Hz, 1H, 23–H_b_), 2.38 (*s*, 1H, 21–H_a_), 2.36 (*dd*, *J* = 18.9, 6.4 Hz, 1H, 18–H), 2.07 (*s*, 3H, 36–H), 2.06 (*s*, 3H, 32–H), 2.02 (*s*, 3H, 34–H), 2.00–1.98 (*m*, 3H, 16–H_a_ + 16–H_b_ + 7–H_a_), 1.91–1.79 (*m*, 4H, 11–H_a_ + 11–H_b_ + 5–H + 9–H), 1.72 (*td*, *J* = 13.8, 4.3 Hz, 1H, 15–H_a_), 1.66–1.53 (*m*, 3H, 22–H_a_ + 22–H_b_ + 7–H_b_), 1.50–1.42 (*m*, 2H, 21–H_b_ + 19–H), 1.42–1.30 (*m*, 3H, 1–H_a_ + 1–H_b_ + 20–H), 1.26–1.22 (*m*, 1H, 15–H_b_), 1.21 (*s*, 3H, 26–H), 1.12 (*s*, 3H, 27–H), 1.02 (*s*, 3H, 25–H), 0.99 (*d*, *J* = 2.0 Hz, 3H, 29–H), 0.92 (*d*, *J* = 6.4 Hz, 3H, 30–H), 0.87 (*s*, 3H, 24–H) ppm; ^13^C NMR (125 MHz, CDCl_3_): δ = 176.1 (C–28), 170.9 (C–35), 170.4 (C–31), 170.3 (C–33), 141.4 (C–13), 138.1 (C–37), 129.1 (C–39) 129.1 (C–41), 126.6 (C-12), 124.1 (C-40), 119.7 (C-38), 119.7 (C–42), 73.5 (C–3), 69.1 (C–2), 67.6 (C–6), 64.9 (C–23), 54.7 (C–18), 48.0 (C–5), 47.8 (C–17), 46.1 (C–9), 45.0 (C–4), 44.1 (C–10), 42.4 (C–1), 39.1 (C–19), 38.7 (C–8), 38.5 (C–7), 38.2 (C–20), 37.3 (C–14), 36.8 (C–22), 32.3 (C–21), 27.1 (C–15), 24.9 (C–16), 23.7 (C–11), 22.4 (C–27), 21.9 (C–30), 21.1 (C–34), 20.9 (C–32), 20.8 (C–36), 18.6 (C–29), 17.4 (C–25), 17.2 (C–26), 15.3 (C–24) ppm; and MS (ESI): *m*/*z* (%) 686.1 ([M-H-H_2_O]^−^, 90), 704.2 ([M-H]^−^, 17); analysis calculated for C_42_H_59_NO_8_ (705.93): C 71.46, H 8.42, N 1.98; found: C 71.21, H 8.57, N 1.62.

### 3.21. (2α,3β,4α,6β) 2,3,23-Tris(acetyloxy)-N-(piperazinyl)-6-hydroxyurs-12-en-28-amide (**17**)

Following GPB from **6** (500 mg, 0.79 mmol) and piperazine (204 mg, 2.37 mmol) followed by chromatography (SiO_2_, chloroform/methanol, 9:1), **17** (206 mg, 41%) was obtained as a colorless solid; m.p.: 133–138 °C;$\;{\left[\alpha \right]}_{D}^{20}$ = +20.25° (*c* = 0.08, CHCl_3_); R_F_ = 0.66 (SiO_2_, CHCl_3_/MeOH, 9:1); IR (ATR): ν = 3414*br*, 2932*br*, 1741*s*, 1631*s*, 1366*s*, 1233*vs*, 1025*s*, 748*s* cm^−1^; ^1^H NMR (500 MHz, CDCl_3_): δ = 5.36–5.23 (*m*, 1H, 12–H), 5.13 (*d*, *J* = 10.4 Hz, 2H, 3–H + 2–H), 4.24 (*d*, *J* = 11.9 Hz, 2H, 6–H + 23–H_a_), 3.73–3.57 (*m*, 1H, 23–H_b_), 3.42–3.24 (*m*, 4H, 37–H_a_ + 37–H_b_ + 40–H_a_ + 40–H_b_), 2.89–2.46 (*m*, 4H, 38–H_a_ + 38–H_b_ +39–H_a_ + 39–H_b_), 2.44 (*d*, *J* = 11.2 Hz, 1H, 18–H), 2.33 (*dd*, *J* = 18.8, 6.2 Hz, 1H, 22–H_a_), 2.03 (*s*, 3H, 36–H), 1.99 (*s*, 3H, 32–H), 1.98 (*s*, 3H, 1–H_a_, 16–H_a_ + 16–H_b_), 1.98 (*s*, 3H, 34–H), 1.93–1.79 (*m*, 2H, 11–H_a_ + 11–H_b_), 1.75 (*dd*, *J* = 11.7, 5.3 Hz, 1H, 9–H), 1.68–1.58 (*m*, 2H, 22–H_b_ + 5–H), 1.52–1.46 (*m*, 1H, 21–H_a_), 1.36–1.26 (*m*, 5H, 1–H_b_ + 21–H_b_ + 20–H + 19–H + 7–H_a_), 1.21 (*s*, 3H, 26–H), 1.16 (*d*, *J* = 3.6 Hz, 1H, 15–H_a_), 1.12 (*s*, 3H, 29–H), 1.09–0.98 (*m*, 1H, 7–H_b_), 0.95 (*s*, 3H, 25–H), 0.96–0.92 (*m*, 1H, 15–H_b_), 0.94 (*s*, 3H, 27–H), 0.88 (*s*, 3H, 30–H), 0.85 (*s*, 3H, 24–H) ppm; ^13^C NMR (125 MHz, CDCl_3_): δ = 175.3 (C–28), 171.1 (C–35), 170.4 (C–31), 170.2 (C–33), 143.9 (C–13), 126.1 (C–12), 73.6 (C–3), 69.1 (C–2), 67.9 (C–6), 65.0 (C–23), 58.8 (C–18), 48.8 (C–37), 48.8 (C–40), 46.1 (C–17), 45.9 (C–9), 45.8 (C–4), 45.5 (C–5), 45.4 (C–10), 45.0 (C–38), 45.0 (C–39), 42.3 (C–1), 40.5 (C–20), 39.5 (C–19), 38.7 (C–8), 38.3 (C–7), 38.3 (C–14), 32.3 (C–22), 30.4 (C–21), 30.3 (C–16), 27.4 (C–15), 25.4 (C–11), 23.4 (C–25), 23.3 (C–27), 22.4 (C–26), 22.3 (C–30), 21.9 (C–34), 21.2 (C–29), 20.9 (C–32), 20.6 (C–36), 17.5 (C–24) ppm; and MS (ESI): *m*/*z* (%) 681.3 ([M + H−H_2_O]^+^, 90); analysis calculated for C_40_H_62_N_2_O_8_ (698.94): C 68.74, H 8.94, N 4.01; found: C 68.45, H 9.13, N 3.83.

### 3.22. (2α,3β,4α,6β) 2,3,23-Tris(acetyloxy)-N-(homopiperazinyl)-6-hydroxyurs-12-en-28-amide (**18**)

Following GPB from **6** (500 mg, 0.79 mmol) with homopiperazine (254 mg, 2.37 mmol) followed by chromatography (SiO_2_, chloroform/methanol, 9:1), **18** (437 mg, 81%) was obtained as a colorless solid; m.p.: 146.5–150.5 °C; ${\left[\alpha \right]}_{D}^{20}$ = +14.38 (*c* = 0.142 CHCl_3_); R_F_ = 0.45 (SiO_2_, CHCl_3_/MeOH, 9:1); IR (ATR): ν = 2943*m*, 1731*s*, 1620*m*, 1464*m*, 1402*m*, 1371*m*, 1237*vs*, 1210*m*, 1180*m*, 1044*m*, 1029*s*, 976*m*, 938*m*, 749*s* cm^−1^; ^1^H NMR (500 MHz, CDCl_3_): δ = 5.24–5.09 (m, 2H, 12–H, 2–H), 5.08–5.01 (m, 1H, 3–H), 3.83 (d, *J* = 11.8 Hz, 1H, 23–H_a_), 3.55 (d, *J* = 11.8 Hz, 1H, 23–H_b_), 4.02–2.87 (m, 10H, 38–H + 41–H + 40–H + 37–H + 39–H), 2.50–2.11 (m, 2H, 18–H + 16–H_a_), 2.06 (s, 3H, 32–H), 1.99 (s, 3H, 36–H), 2.09–1.84 (m, 3H, 1–H + 11–H), 1.95 (s, 3H, 34–H), 1.83–1.68 (m, 1H, 22–H_a_), 1.66–1.54 (m, 2H, 9–H + 22–H_b_), 1.53–1.18 (m, 8H, 21–H + 7–H + 19–H + 6–H + 5–H), 1.15–0.98 (m, 3H, 1–H_b_ + 15–H), 1.07 (s, 3H, 25–H), 1.05 (s, 3H, 27–H), 0.93 (d, *J* = 5.9 Hz, 3H, 30–H), 0.86 (s, 3H, 24–H), 0.84 (d, *J* = 5.7 Hz, 3H, 29–H), 0.70 (s, 3H, 26–H) ppm; ^13^C NMR (125 MHz, CDCl_3_): δ = 176.6 (C–28), 171.0 (C–35), 170.5 (C–31), 170.5 (C–33), 138.7 (C–13), 124.9 (C–12), 75.0 (C–3), 70.0 (C–2), 65.4 (C–23), 55.2 (C–18), 49.1 (C–41), 47.8 (C–40), 47.7 (C–39), 47.7 (C–5), 47.7 (C–9), 47.7 (C–38), 46.5 (C–17), 43.8 (C–1), 43.8 (C–37), 42.4 (C–14), 42.0 (C–4), 39.5 (C–19), 38.7 (C–20), 38.0 (C–8), 37.9 (C–10), 34.4 (C–22), 32.5 (C–7), 30.5 (C–21), 28.1 (C–15), 26.1 (C–16), 23.4 (C–11), 23.4 (C–27), 21.3 (C–30), 21.2 (C–34), 21.0 (C–36), 20.9 (C–32), 18.0 (C–6), 17.4 (C–29), 17.2 (C–25), 17.1 (C–26), 14.0 (C–24) ppm; and MS (ESI): *m*/*z* (%) 701.5 ([M + H]^+^, 100); analysis calculated for C_40_H_64_N_2_O_8_ (712.97): C 69.07, H 9.05, N 3.93; found: C 68.86, H 9.28, N 3.70.

### 3.23. N-[9-[2-({4-(3β,4α)-3-Acetyloxy-23,28-dioxours-12-en-28-yl]-1-piperazinyl}carbonyl)phenyl]-6-diethylamino-3H-xanthen-3-ylidene]-N-ethylethanaminium Chloride (**19**)

Following GPC from **9** (186 mg, 0.32 mmol) followed by chromatography (SiO_2_, chloroform/methanol, 9:1), **19** (213 mg, 64%) was obtained as a purple solid; m.p.: 243–247 °C; R_F_ = 0.29 (SiO_2_, CHCl_3_/MeOH, 9:1); IR (ATR): ν = 2928*w*, 1633*m*, 1586*vs,* 1481*m*, 1411*s*, 1335*s*, 1273*m*, 1244*s*, 1179*vs*, 1132*s,* 1044*w*, 921*m*, 788*w*, 482*w*, 410*w* cm^−1^; UV-Vis (CHCl_3_): λ_max_ (log ε) = 260 (4.54), 307 (4.20), 356 (3.90), 561 (5.08) nm; ^1^H NMR (400 MHz, CDCl_3_): δ = 9.25 (*s*, 1H, 23–H), 7.71–7.59 (*m*, 1H, 38–H), 7.56–7.46 (*m*, 1H, 37–H), 7.37–7.20 (*m*, 1H, 39–H), 7.16–6.89 (*m*, 2H, 44–H + 44′–H), 6.82–6.70 (*m*, 2H, 47–H + 47′–H), 5.23–5.17 (*m*, 1H, 12–H), 4.99–4.90 (*m*, 1H, 3–H), 3.84–3.13 (*m*, 16H, 49–H + 49′–H + 34–H + 33–H), 3.07–2.80 (*m*, 1H, 18–H), 2.20–1.98 (*m*, 1H, 16–H_a_), 1.93 (*s*, 3H, 32–H), 1.91–1.82 (*m*, 2H, 11–H), 1.78–1.51 (*m*, 6H, 2–H + 1–H_a_ + 19–H_a_ + 9–H + 22–H_a_), 1.49–1.36 (*m*, 3H, 6–H_a_ + 15–H_a_ + 7–H_a_), 1.29 (*t*, *J* = 7.0 Hz, 13H, 49–H + 49′–H + 5–H), 1.25–1.16 (*m*, 2H, 22–H_b_), 1.09 (*s*, 3H, 27–H), 1.16–1.00 (*m*, 4H, 19–H_b_ + 21–H_b_ + 7–H_b_ + 1–H_b_), 1.04 (*s*, 3H, 23–H), 0.92 (*s*, 3H, 25–H), 1.00–0.78 (*m*, 3H, 15–H), 0.85 (*s*, 3H, 30–H), 0.83 (*s*, 3H, 29–H), 0.63 (*s*, 3H, 26–H) ppm; ^13^C NMR (100 MHz, CDCl_3_): δ = 204.6 (C–24), 175.7 (C–28), 170.4 (C–31), 167.8 (C–35), 157.8 (C–48), 157.8 (C–48′),155.9 (C–46), 155.8 (C–46′), 155.7 (C–42), 144.9 (C–13), 135.1 (C–41), 132.4 (C–45), 132.4 (C–45′), 130.8 (C–36), 130.4 (C–39), 130.3 (C–40), 130.3 (C–38), 127.7 (C–37), 121.1 (C–12), 114.4 (C–44), 114.4 (C–44′), 113.9 (C–43), 113.9 (C–43′), 96.4 (C–47), 96.4 (C–47′), 73.5 (C–3), 54.4 (C–4), 47.9 (C–5), 47.9 (C–34), 47.9 (C–41), 47.7 (C–9), 47.6 (C–17), 46.3 (C–49), 46.3 (C–49′), 46.3 (C–19), 43.7 (C–18), 42.0 (C–14), 42.0 (C–33), 42.0 (C–39), 39.5 (C–8), 37.8 (C–1), 36.0 (C–10), 34.0 (C–21), 33.0 (C–29), 32.3 (C–7), 30.4 (C–20), 29.8 (C–22), 27.9 (C–15), 25.9 (C–27), 24.1 (C–30), 23.4 (C–11), 22.6 (C–2), 22.4 (C–16), 21.1 (C–32), 20.5 (C–6), 16.98 (C–26), 15.7 (C–25), 12.8 (C–50), 12.8 (C–50′), 9.5 (C–24) ppm; and MS (ESI): *m*/*z* (%) 1005.7 ([M−Cl]^+^, 100); analysis calculated for C_64_H_85_ClN_4_O_6_ (1041.86): C 73.78, H 8.22, N 5.38; found: C 73.49, H 8.47, N 5.08.

### 3.24. N-[9-[2-({4-(3β, 4α)-3-Acetyloxy-23,28-dioxours-12-en-28-yl]-1,4-diazepan-1-yl}carbonyl)phenyl]-6-diethylamino-3H-xanthen-3-ylidene]-N-ethylethanaminium Chloride (**20**)

Following GPC from **10** (176 mg, 0.30 mmol) followed by chromatography (SiO_2_, chloroform/methanol, 9:1), **20** (171 mg, 56%) was obtained as a purple solid; m.p.: 270–276 °C; R_F_ = 0.29 (SiO_2_, CHCl_3_/MeOH, 9:1); IR (ATR): ν = 2927*w*, 1739*m*, 1650*w*, 1644*w*, 1625*m*, 1587*vs*, 1529*w*, 1468*m*, 1412*s*, 1381*m*, 1336*s*, 1245*s*, 1180*vs*, 1074*m*, 975*m*, 824*w*, 748*m*, 620*w*, 472*w*, 412*w* cm^−1^; UV-Vis (CHCl_3_): λ_max_ (log ε) = λ_max_ (log ε) = 260 (4.19), 308 (3.89), 356 (3.59), 562 (4.75) nm; ^1^H NMR (400 MHz, CDCl_3_): δ = 9.25 (s, 1H, 24–H), 7.73–7.53 (m, 1H, 41–H), 7.49–7.37 (m, 1H, 40–H), 7.36–7.08 (m, 3H, 48–H + 42–H), 7.04–6.58 (m, 2H, 50–H + 50′–H), 5.31–5.16 (m, 1H, 12–H), 5.05–4.86 (m, 1H, 3–H), 4.22–3.47 (m, 8H, 52–H + 52′–H + 33–H + 35–H + 37–H), 3.45–2.94 (m, 1H, 34–H + 18–H), 2.41–2.17 (m, 2H, 36–H), 1.93 (s, 3H, 32–H), 2.15–1.80 (m, 2H, 11–H), 1.79–1.56 (m, 6H, 16–H + 1–H_a_ + 19–H_a_ + 22–H_a_ + 9–H), 1.56–1.24 (m, 4H, 7–H_a_ + 5–H + 2–H), 1.31 (t, *J* = 6.2 Hz, 12H, 53–H + 53′–H), 1.23–0.99 (m, 5H, 21–H + 7–H_b_ + 19–H_b_ + 1–H_a_), 1.10 (s, 3H, 27–H), 1.04 (s, 3H, 23–H), 1.04–0.80 (m, 15–H + 6–H), 0.94 (s, 3H, 25–H), 0.90 (s, 3H, 30–H), 0.85 (s, 3H, 29–H), 0.67 (s, 3H, 26–H) ppm; ^13^C NMR (100 MHz, CDCl_3_): δ = 204.5 (C–24), 175.6 (C–28), 170.3 (C–31), 167.7 (C–38), 157.7 (C–51), 157.7 (C–51′), 155.8 (C–49), 155.7 (C-49′), 155.6 (C–45), 144.8 (C–13), 135.0 (C–44), 132.3 (C–48), 130.7 (C–39), 130.3 (C–43), 130.2 (C–42), 130.2 (C–41), 127.6 (C–40), 121.0 (C–12), 114.3 (C–47), 114.3 (C–47′), 113.8 (C–46), 113.8 (C–46′), 96.4 (C–50), 96.3 (C–50′), 73.4 (C–3), 54.3 (C–4), 49.2 (C-35), 47.8 (C–5), 47.6 (C–9), 47.4 (C–17), 47.2 (C-34), 46.5 (C-33 + C-37), 46.2 (C–19), 46.2 (C–52), 46.1 (C–52′), 43.5 (C–18), 41.9 (C–14), 39.4 (C–8), 37.7 (C–1), 35.8 (C–10), 33.9 (C–21), 32.9 (C–29), 32.2 (C–7), 30.3 (C–22), 27.7 (C–15), 26.6 (C-36), 25.8 (C-27), 24.0 (C–30), 23.2 (C–11), 22.4 (C–16), 21.9 (C–2), 21.0 (C–32), 20.4 (C–6), 16.9 (C–26), 15.5 (C–25), 12.7 (C–53), 12.7 (C–53′), 9.4 (C–23) ppm; and MS (ESI): *m*/*z* (%) 1019.1 ([M−Cl]^+^, 100); analysis calculated for C_65_H_87_ClN_4_O_6_ (1055.88): C 73.94, H 8.31, N 5.31; found: C 73.76, H 8.57, N 5.05.

### 3.25. N-9-[2-({4-[(3β,4α)-3,23-Bis(acetyloxy)-28-oxours-12-en-28-yl]-1-piperazinyl}carbonyl)phenyl]-6-diethylamino-3H-xanthen-3-ylidene]-N-ethylethanaminium Chloride (**21**)

Following GPC from **13** (500 mg, 0.80 mmol) followed by chromatography (SiO_2_, chloroform/methanol, 9:1), **21** (237 mg, 47%) was obtained as a purple solid; m.p.: 236–243 °C; R_F_ = 0.19 (SiO_2_, CHCl_3_/MeOH 95:5); IR (ATR): ν = 3340*br*, 2938*br*, 1728*s*, 1585*vs*, 1338*vs*, 1240*vs*, 1005*s*, 686*w* cm^−1^; UV-Vis (CHCl_3_): λ_max_ (log ε) = 258 (0.3), 306 (0.1), 354 (0.1), 561 (1.0) nm; ^1^H NMR (500 MHz, CDCl_3_): δ = 7.72–7.43 (*m*, 3H, 42–H + 43–H + 41–H), 7.38–7.29 (*m*, 1H, 44–H), 7.36–7.24 (*m*, 2H, 49–H + 49′–H), 7.04 (*d*, *J* = 37.5 Hz, 2H, 48–H + 48′–H), 6.83–6.63 (*m*, 2H, 51–H + 51′–H), 5.21 (*d*, *J* = 3.6 Hz, 1H, 12–H), 4.76 (*dd*, *J* = 11.5, 4.9 Hz, 1H, 3–H), 3.96–3.80 (*m*, 1H, 23–H_a_), 3.69–3.57 (*m*, 3H, 23–H_b_ + 53–H + 53′–H), 3.41–3.32 (*m*, 2H, 36–H_a_ + 36–H_b_ + 37–H_a_ + 37–H_b_), 3.27–3.22 (*m*, 2H, 35–H_a_ + 35–H_b_ + 38–H_a_ + 38–H_b_), 3.02–2.83 (*m*, 1H, 18–H), 2.63–2.40 (*m*, 1H, 5–H), 2.05 (*d*, *J* = 2.6 Hz, 3H, 32–H), 2.02 (*s*, 3H, 34–H), 1.96–1.75 (*m*, 2H, 11–H_a_ + 11–H_b_), 1.72–1.38 (*m*, 14H, 16–H_a_ + 16–H_b_ + 19–H_a_ + 7–H_a_ + 7–H_b_ + 2–H_a_ + 2–H_b_ + 1–H_a_ + 9–H + 6–H_a_ + 6–H_b_ + 15–H_a_ + 22–H_a_ + 21–H_a_), 1.31 (*t*, *J* = 6.9 Hz, 2H, 54–H + 54′–H), 1.24–1.08 (*m*, 5H, 19–H_b_ + 15–H_b_ + 22–H_b_ + 1–H_b_ + 21–H_b_), 1.07 (*s*, 3H, 27–H), 1.00 (*d*, *J* = 23.5 Hz, 3H, 29–H), 0.92 (*s*, 3H, 25–H), 0.86 (*d*, *J* = 1.9 Hz, 3H, 30–H), 0.81 (*s*, 3H, 24–H), 0.64 (*s*, 3H, 26–H) ppm; ^13^C NMR (125 MHz, CDCl_3_): δ = 175.7 (C–28), 171.0 (C–31), 170.6 (C–33), 167.7 (C–37), 157.7 (C–52), 157.7 (C–52′), 155.8 (C–46), 155.7 (C–50), 155.6 (C–50′), 144.6 (C–13), 135.1 (C–45), 132.3 (C–49), 132.3 (C–49′), 130.7 (C–40), 130.4 (C–44), 130.2 (C–42), 130.2 (C–43), 127.6 (C–41), 121.4 (C–12), 114.3 (C–48), 114.3 (C–48′), 113.8 (C–47), 113.8 (C–47′), 96.3 (C–51), 96.3 (C–51′), 74.5 (C–3), 65.4 (C–23), 55.8 (C–5), 47.9 (C–9), 47.7 (C–17), 47.5 (C–36), 47.5 (C–37), 46.2 (C–19), 46.1 (C–53), 46.1 (C–53′), 45.4 (C–4), 43.5 (C–18), 41.7 (C–35), 41.7 (C–38), 40.5 (C–14), 39.0 (C–8), 37.7 (C–1) 36.8 (C–10), 34.0 (C–21), 32.9 (C–30), 32.5 (C–7), 32.3 (C–20), 30.3 (C–15), 29.9 (C–2), 27.8 (C–27), 25.7 (C–29), 24.0 (C–11), 23.4 (C–16), 22.9 (C–22), 21.2 (C–32), 20.9 (C–34), 17.9 (C–6), 16.9 (C–26), 15.8 (C–25), 13.1 (C–24) 12.7 (C–54), 12.7 (C–54′) ppm; and MS (ESI): *m*/*z* (%) 1049.86 ([M−Cl]^+^, 60); analysis calculated for C_66_H_89_ClN_4_O_7_ (1085.91): C 73.00, H 8.26, N 5.16; found: C 72.84, H 8.41, N 4.87.

### 3.26. N-9-[2-({4-[(3β,4α)-3,23-Bis(acetyloxy)-28-oxours-12-en-28-yl]-1,4-diazepan-1-yl}carbonyl)phenyl]-6-diethylamino-3H-xanthen-3-ylidene]-N-ethylethanaminium Chloride (**22**)

Following GPC from **14** (156 mg, 0.24 mmol) followed by chromatography (SiO_2_, chloroform/methanol, 9:1), **22** (49 mg, 31%) was obtained as a purple solid; m.p.: 243–248 °C; R_F_ = 0.29 (SiO_2_, CHCl_3_/MeOH, 9:1); IR (ATR): ν = 3424*br*, 2941*br*, 1732*s*, 1640*w*,1588*s*, 1338*w*, 1244*s*, 690*w* cm^−1^; UV-Vis (CHCl_3_): λ_max_ (log ε) = 256 (0.2), 304 (0.1), 354 (0.1), 558 (0.5) nm; ^1^H NMR (500 MHz, CDCl_3_): δ = 7.84–7.68 (*m*, 1H, 42–H), 7.67–7.55 (*m*, 2H, 43–H + 44–H), 7.43 (*d*, *J* = 6.6 Hz, 1H, 45–H), 7.26 (*s*, 2H, 50–H + 50′–H), 7.05 (*t*, *J* = 11.1 Hz, 2H, 49–H + 49′–H), 6.89–6.70 (*m*, 2H, 52–H + 52′–H), 5.31–5.17 (*m*, 1H, 12–H), 4.80–4.69 (*m*, 1H, 3–H), 3.84 (*t*, *J* = 10.3 Hz, 1H, 23–H_a_), 3.74–3.40 (*m*, 5H, 23–H_b_ + 54–H+ 54′–H + 39–H_a_ + 39–H_b_), 3.13 (*d*, *J* = 75.5 Hz, 2H, 35–H_a_ + 35–H_b_), 2.94–2.82 (*m*, 5H, 18–H + 36–H_a_ + 36–H_b_ + 37–H_a_+ 37–H_b_), 2.18–2.07 (*m*, 1H, 16–H_a_ + 22–H_a_) 2.05 (*s*, 3H, 34–H), 2.00 (*s*, 3H, 32–H), 1.87 (*tq*, *J* = 18.0, 9.1, 8.6 Hz, 2H, 11–H_a_ + 11–H_b_), 1.74–1.35 (*m*, 14H, 16–H_b_ + 19–H_a_ + 19–H_b_ + 15–H_a_ + 38–H_a_ + 38–H_b_ + 2–H_a_ + 2–H_b_ + 22–H_b_ + 1–H_a_ + 7–H_a_ + 6–H_a_ + 6–H_b_ +21–H_a_), 1.26–1.13 (*m*, 5H, 7–H_b_ + 9–H + 1–H_b_ + 21–H_b_ + 15–H_b_ + 55–H + 55′–H), 1.12 (*s*, 3H, 27–H), 0.94 (*s*, 3H, 25–H), 0.93 (*s*, 3H, 29–H), 0.89 (*s*, 3H, 30–H), 0.81 (*s*, 3H, 24–H), 0.74 (*s*, 3H, 26–H) ppm; ^13^C NMR (125 MHz, CDCl_3_): δ = 174.5 (C–28), 171.0 (C–31), 170.7 (C–33), 166.8 (C–40), 157.7 (C–53), 157,7 (C–53′), 155.7 (C–47), 155.7 (C–51), 155,6 (C–51′), 144.7 (C–13), 135.0 (C–46), 132.2 (C–50), 132.2 (C–50′), 130.6 (C–41), 130.4 (C–45),130.3 (C–43), 130.2 (C–44), 127.8 (C–42), 121.4 (C–12), 114.3 (C–49), 114.3 (C–49′), 113.9 (C–48), 113.9 (C–48′), 96.2 (C–52), 96.2 (C–52′), 74.6 (C–3), 65.5 (C–23), 56.3 (C–39), 48.0 (C–5), 47.9 (C–9), 47.8 (C–36), 46.6 (C–17), 46.3 (C–37), 46.2 (C–54), 46.2 (C–54′), 45.5 (C–19), 43.7 (C–35), 41.9 (C–4), 40.6 (C–14), 40.5 (C–18), 39.1 (C–8), 37.7 (C–1), 36.8 (C–10), 34.1 (C–21), 33.0 (C–30), 32.4 (C–7), 32.3 (C–20), 30.3 (C–15), 30.0 (C–2), 27.9 (C–27), 25.8 (C–29), 23.9 (C–11), 23.4 (C–16), 22.9 (C–22), 21.2 (C–32), 20.9 (C–34), 18.0 (C–6), 16.8 (C–26), 15.8 (C–25), 13.1 (C–24), 12.9 (C–55), 12.9 (C–55′) ppm; and MS (ESI): *m*/*z* (%) = 1064.1 ([M−Cl]^+^, 70); analysis calculated for C_67_H_91_ClN_4_O_7_ (1099.94): C 73.16, H 8.34, N 5.09; found: C 72.87, H 8.53, N 4.85.

### 3.27. N-{6-Diethylamino-9-[2-({4[ (2α,3β,4α,6β)-2,3,23-tris(acetyloxy)-6-hydroxy-28-oxours-12-en-28-yl)-1-piperazinyl]carbonyl]phenyl]-3H-xanthen-3-ylidene)-N-ethylethanaminium Chloride (**23**)

Following GPC from **17** (156 mg, 0.22 mmol) followed by chromatography (SiO_2_, chloroform/methanol, 9:1), **23** (88 mg, 56%) was obtained as a purple solid; m.p.: 228–233 °C; R_F_ = 0.20 (SiO_2_, CHCl_3_/MeOH, 95:5); IR (ATR): ν = 2915*w*, 2897*w*, 1735*s*, 1568*s*, 1466*s*, 1340*s*, 1232*s*, 1160*vs*, 1077*s* cm^−1^; UV-Vis (CHCl_3_): λ_max_ (log ε) = 256 (0.4), 305 (0.2), 354 (0.1), 560 (1.0) nm; ^1^H NMR (500 MHz, CDCl_3_): δ = 7.84–7.61 (*m*, 2H, 44–H, 45–H), 7.54–7.47 (*m*, 1H, 43–H), 7.35–7.27 (*m*, 3H, 46–H, 51–H, 51′–H), 7.18–6.93 (*m*, 2H, 50–H, 50′–H), 6.92–6.67 (*m*, 2H, 53–H, 53′–H), 5.32–5.24 (*m*, 1H, 12–H), 5.13 (*dd*, *J* = 10.4, 6.7 Hz, 2H, 3–H, 2–H), 4.23 (*dd*, *J* = 11.9, 7.7 Hz, 2H, 6–H, 23–H_a_), 3.78–3.51 (*m*, 3H, 55–H, 55′–H, 23–H_b_), 3.49–3.18 (*m*, 8H, 37–H_a_, 37–H_b_ 40–H_a_, 40–H_b_, 38–H_a_, 38–H_b,_ 39–H_a_, 39–H_b_), 2.23–2.11 (*m*, 1H, 22–H_a_), 2.09 (*s*, 3H, 36–H), 2.16–2.07 (*m*, 2H, 18–H, 16–H_a_), 2.02 (*s*, 3H, 32–H), 2.00 (*s*, 3H, 34–H), 1.98 (*s*, 3H, 1–H_a_, 16–H), 1.92–1.77 (*m*, 2H, 11–H_a_, 11–H_b_), 1.80–1.54 (*m*, 1H, 9–H), 1.66–1.27 (*m*, 14H, 6–H_a_, 6–H_b_, 7–H_a_, 7–H_b_, 16–H_b_, 22–H_b_, 21–H_a_, 1–H_b_, 15–H_a_, 21–H_b_, 20–H, 19–H_a_, 56–H, 56′–H), 1.25 (*s*, 3H, 26–H), 1.22–0.92 (*m*, 2H, 15–H_b_, 19–H_b_), 1.12 (*s*, 3H, 29–H), 0.99 (*s*, 3H, 25–H), 0.93 (*s*, 3H, 27–H), 0.86 (*s*, 3H, 30–H), 0.82–0.75 (*m*, 1H, 5–H), 0.83 (*s*, 3H, 24–H) ppm; ^13^C NMR (125 MHz, CDCl_3_): δ = 174.6 (C–28), 171.0 (C–35), 170.5 (C–31), 170.2 (C–33), 167.7 (C–41), 157.7 (C–54), 157.7 (C–54′), 155.9 (C–48), 155.6 (C–52), 155.6 (C–52′), 144.7 (C–13), 135.0 (C–47), 132.3 (C–51), 132.3 (C–51′), 130.7 (C–42), 130.3 (C–46), 130.2 (C–44), 130.1 (C–45), 127.7 (C–43), 126.0 (C–12), 114.3 (C–50), 114.3 (C–50′), 113.9 (C–49), 113.9 (C–49′), 96.2 (C–53), 96.2 (C–53′), 73.5 (C–3), 69.1 (C–2), 67.5 (C–6), 64.9 (C–23), 58.7 (C–18), 48.7 (C–37), 48.7 (C–39), 46.2 (C–17), 45.9 (C–55), 45.9 (C–55′), 45.9 (C–9), 45.7 (C–4), 45.5 (C–5), 45.3 (C–10), 45.0 (C–38), 45.0 (C–39), 42.3 (C–1), 40.6 (C–20), 39.3 (C–19), 38.6 (C–8), 38.3 (C–7), 38.2 (C–14), 32.3 (C–22), 30.4 (C–21), 30.3 (C–16), 27.4 (C–15), 25.4 (C–11), 23.4 (C–25), 23.3 (C–27), 22.4 (C–26), 22.3 (C–30), 21.9 (C–34), 21.1 (C–29), 20.9 (C–32), 20.7 (C–36) 17.4 (C–24), 12.7 (C–56), 12.7 (C–56′) ppm; and MS (ESI): *m*/*z* (%) = 1123.7 ([M−Cl]^−^, 35); analysis calculated for C_68_H_91_ClN_4_O_10_ (1159.94): C 70.41, H 7.91, N 4.83; found: C 7021, H 8.13, N 4.74.

### 3.28. N-{6-Diethylamino-9-[2-({4[ (2α,3β,4α,6β)-2,3,23-tris(acetyloxy)-6-hydroxy-28-oxours-12-en-28-yl)-1,4-diazepan-1-yl}carbonyl]phenyl]-3H-xanthen-3-ylidene)-N-ethylethanaminium Chloride (**24**)

Following GPC from **18** (250 mg, 0.35 mmol) followed by chromatography (SiO_2_, chloroform/methanol, 9:1), **24** (197 mg, 48%) was obtained as a purple solid; m.p.: 269–273 °C; R_F_ = 0.34 (SiO_2_, CHCl_3_/MeOH 9:1); IR (ATR): ν = 2925*w*, 1739*m*, 1645*w*, 1626*m*, 1587*vs*, 1412*s*, 1395*m*, 1336*s*, 1244*s*, 1179*vs*, 1160*m*, 1073*m*, 1029*m*, 976*m*, 746*s*, 683*s*, 472*w*, 410*w* cm^−1^; UV-Vis (CHCl_3_): λ_max_ (log ε) = 260 (4.43), 307 (4.10), 355 (3.80), 562 (4.98) nm; ^1^H NMR (500 MHz, CDCl_3_): δ = 7.72–7.54 (*m*, 2H, 46–H + 45–H), 7.50–7.37 (*m*, 1H, 44–H), 7.33–7.07 (*m*, 3H, 47–H + 51–H + 51′–H), 6.96–6.62 (*m*, 2H, 54–H + 54′–H), 5.58–5.46 (*m*, 1H, 12–H), 5.38–5.20 (*m*, 1H, 2–H), 5.11 (*d*, *J* = 10.4 Hz, 1H, 3–H), 4.21 (*d*, *J* = 11.8 Hz, 1H, 23–H_a_), 4.15–2.81 (*m*, 19H, 56–H + 56′–H + 23–H_b_ + 37–H + 38–H + 39–H + 40–H + 41–H), 2.52–2.22 (*m*, 4H, 18–H + 22–H_a_ + 16–H), 2.20–1.84 (*m*, 3H, 1–H_a_ + 11–H), 2.02 (*s*, 3H, 36–H), 1.97 (*s*, 3H, 34–H), 1.96 (*s*, 3H, 32–H), 1.83–1.66 (*m*, 1H, 9–H), 1.64–1.50 (*m*, 1H, 22–H_b_), 1.31 (*s*, 12H, 57–H + 57′–H), 1.49–1.22 (*m*, 5H, 21–H_b_ + 1–H_b_ + 19–H + 6–H +20–H), 1.18 (*s*, 3H, 25–H), 1.10 (*s*, 3H, 24–H), 1.03–0.87 (*m*, 1H, 5–H), 0.93 (*s*, 3H, 27–H), 0.91 (*s*, 3H, 29–H), 0.83 (*s*, 3H, 26–H), 0.82 (*s*, 3H, 30–H) ppm; ^13^C NMR (125 MHz, CDCl_3_): δ = 176.8 (C–28), 171.1 (C–35), 170.5 (C–31), 170.4 (C–33), 168.1 (C–42), 157.9 (C–55), 157.9 (C–55′), 155.9 (C–53), 155.9 (C–53′), 155.8 (C–49), 143.1 (C–13), 134.1 (C–48), 132.5 (C–51), 132.5 (C–51′), 131.4 (C–52), 131.4 (C–52′), 130.2 (C–46), 130.1 (C–47), 129.7 (C–45), 126.9 (C–44), 122.7 (C–12), 114.2 (C–51), 114.2 (C–51′), 114.0 (C–50), 113.8 (C–50′), 96.4 (C–54), 96.4 (C–54′), 73.7 (C–3), 69.3 (C–2), 65.1 (C–23), 55.8 (C–18), 49.4 (C–37), 49.4 (C–41), 46.6 (C–40), 46.4 (C–39), 46.3 (C–56), 46.6 (C–56′), 46.2 (C–38), 46.2 (C-17), 46.0 (C-9), 45.1 (C-4), 43.9 (C-14),42.4 (C-1), 38.8 (C-19), 38.6 (C-5), 38.5 (C-20), 38.4 (C-6), 38.4 (C-8), 38.3 (C-10), 34.2 (C-7),32.4 (C-22), 30.4 (C-21), 27.4 (C-15), 25.4 (C-16), 23.5 (C-11), 23.1 (C-27), 22.4 (C-25), 22.0 (C-24), 21.3 (C-29), 21.2 (C-26), 21.0 (C-34), 21.0 (C-32), 20.8 (C-36), 17.7 (C-30), 12.8 (C-57 + C-57′) ppm; and MS (ESI): *m*/*z* (%) 1137.7 ([M−Cl]^+^, 25); analysis calculated for C_69_H_93_ClN_4_O_10_ (1173.97): C 70.59, H 7.99, N 4.77; found: C 70.33, H 8.16, N 4.52.

## 4. Conclusions

Several amides and rhodamine B conjugates derived from parent triterpenoic acids gypsogenin, hederagenin, and madecassic acid were synthesized and their cytotoxicity was evaluated in SRB assay. While the parent acids exhibited very low cytotoxicity, their corresponding acetates proved better. Acetylated amides, however, gave significantly lower EC_50_ values than the acetates. A significant increase in cytotoxicity was observed for the corresponding rhodamine B conjugates obtained from the latter. While rhodamine B is not cytotoxic (cut-off 30 μM), triterpenoic conjugates of rhodamine B holding a piperazinyl or homopiperazinyl spacer were cytotoxic, even in nano-molar concentration, with compound **24** being the most cytotoxic one. In comparison, homopiperazinyl-spacered compounds were more tumor-cell selective than their piperazinyl-spacered analogs. The preparation of benzyl and phenylamides succeeded in generating compounds holding similar bioactivity as previously reported for some analogs derived from betulinic, ursolic, glycyrrhetinic, or oleanolic acid. Our present studies showed spacered conjugates derived from madecassic acid of excellent cytotoxicity in single-digit nanomolar concentration combined with a noteworthy tumor/non-tumor cell selectivity.

## Figures and Tables

**Table 1:** SRB assay: EC50 values (µM) after 72 h of treatment; averaged from three independent experiments performed each in triplicate; confidence interval CI = 95%. Human cancer cell lines: A375 (melanoma), HT29 (colorectal carcinoma), MCF7 (breast adenocarcinoma), A2780 (ovarian carcinoma), HeLa (cervical carcinoma), and (NIH 3T3 (non-malignant fibroblasts); cut-off 30 μM, n.d. not determined. Doxorubicin (DX) has been used as a positive standard.

#	A375	HT29	MCF7	A2780	HeLa	NIH 3T3
**1**	17.3 ± 1.6	>30	18.5 ± 2.2	13.0 ± 1.6	25.0 ± 4.1	16.7 ± 2.8
**2**	26.3 ± 1.8	>30	25.7 ± 1.9	19.6 ± 1.6	>30	>30
**3**	>30	>30	>30	>30	n.d.	>30
**4**	>30	>30	24.6 ± 4.7	18.7 ± 2,7	>30	13.0 ± 1,5
**5**	17.1 ± 1.1	19.0 ± 0.6	9.8 ± 1.0	11.9 ± 0.5	12.4 ± 0.7	9.6 ± 0.6
**6**	3.5 ± 0.5	14.5 ± 1.1	7.1 ± 0.6	8.1 ± 0.7	n.d.	7.6 ± 0.8
**7**	3.3 ± 0.3	10.7 ± 1.8	8.8 ± 1.6	4.4 ± 0.3	n.d.	18.5 ± 4.6
**8**	7.9 ± 3.3	>30	>30	19.9 ± 1.4	n.d.	>30
**9**	1.5 ± 0.2	1.7 ± 0.3	2.1 ± 0.3	1.6 ± 0.2	n.d.	2.3 ± 0.3
**10**	1.6 ± 0.6	0.7 ± 0.1	2.0 ± 0.2	1.7 ± 0.5	1.7 ± 0.3	1.4 ± 0.2
**11**	1.1 ± 0.6	3.8 ± 0.7	3.4 ± 0.4	1.3 ± 0.3	n.d.	>30
**12**	0.6 ± 0.2	1.9 ± 0.6	1.9 ± 0.1	0.8 ± 0.2	n.d.	>30
**13**	1.3 ± 0.4	1.9 ± 0.5	1.9 ± 0.4	1.4 ± 0.2	n.d.	2.0 ± 0.3
**14**	1.2 ± 0.3	1.9 ± 0.1	1.7 ± 0.2	2.0 ± 0.2	2.2 ± 0.3	1.7 ± 0.3
**15**	1.0 ± 0.1	2.3 ± 0.2	2.7 ± 0.4	1.4 ± 0.1	n.d.	17.9 ± 2.1
**16**	1.3 ± 0.1	3.7 ± 0.4	4.5 ± 0.9	2.2 ± 0.1	n.d.	21.1 ± 1.1
**17**	1.9 ± 0.4	2.1 ± 0.6	2.0 ± 0.4	2.1 ± 0.3	n.d.	2.4 ± 0.6
**18**	2.2 ± 0.3	2.4 ± 0.4	2.4 ± 0.3	2.8 ± 0.3	n.d.	2.5 ± 0.2
**19**	n.d.	n.d.	n.d.	n.d.	n.d.	n.d.
**20**	0.0319 ±0.0036	0.0566 ±0.0123	0.0558 ± 0.0072	0.0131 ± 0.0007	0.1021 ± 0.0263	0.1600 ± 0.0226
**21**	0.055 ± 0.01	0.086 ± 0.01	0.066 ± 0.008	0.025 ± 0.002	-	0.305 ± 0.06
**22**	0.031 ± 0.007	0.083 ± 0.02	0.086 ± 0.01	0.018 ± 0.001	-	0.441 ± 0.12
**23**	0.055 ± 0.01	0.108 ± 0.02	0.054 ± 0.01	0.015 ± 0.001	-	0.277 ± 0.03
**24**	0.0095 ± 0.0015	0.0127 ± 0.0038	0.0162 ± 0.0045	0.0029 ± 0.0005	0.0526 ± 0.0151	0.1013 ± 0.0231
**DX**	n.d.	0.9 ± 0.2	1.1 ± 0.3	0.02 ± 0.01	n.d.	1.7 ± 0.3

## Data Availability

Not applicable.

## Supplementary Materials

The following are available online at https://www.mdpi.com/article/10.3390/ijms23084362/s1, Supplementary File S1: details of the SRB assay, and depicted NMR (^1^H, ^13^C).
