# Supplementary material for: Madecassic Acid—A New Scaffold for Highly Cytotoxic Agents
Source: Int J Mol Sci. 2022 Apr 14;23(8):4362. doi: 10.3390/ijms23084362 (PMC9026082; doi:10.3390/ijms23084362)

**Supplementary material**

# Madecassic acid – a new scaffold for highly cytotoxic agents

Oliver Kraft [a], Anne-Kathrin Hartmann[a], Sophie Hoenke [a], Immo Serbian [a], René Csuk[*, a]

[a] Martin-Luther-University Halle-Wittenberg, Organic Chemistry, Kurt-Mothes-Str. 2, D-06120 Halle (Saale), Germany

## 1. *General*

NMR spectra were recorded using the Agilent spectrometers DD2 500 MHz and VNMRS 400 MHz (δ given in ppm, J in Hz; typical experiments: APT, H-H-COSY, HMBC, HSQC, NOESY), MS spectra were taken on an Advion Expression CMS instrument. TLC was performed on silica gel (Macherey-Nagel, detection with cerium molybdate reagent); melting points are uncorrected (Leica hot stage microscope, or BUCHI melting point M-565), and elemental analyses were performed on a Foss-Heraeus Vario EL (CHNS) unit. IR spectra were recorded on a Perkin Elmer FT-IR spectrometer Spectrum 1000 or on a Perkin-Elmer Spectrum Two (UATR Two Unit). The solvents were dried according to usual procedures.

## 2. *Cytotoxicity assay (SRB assay)*

The cell lines were obtained from Department of oncology (Martin-Luther-University Halle Wittenberg). Cultures were maintained as monolayers in RPMI 1640 medium with L-glutamine (Capricorn Scientific GmbH, Ebsdorfergrund, Germany) supplemented with 10% heat inactivated fetal bovine serum (Sigma-Aldrich Chemie GmbH, Steinheim, Germany) and penicillin/streptomycin (1%, Capricorn Scientific GmbH, Ebsdorfergrund, Germany) at 37 °C in a humidified atmosphere with 5% $CO_2$.

The cytotoxicity of the compounds was evaluated using the sulforhodamine-B (Kiton-Red S, ABCR) micro culture colorimetric assay using confluent cells in 96-well plates with the seeding of the cells on day 0 applying appropriate cell densities to prevent confluence of the cells during the period of the experiment. On day 1, the cells were treated with six different concentrations (1, 3, 7, 12, 20 and 30 μM); thereby, the final concentration of DMSO was always < 0.5%, generally regarded as non-toxic to the cells. On day 4, the supernatant medium was discarded; the cells were fixed with 10 % trichloroacetic acid. After another day at 4 °C, the cells were washed in a strip washer and dyed with the SRB solution (100 μL, 0.4% in 1% acetic acid) for about 20 min to be followed by washing of the plates (four times, 1% acetic acid) and air-drying

overnight. Furthermore, tris base solution (200 μL, 10 mM) was added to each well and absorbance was measured at λ = 570 nm employing a reader (96 wells, Tecan Spectra, Crailsheim, Germany). The $EC_{50}$ values were averaged from three independent experiments performed each in triplicate calculated from semi logarithmic dose response curves applying a non-linear four-parameter Hills-slope equation (GraphPad Prism5; variables top and bottom were set to 100 and 0, respectively).

**Compound 5:**

$^{1}H$ NMR (500 MHz, $CDCl_3$)

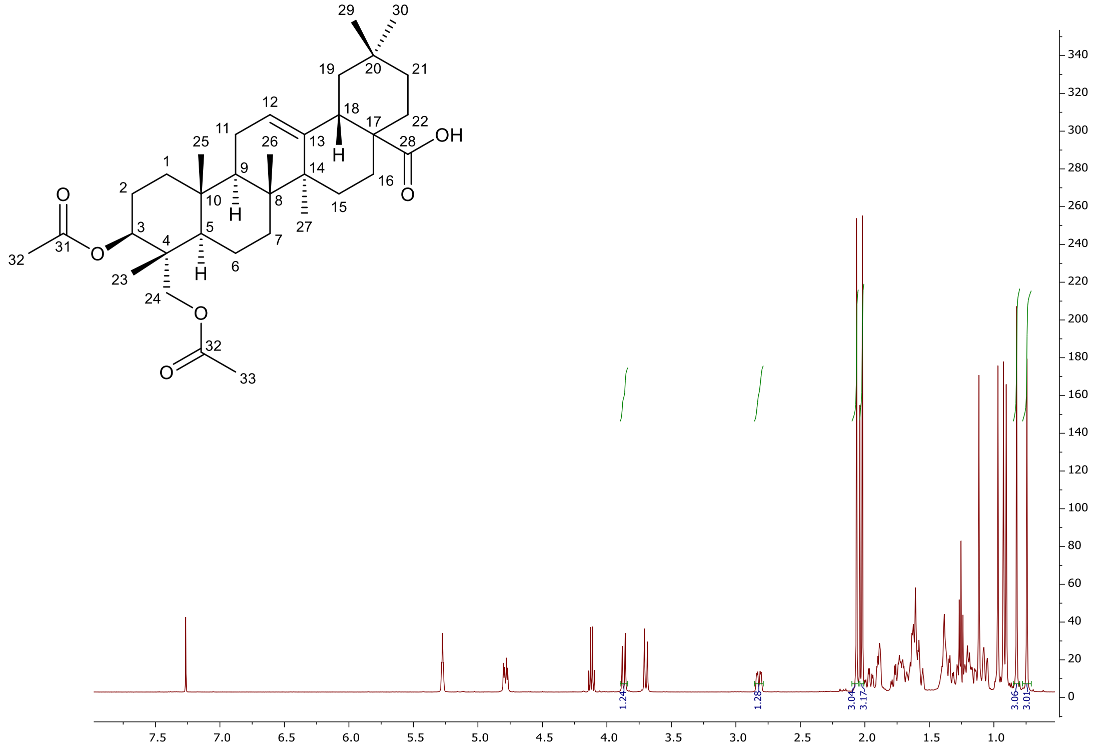


$^{13}C$ NMR (125 MHz, $CDCl_3$)

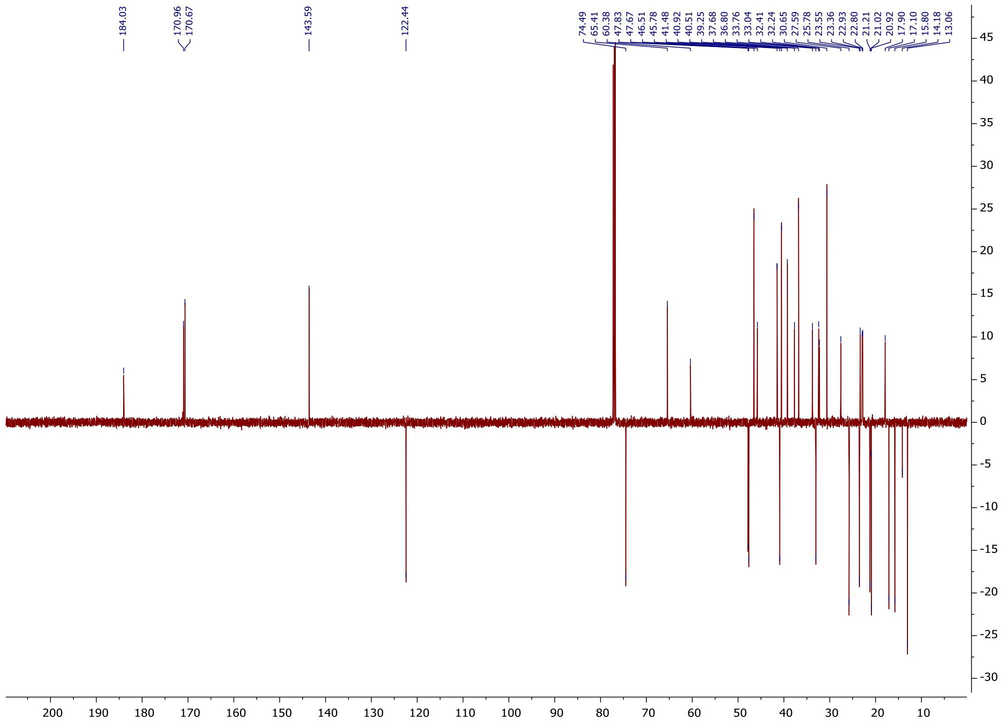

**Compound 6:**

$^{1}$H NMR (400 MHz, $CDCl_3$)

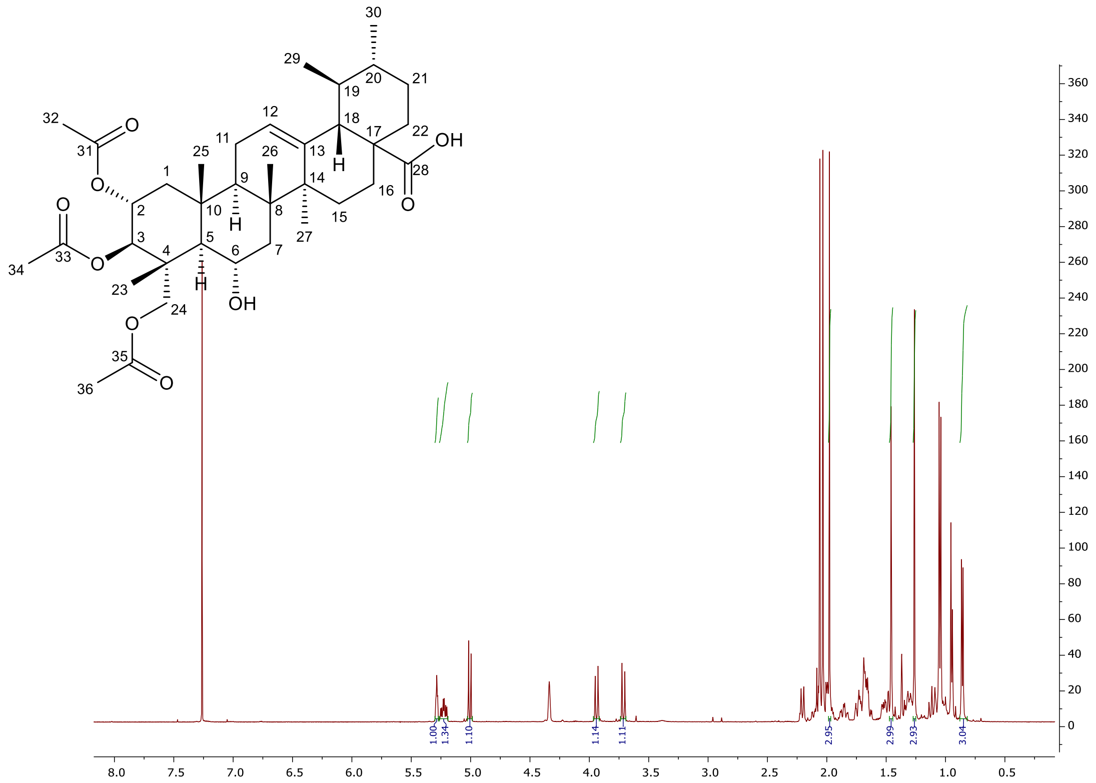


$^{13}$C NMR (100 MHz, $CDCl_3$)

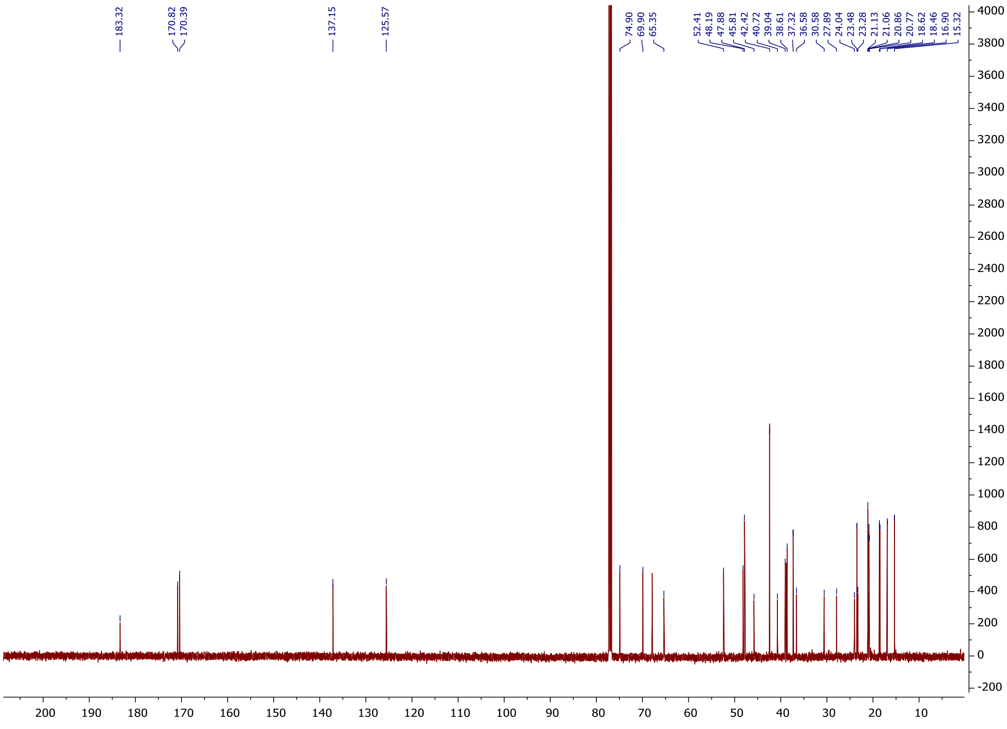

**Compound 7:**

$^{1}$H NMR (400 MHz, $CDCl_3$)

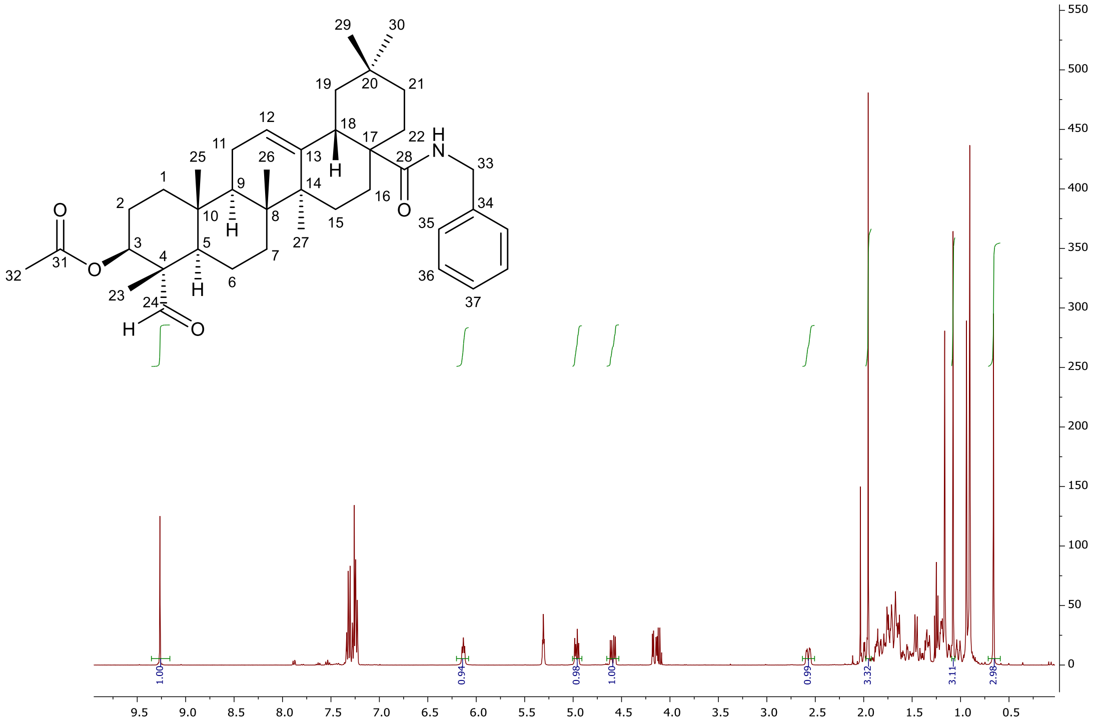


$^{13}$C NMR (100 MHz, $CDCl_3$)

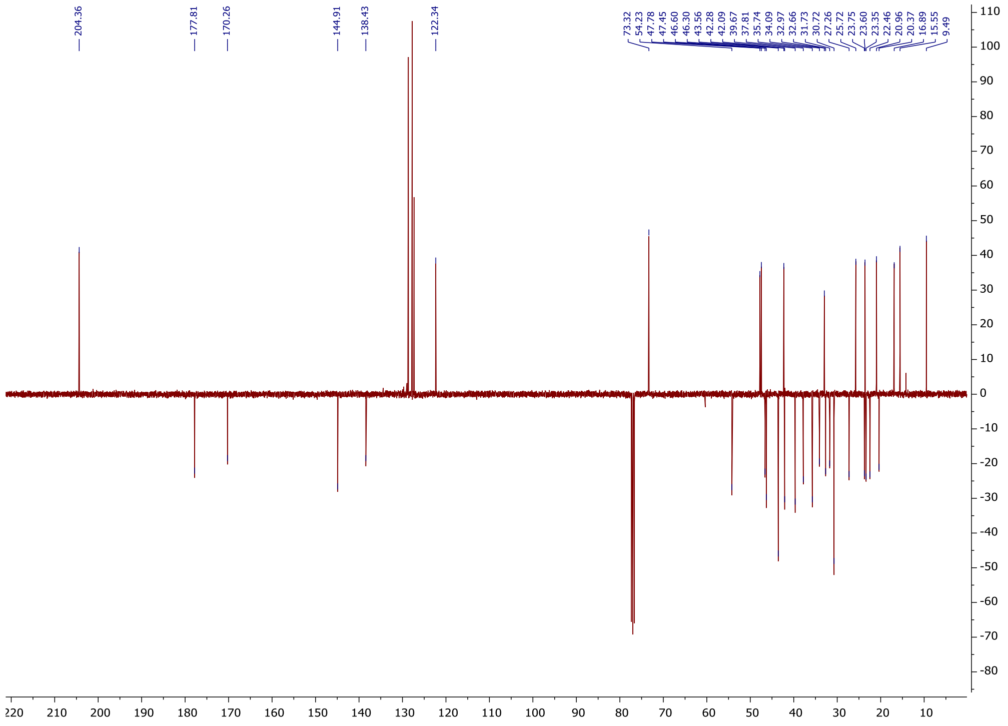

**Compound 8:**

$^{1}H$ NMR (500 MHz, $CDCl_3$)

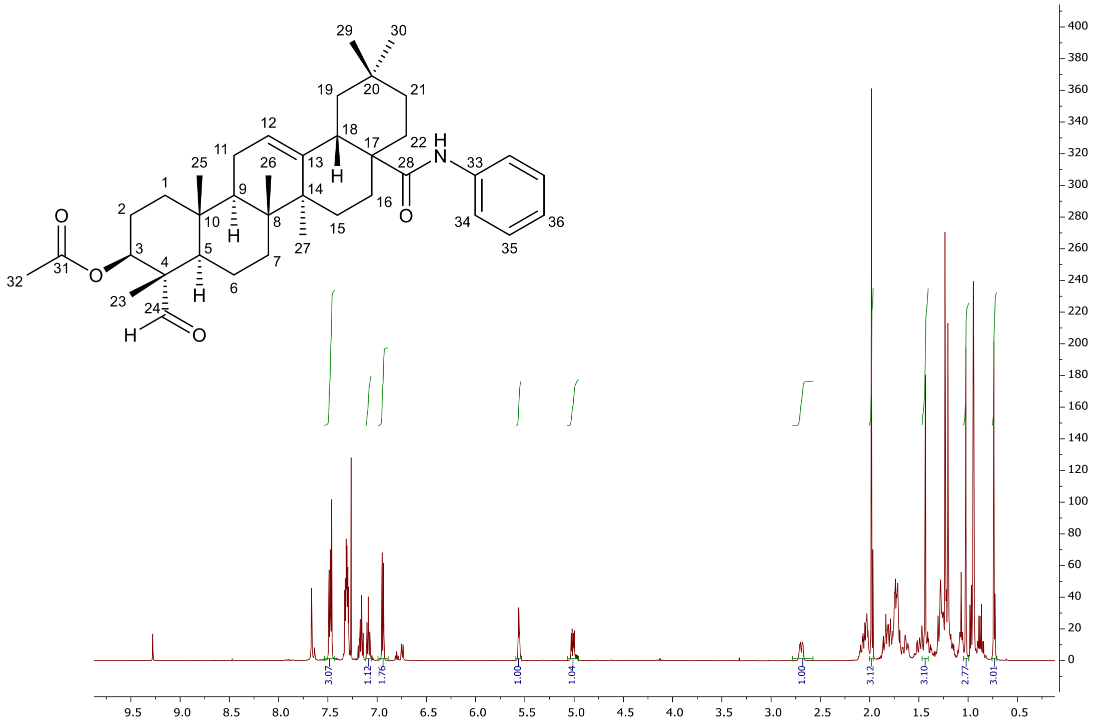


$^{13}C$ NMR (125 MHz, $CDCl_3$)

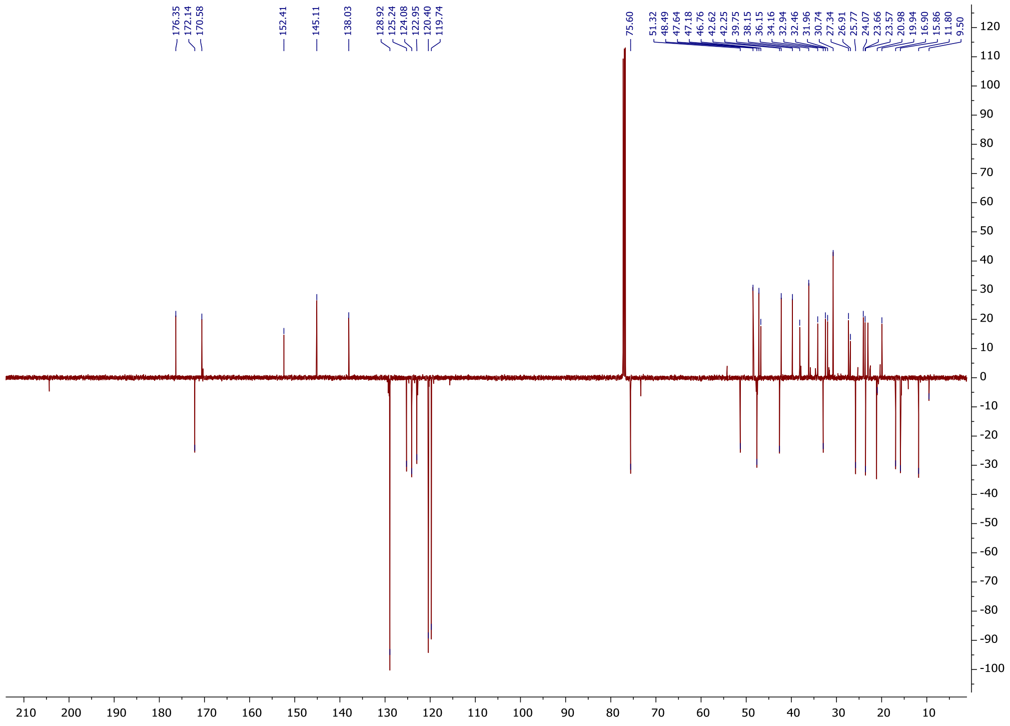

**Compound 9:**

$^{1}$H NMR (400 MHz, $CDCl_3$)

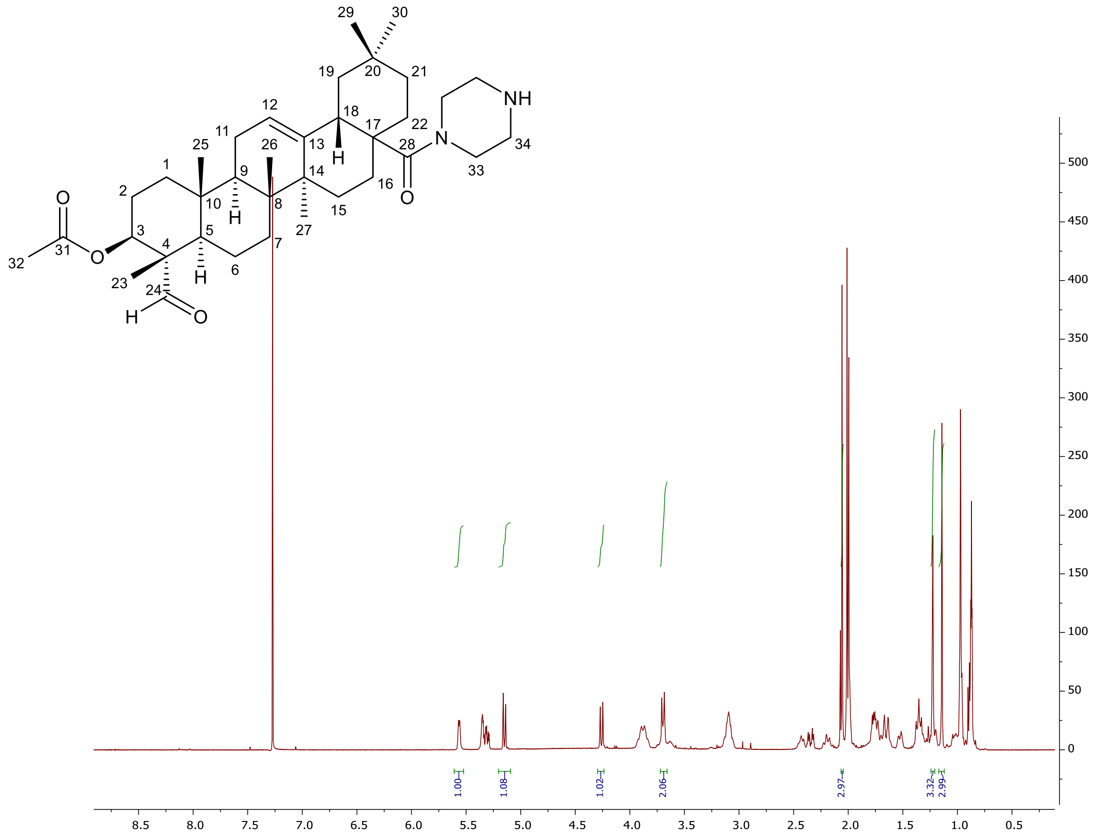


$^{13}$C NMR (100 MHz, $CDCl_3$)

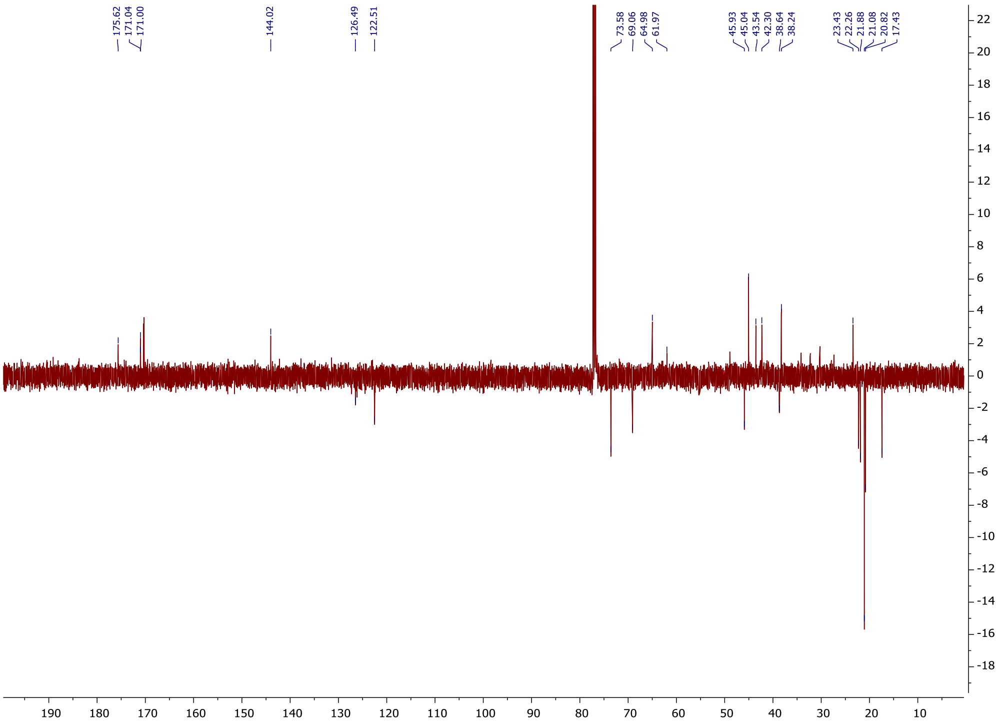

**Compound 10:**

$^{1}$H NMR (400 MHz, $CDCl_3$)

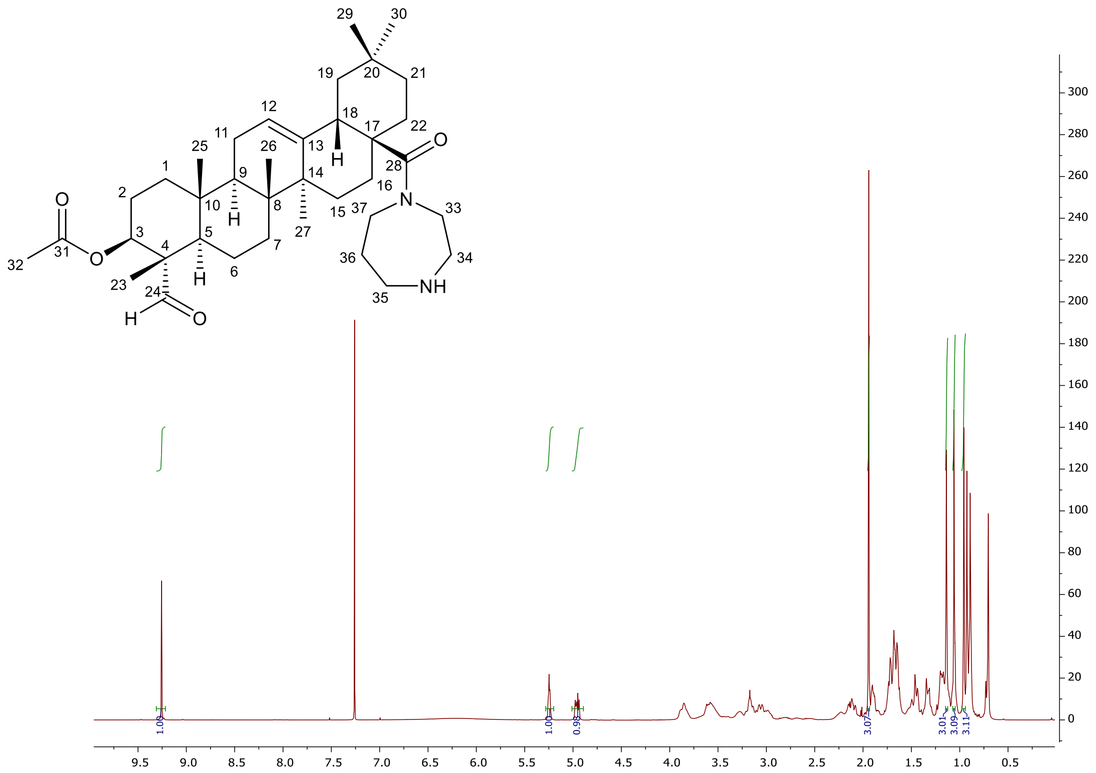

$^{13}$C NMR (100 MHz, $CDCl_3$)

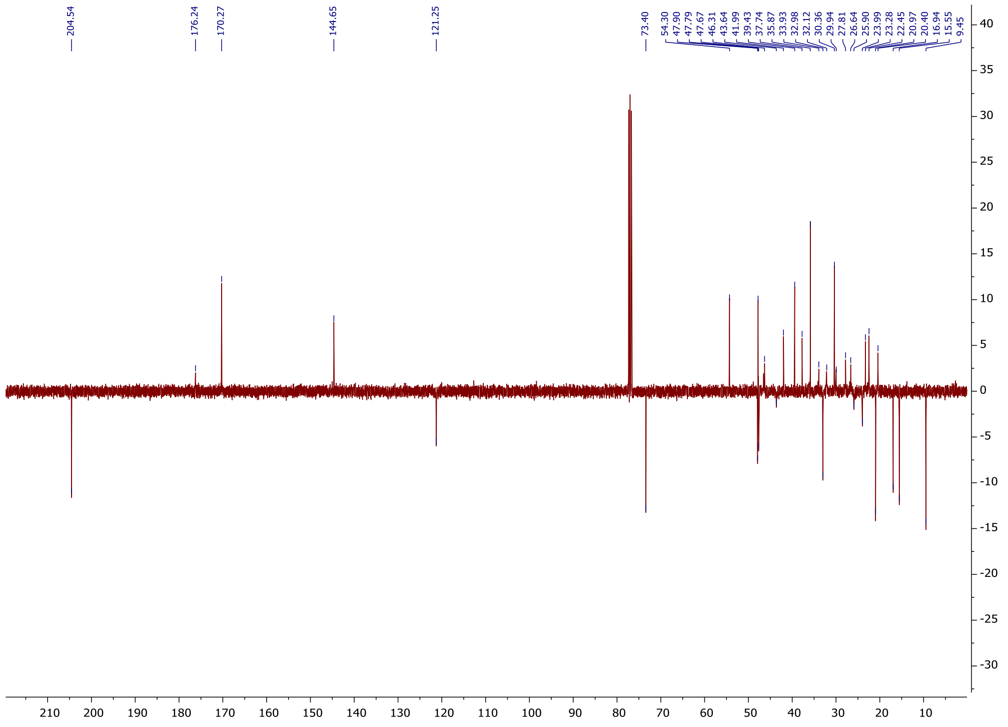

## Compound 11:

$^{1}$H NMR (400 MHz, $CDCl_3$)

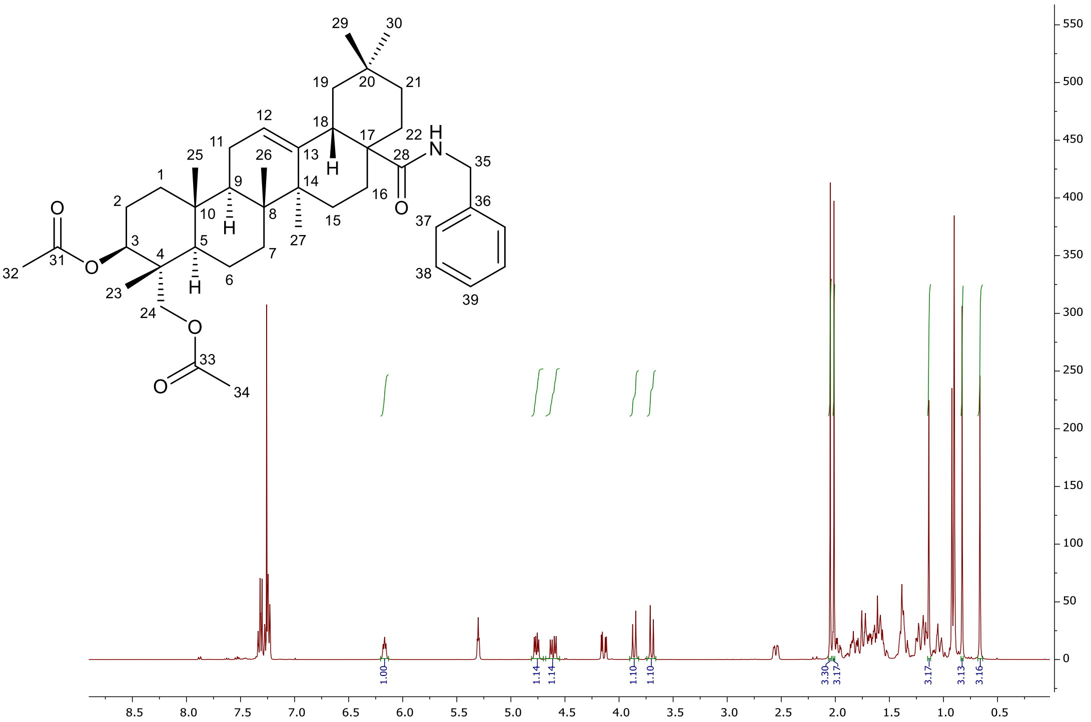

$^{13}$C NMR (100 MHz, $CDCl_3$)

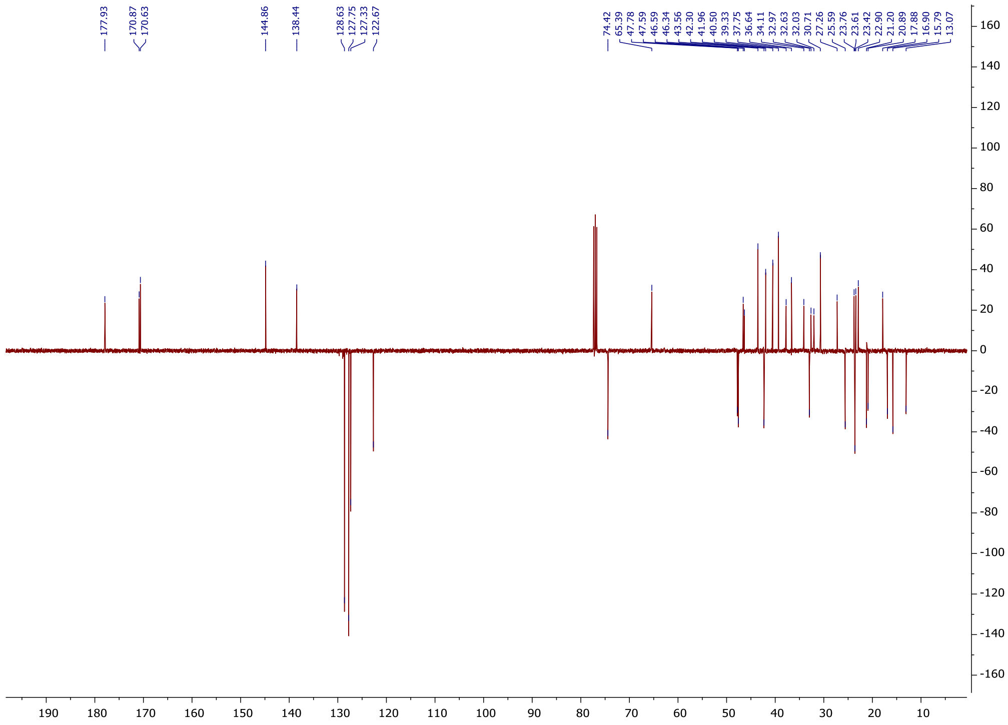

**Compound 12:**

$^{1}$H NMR (400 MHz, $CDCl_3$)

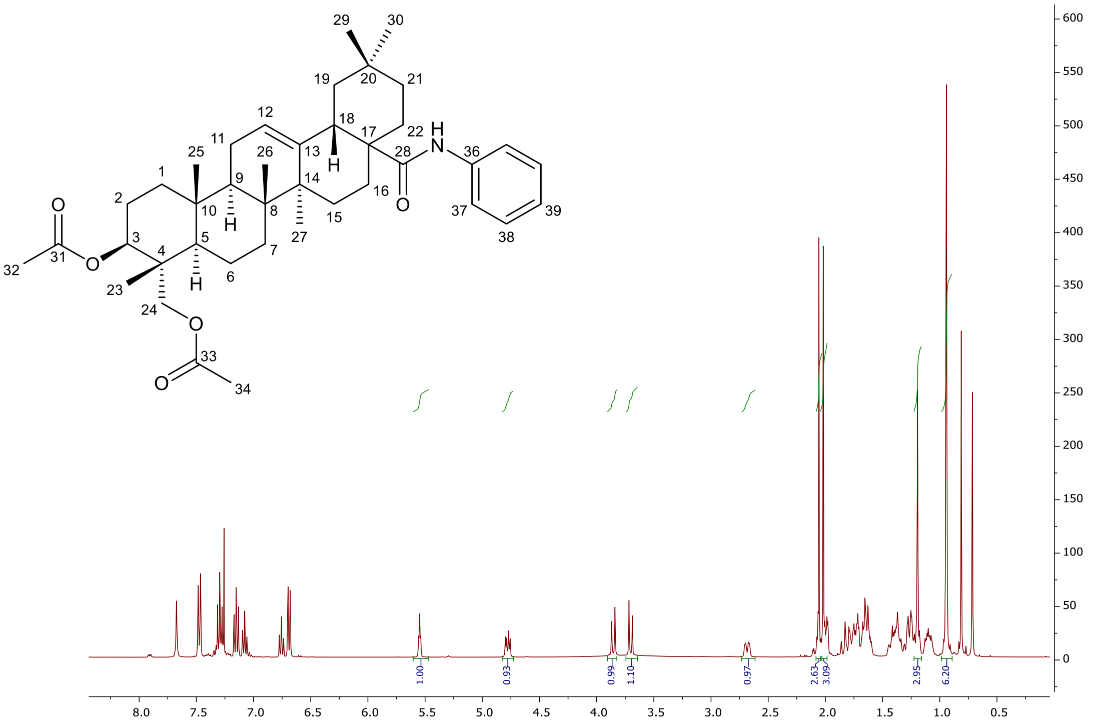


$^{13}$C NMR (100 MHz, $CDCl_3$)

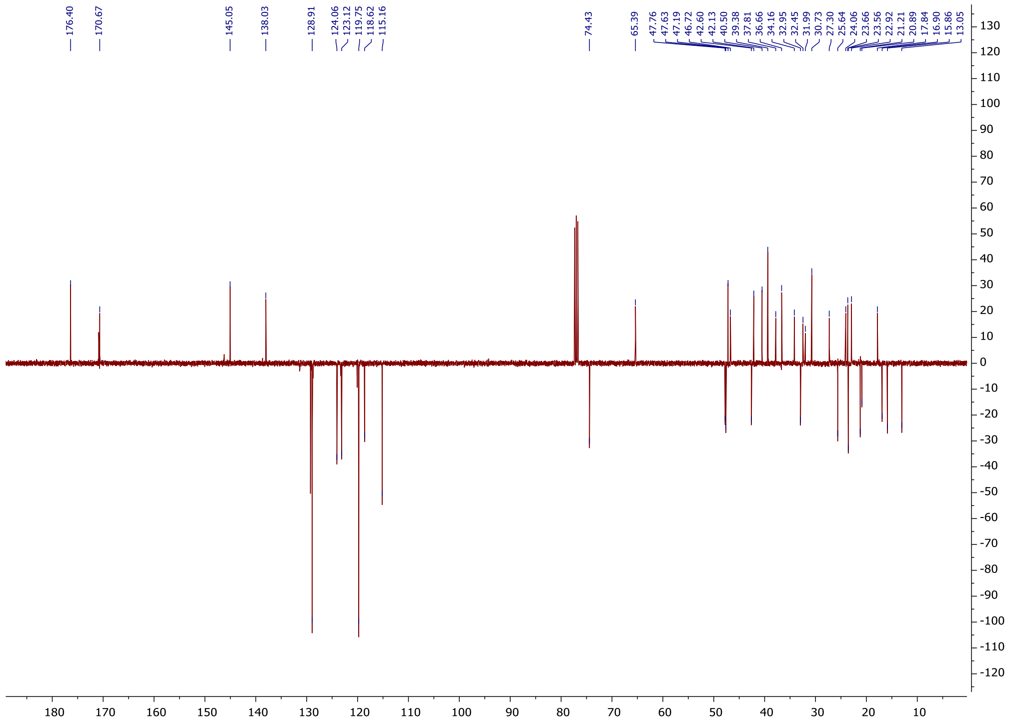

## Compound 13:

$^{1}$H NMR (500 MHz, $CDCl_3$)

7

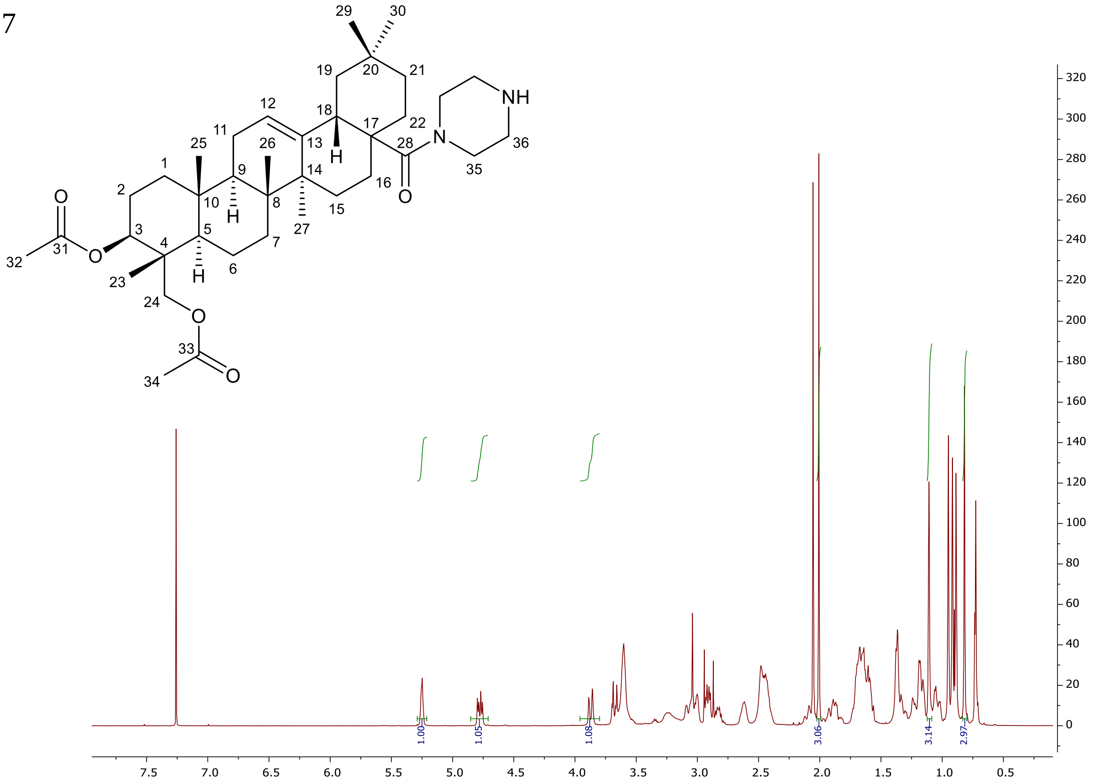


$^{13}$C NMR (125 MHz, $CDCl_3$)

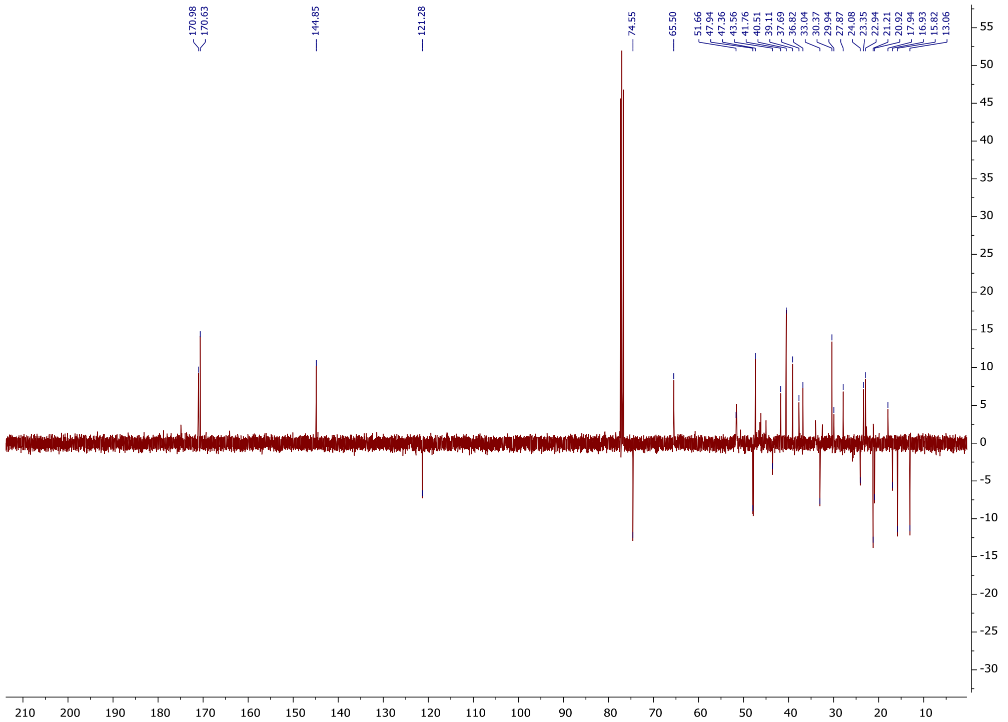

**Compound 14:**

$^{1}H$ NMR (400 MHz, $CDCl_3$)

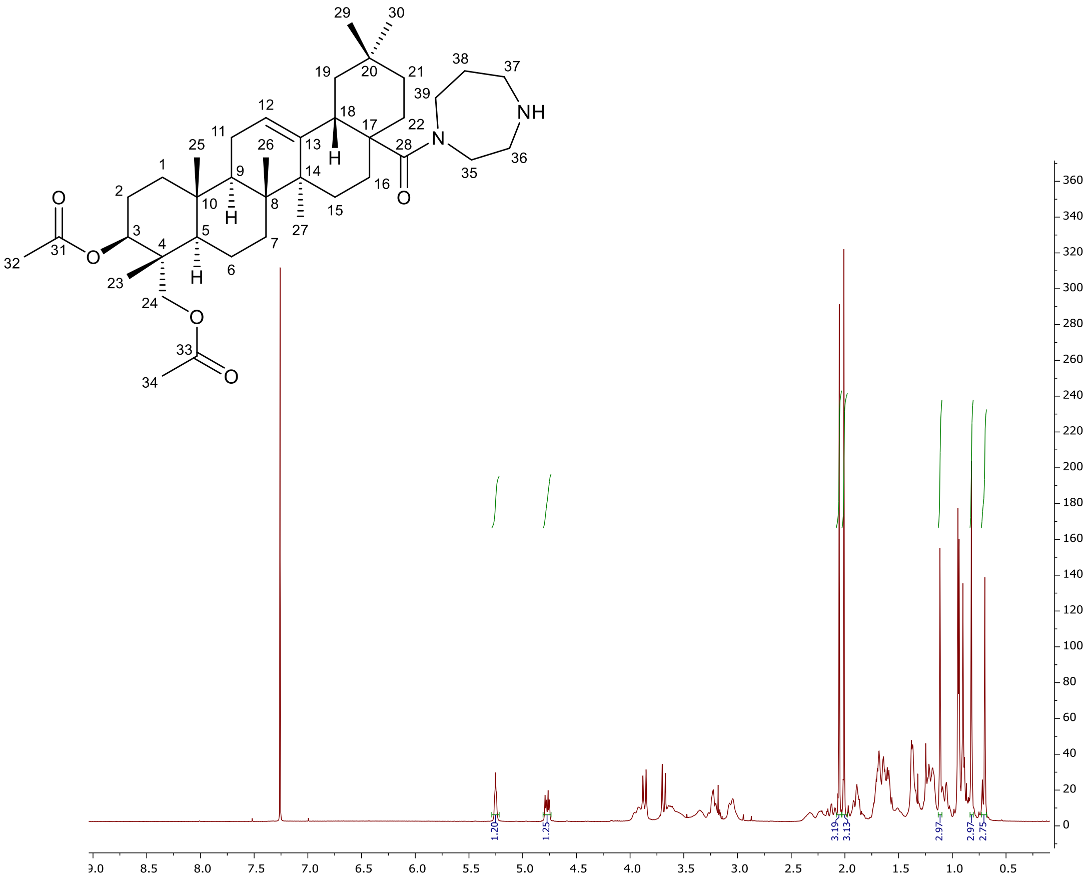


$^{13}C$ NMR (100 MHz, $CDCl_3$)

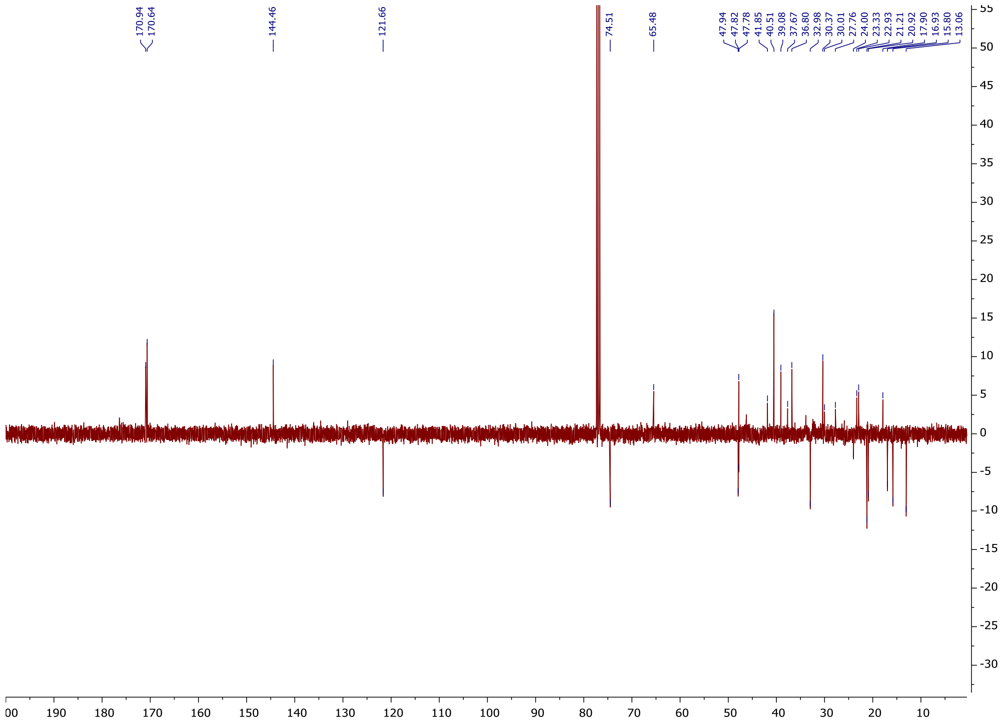

**Compound 15:**

$^{1}H$ NMR (400 MHz, $CDCl_3$)

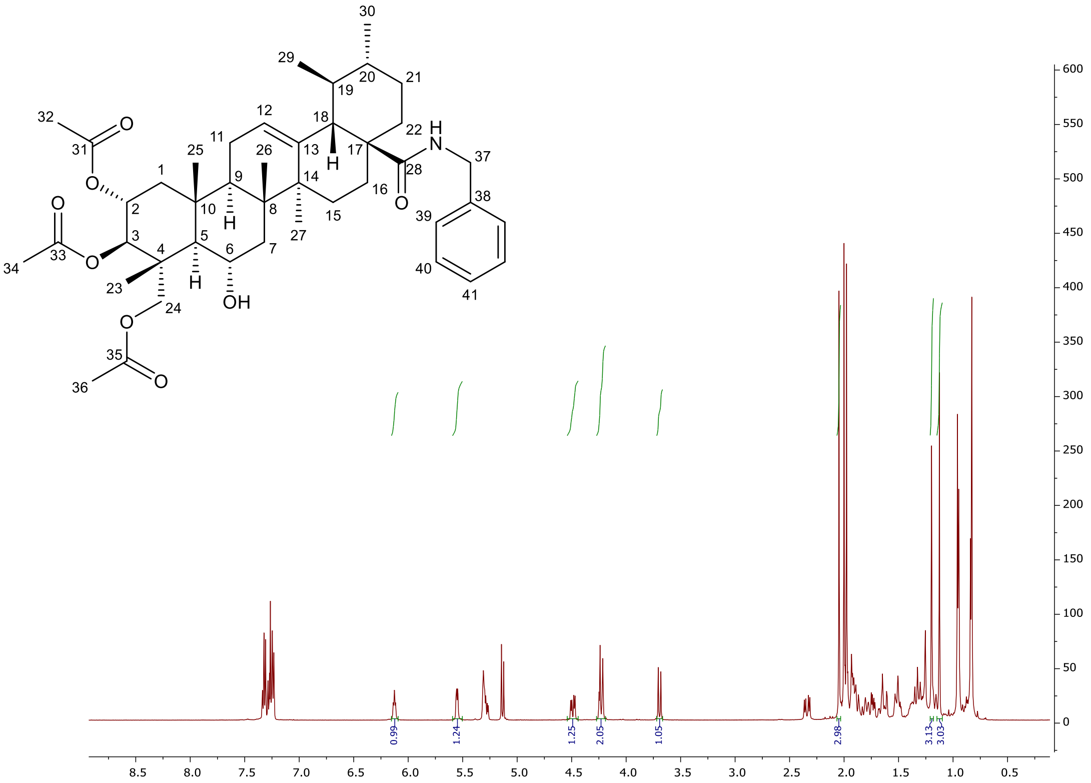


$^{13}C$ NMR (100 MHz, $CDCl_3$)

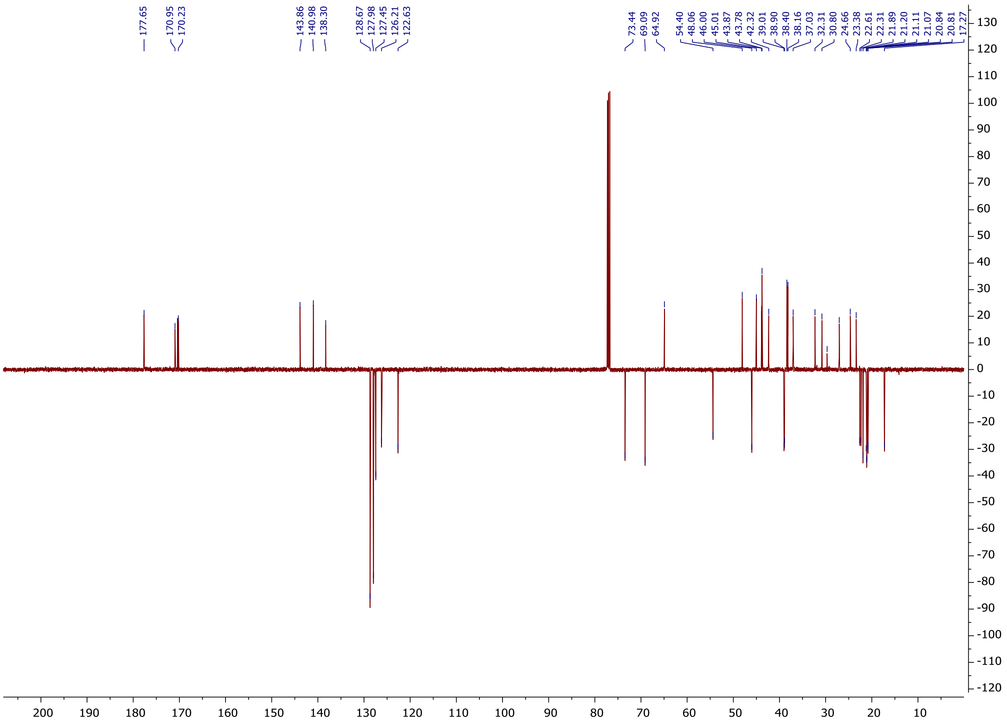

**Compound 16:**

$^1$H NMR (500 MHz, $CDCl_3$)

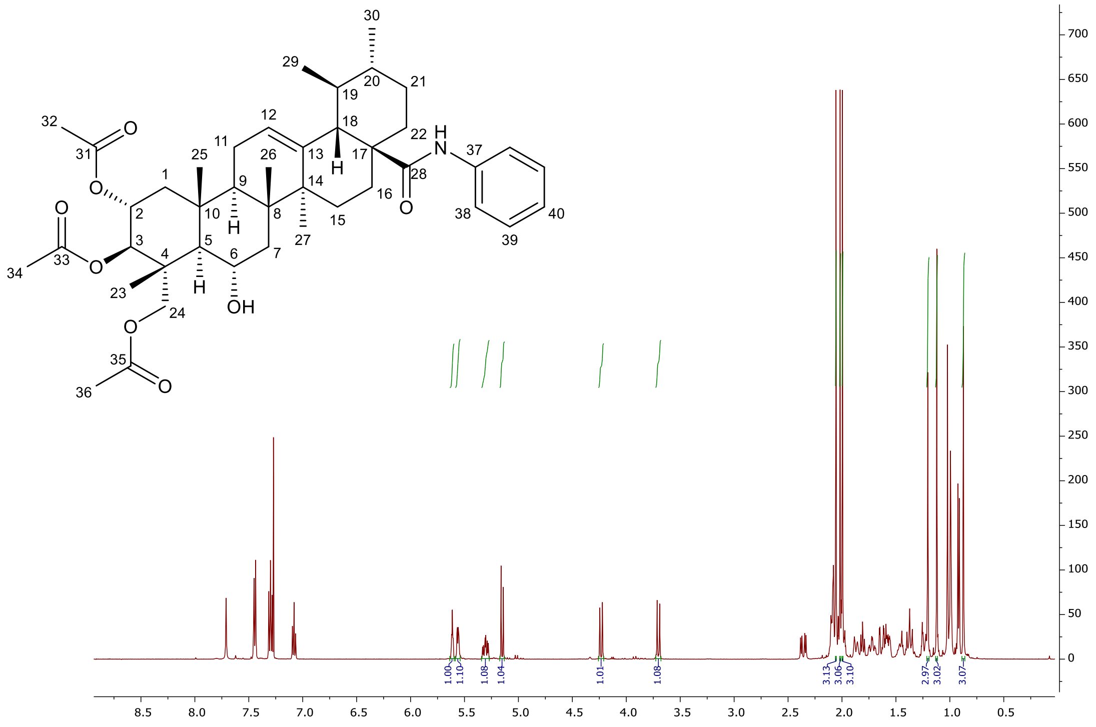


$^{13}$C NMR (125 MHz, $CDCl_3$)

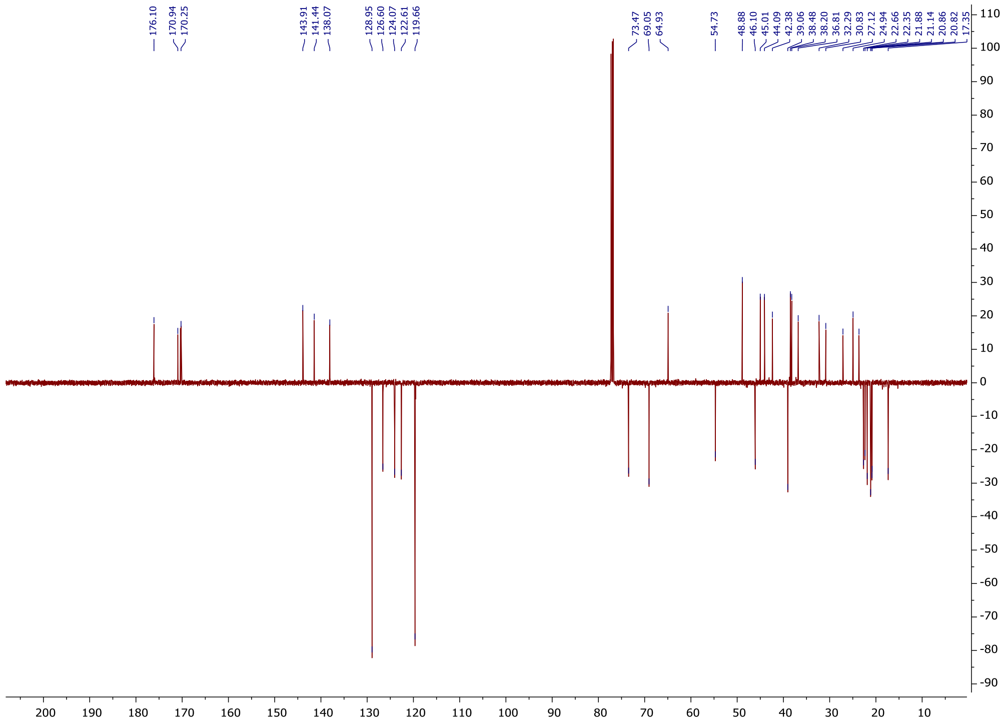

**Compound 17:**

$^{1}H$ NMR (500 MHz, $CDCl_3$)

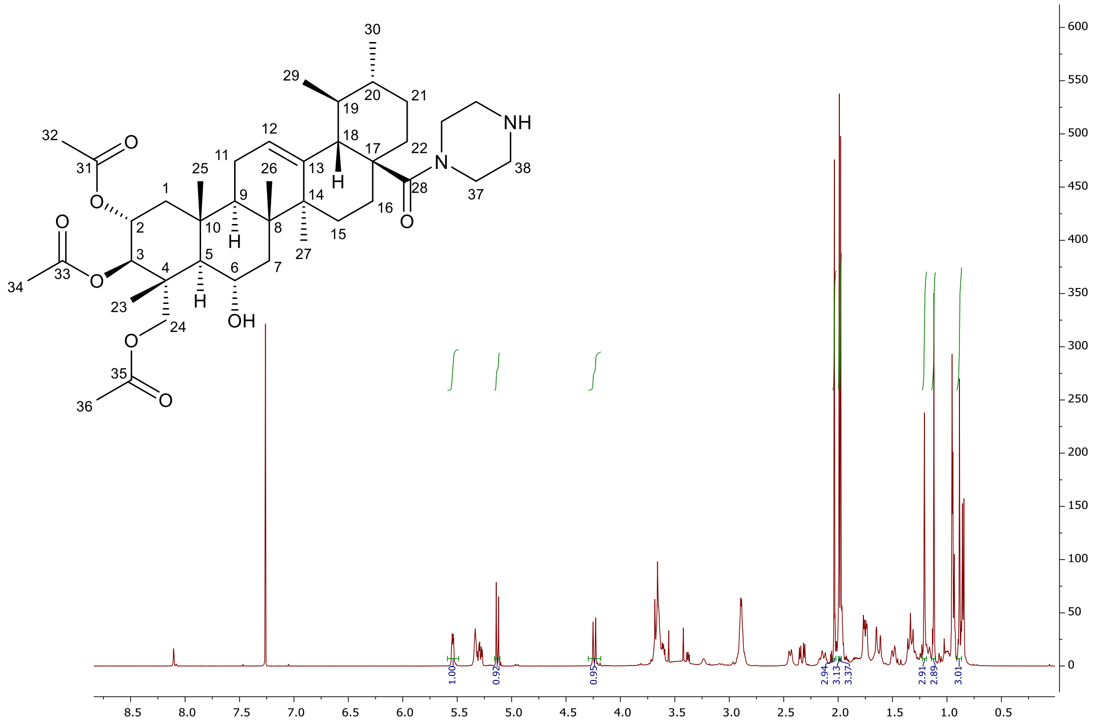


$^{13}C$ NMR (125 MHz, $CDCl_3$)

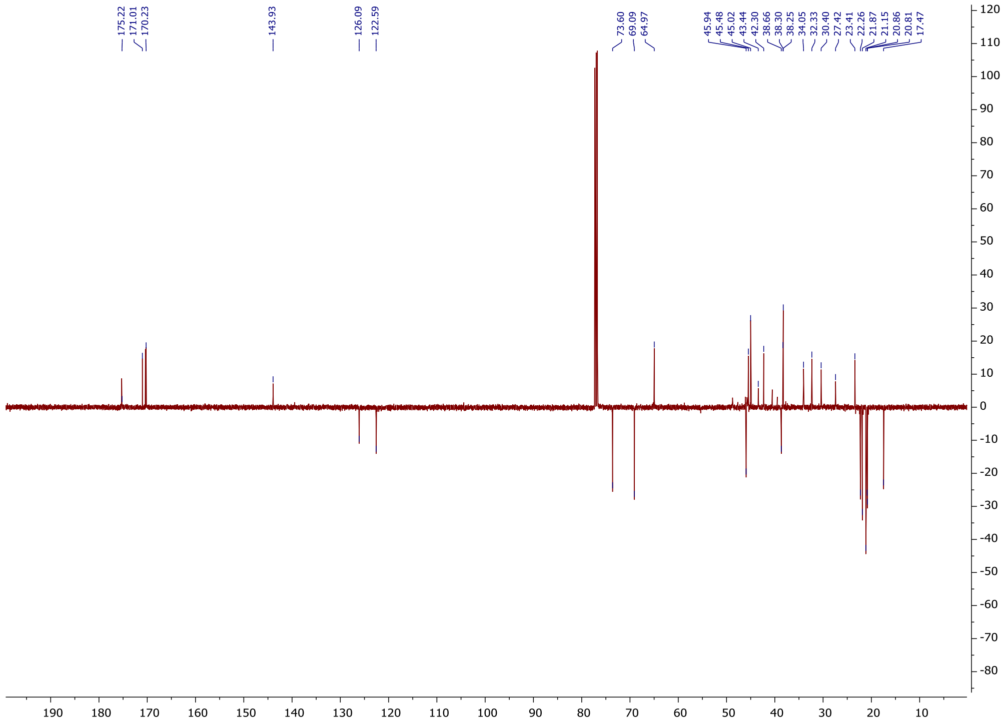

**Compound 18:**

$^{1}H$ NMR (500 MHz, $CDCl_3$)

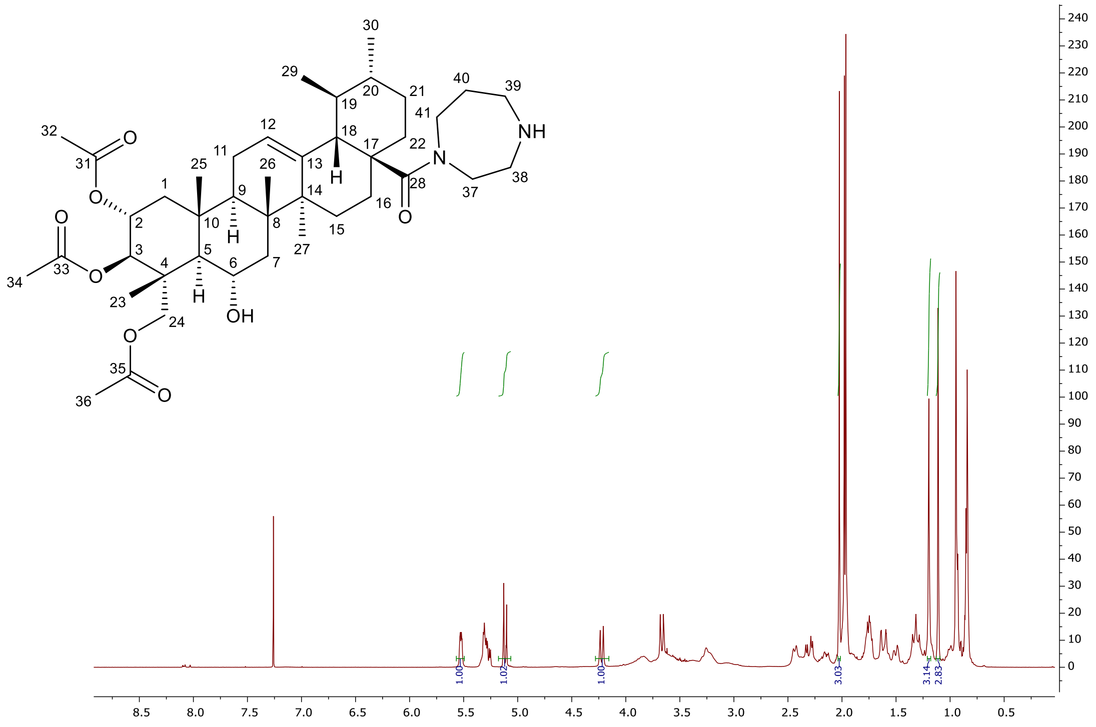


$^{13}C$ NMR (125 MHz, $CDCl_3$)

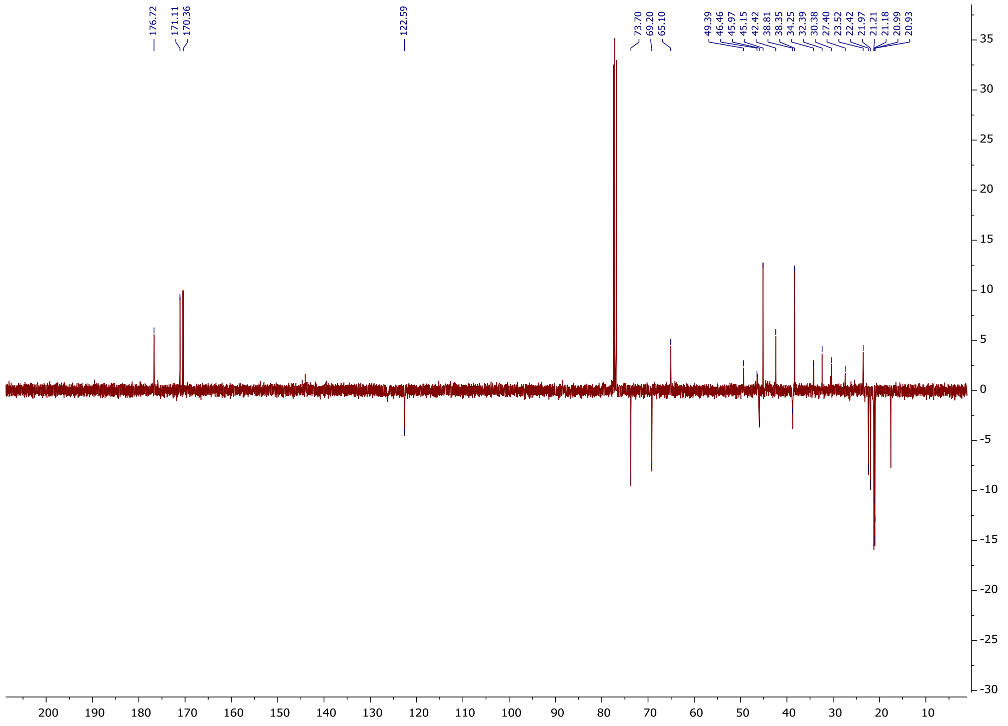

**Compound 19:**

$^{1}H$ NMR (400 MHz, $CDCl_3$)

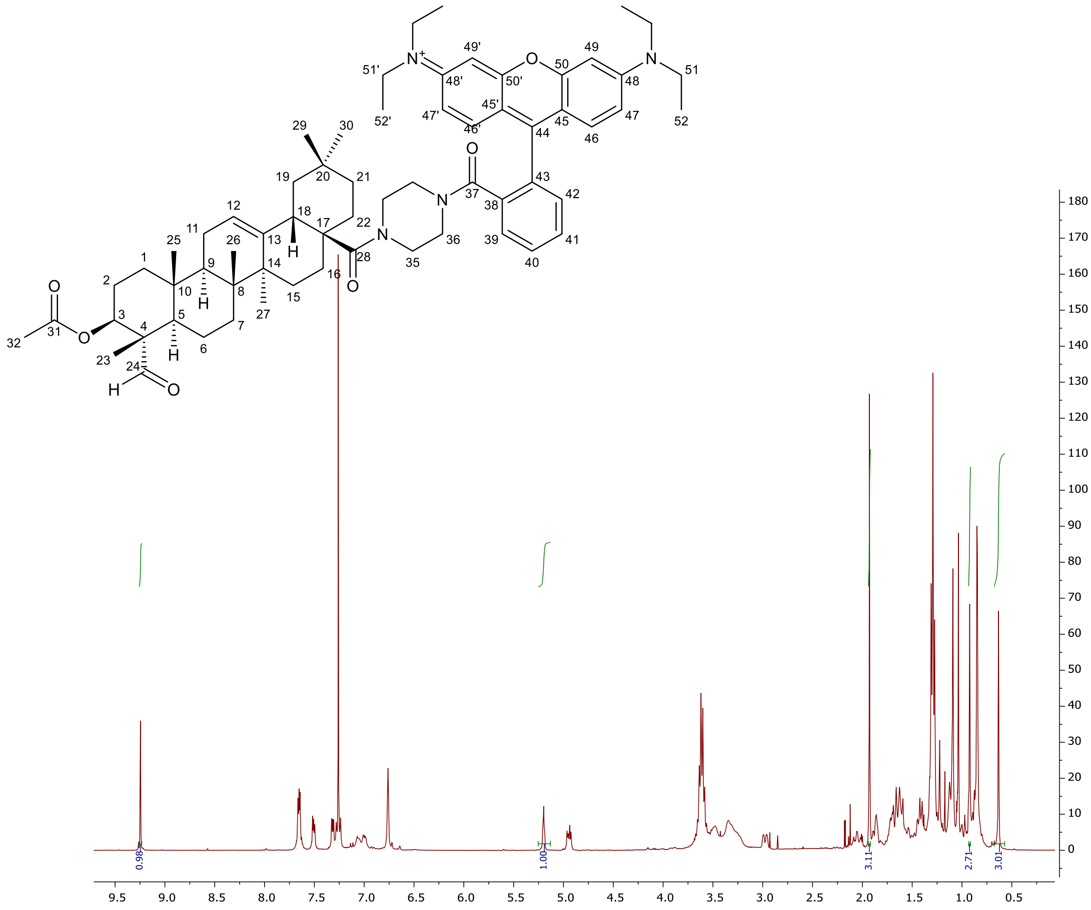

$^{13}C$ NMR (100 MHz, $CDCl_3$)

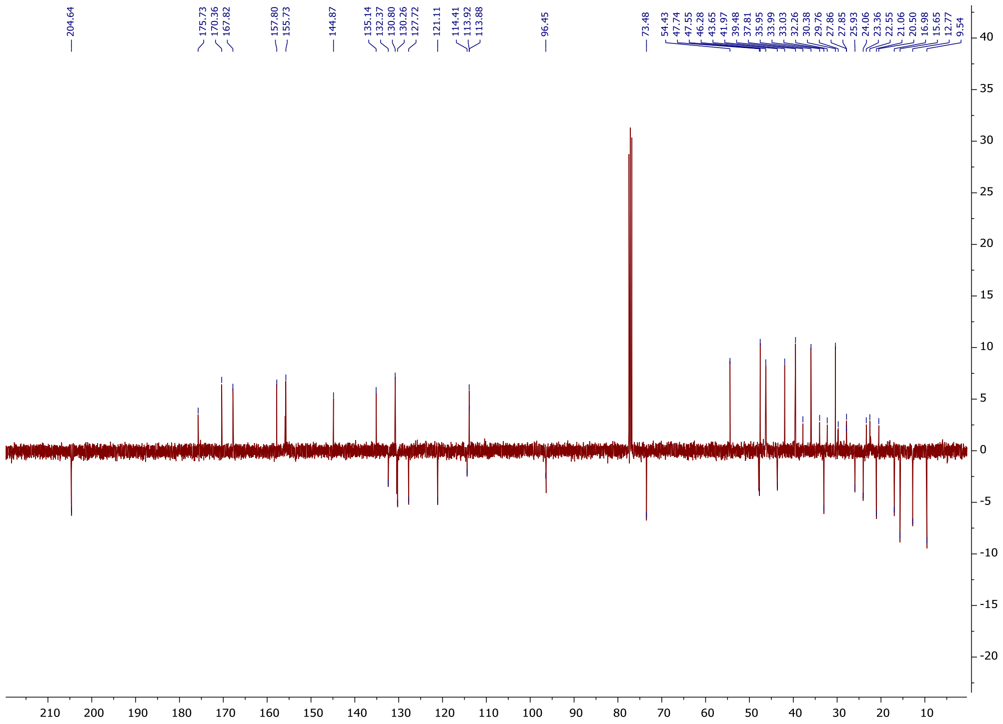

**Compound 20:**

$^{1}$H NMR (400 MHz, $CDCl_3$)

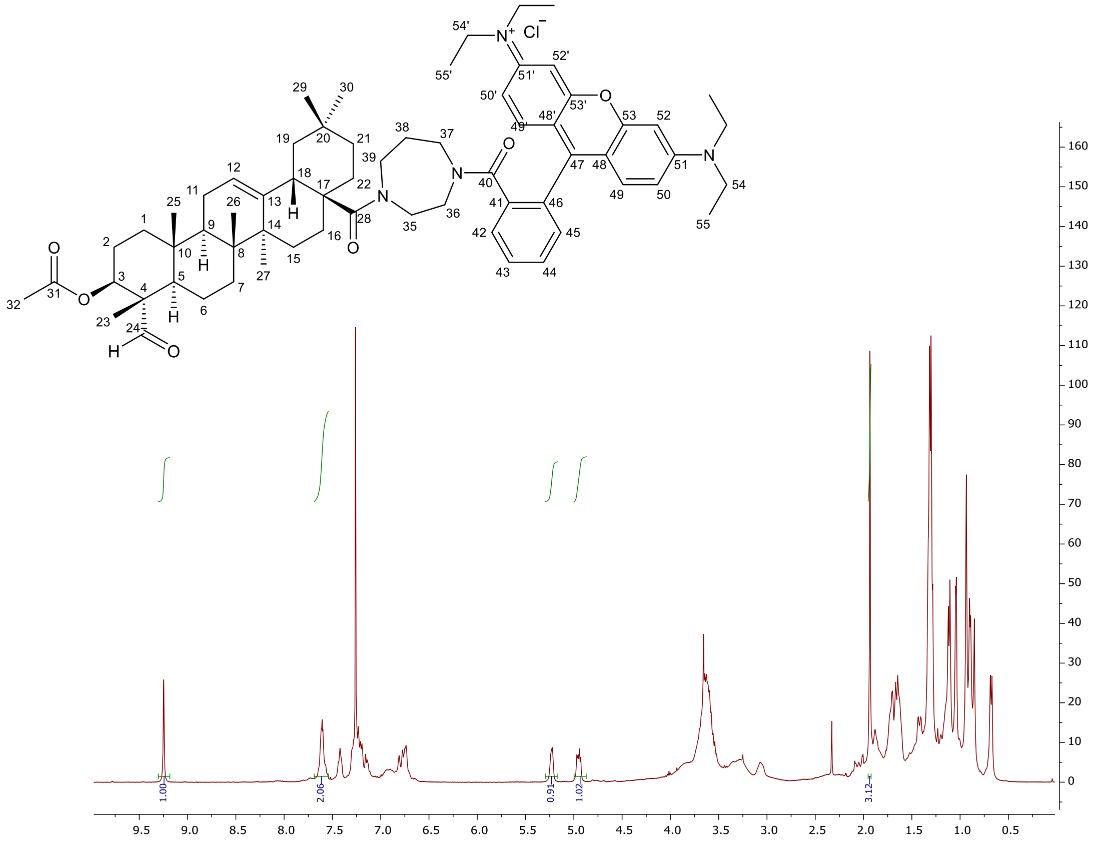

$^{13}$C NMR (100 MHz, $CDCl_3$)

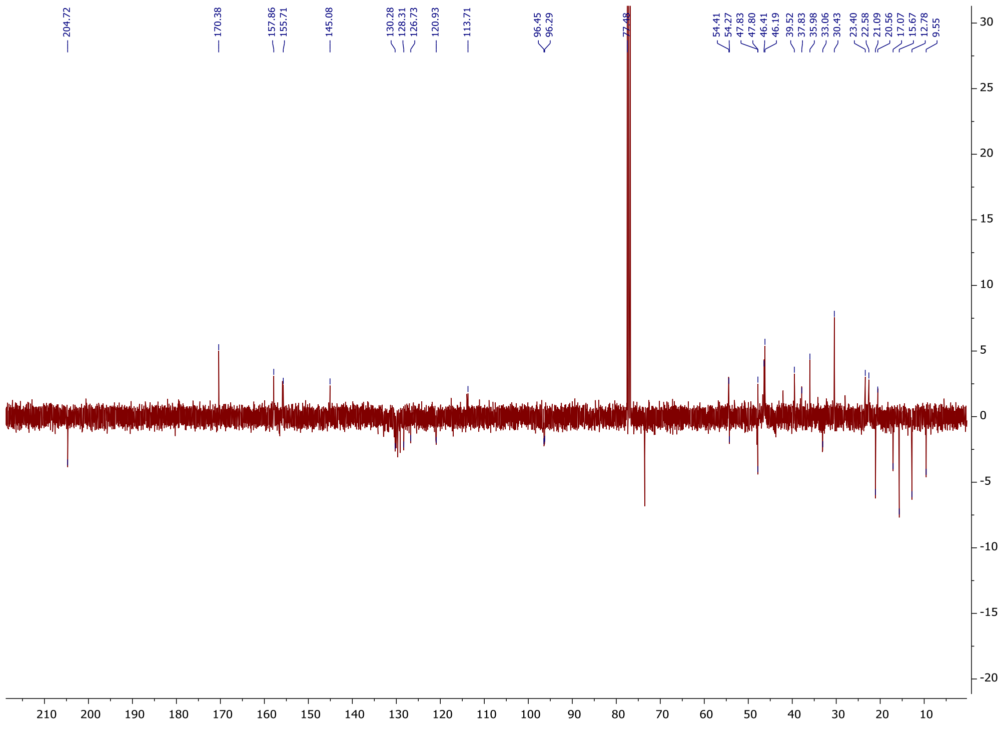

**Compound 21:**

$^{1}$H NMR (500 MHz, $CDCl_3$)

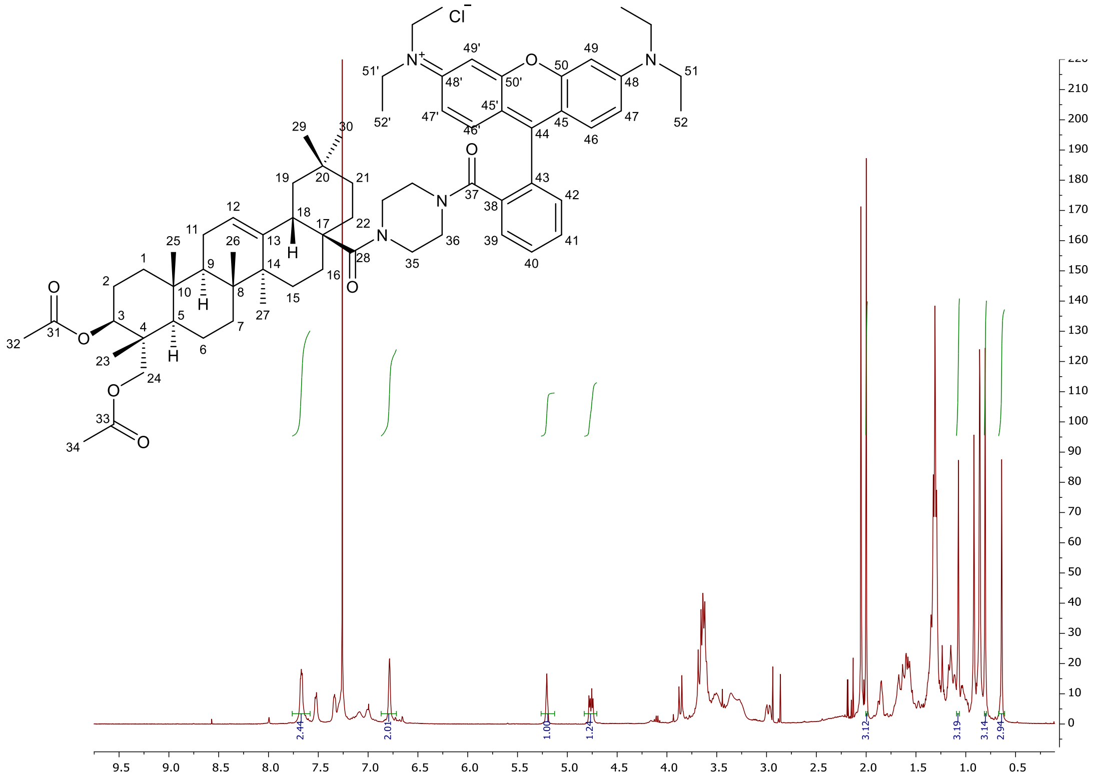

$^{13}$C NMR (125 MHz, $CDCl_3$)

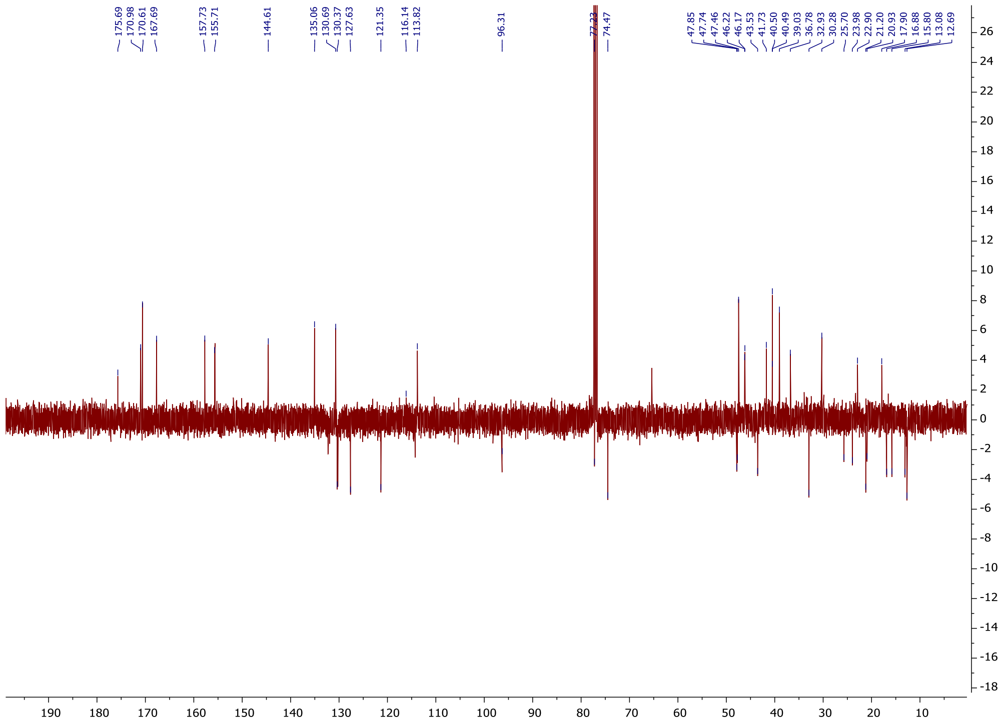

**Compound 22:**

$^{1}$H NMR (500 MHz, $CDCl_3$)

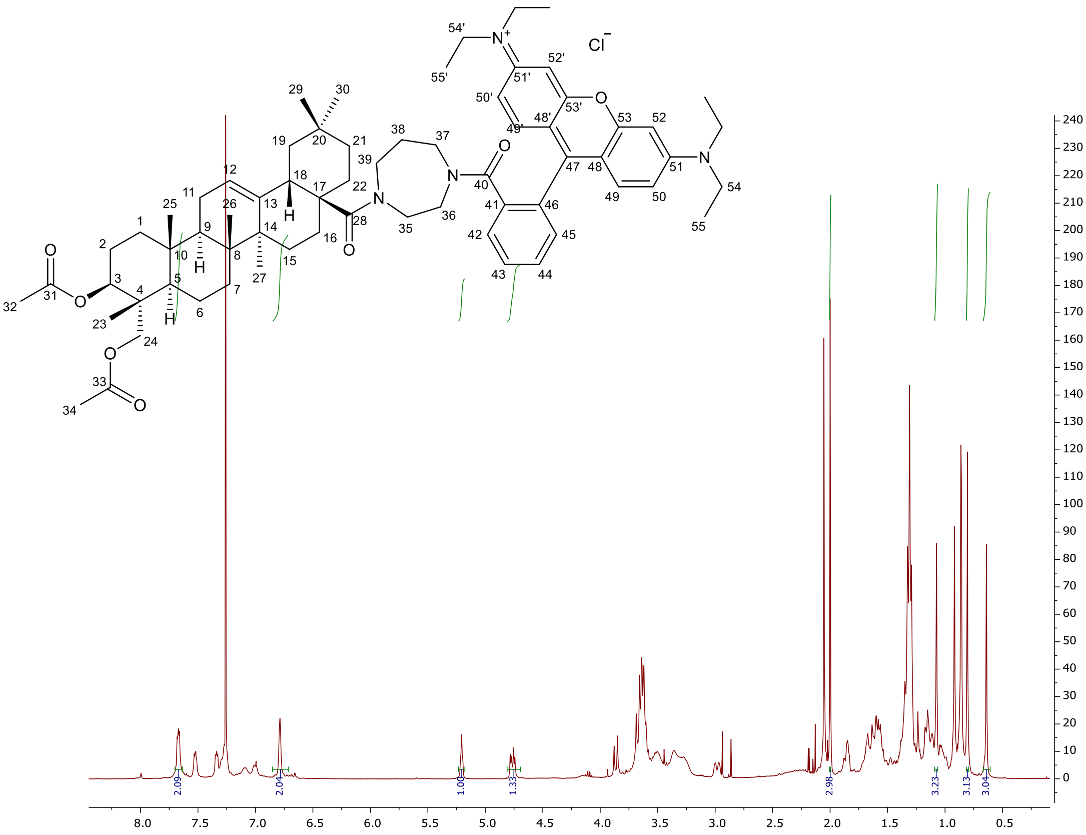


$^{13}$C NMR (125 MHz, $CDCl_3$)

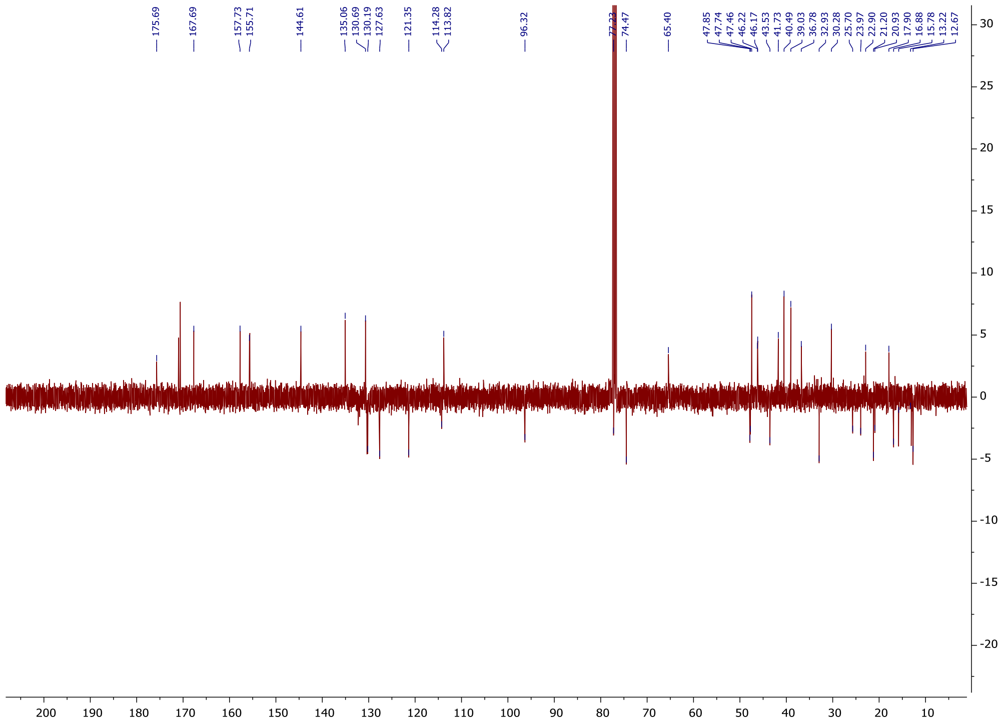

**Compound 23:**

$^{1}H$ NMR (500 MHz, $CDCl_3$)

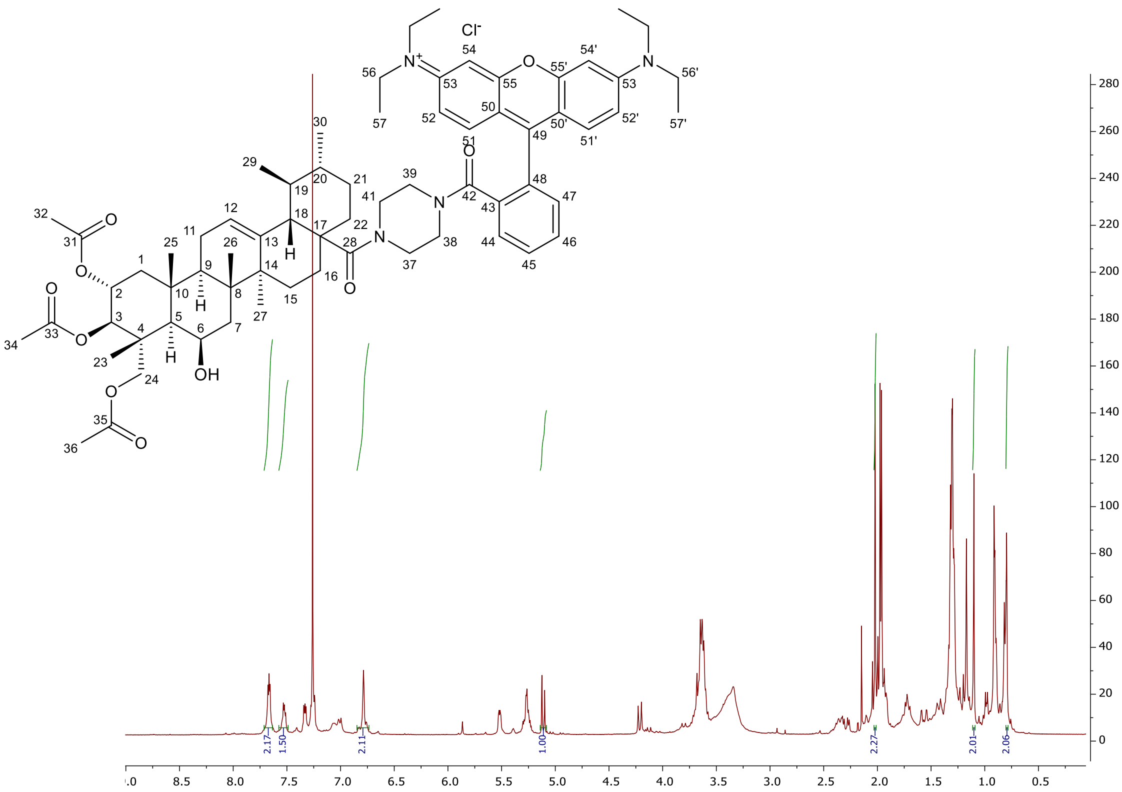


$^{13}C$ NMR (125 MHz, $CDCl_3$)

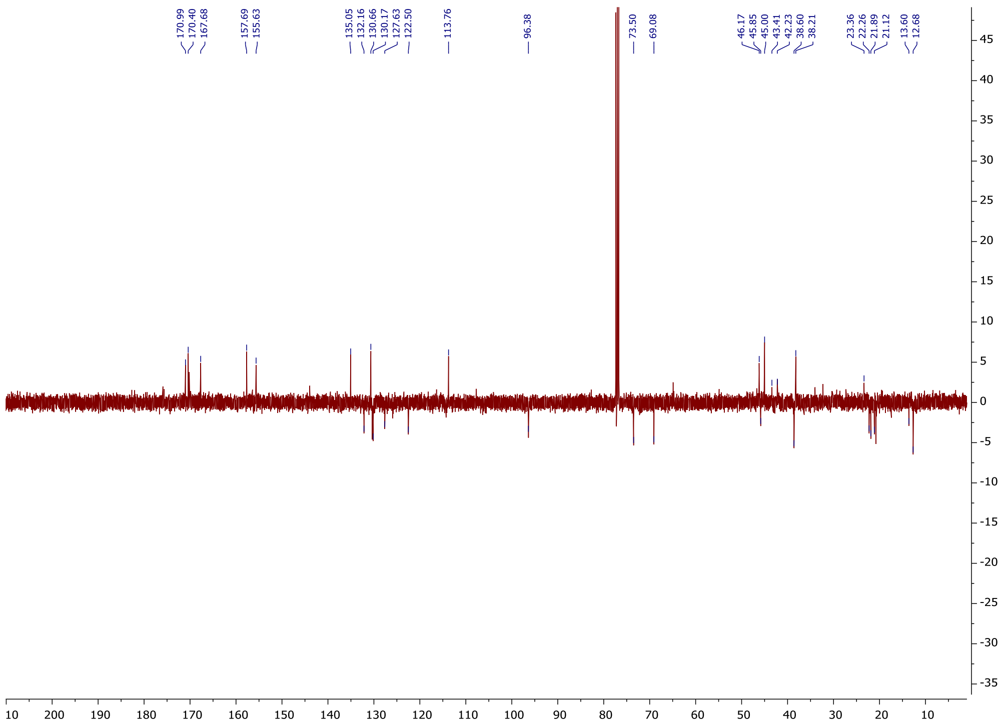

**Compound 24:**

$^1$H NMR (500 MHz, $CDCl_3$)

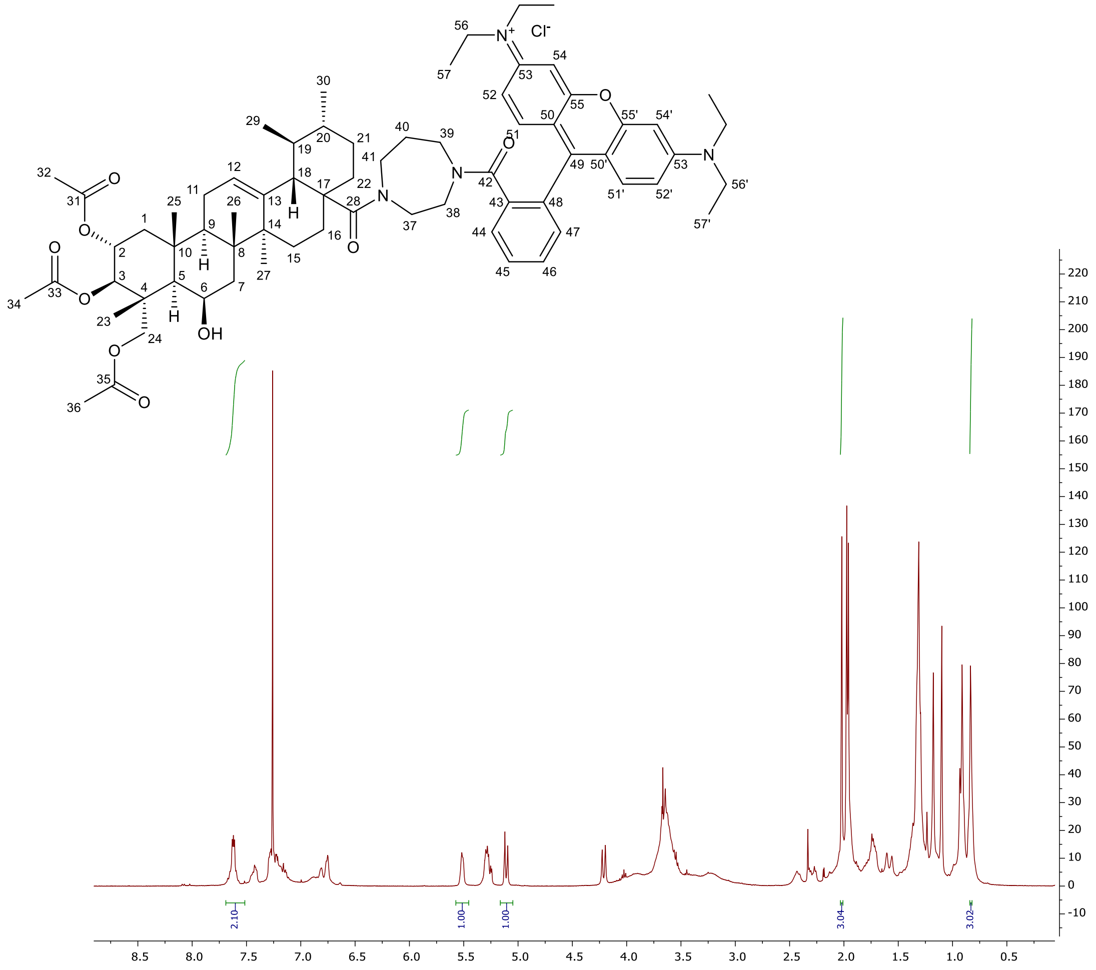


$^{13}$C NMR (125 MHz, $CDCl_3$)

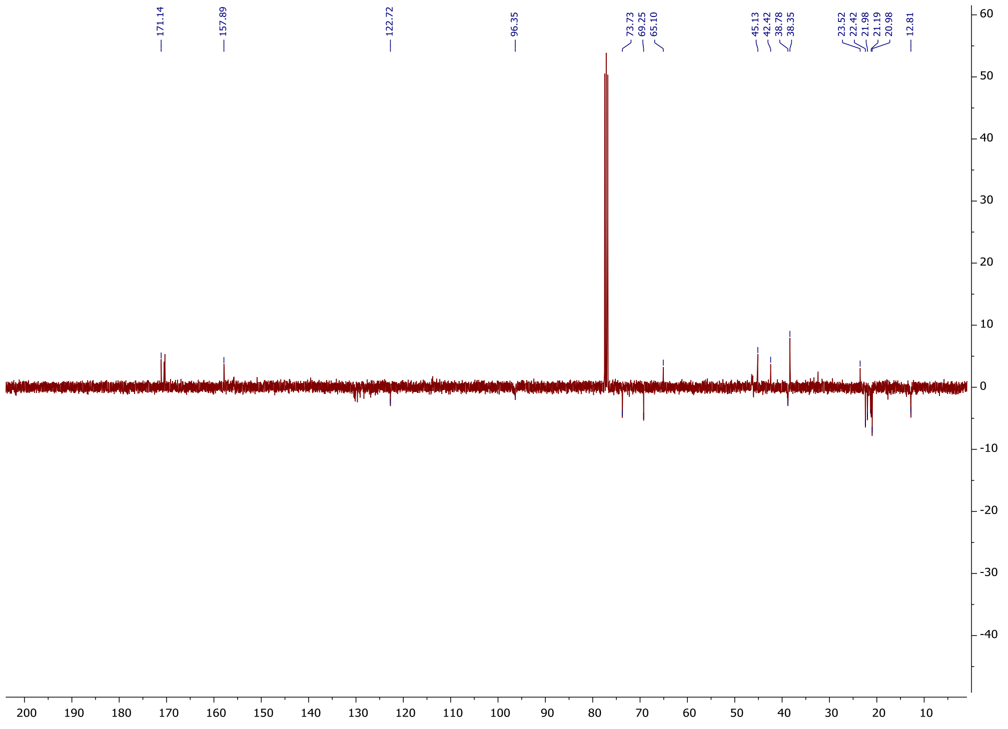

Supplement: Supplementary file 1 [file ijms-23-04362-s001.zip › ijms-1671697-supplementary.pdf]
